# A consensus framework map of durum wheat (*Triticum durum* Desf.) suitable for linkage disequilibrium analysis and genome-wide association mapping

**DOI:** 10.1186/1471-2164-15-873

**Published:** 2014-10-07

**Authors:** Marco Maccaferri, Maria Angela Cane’, Maria C Sanguineti, Silvio Salvi, Maria C Colalongo, Andrea Massi, Fran Clarke, Ron Knox, Curtis J Pozniak, John M Clarke, Tzion Fahima, Jorge Dubcovsky, Steven Xu, Karim Ammar, Ildikó Karsai, Gyula Vida, Roberto Tuberosa

**Affiliations:** Department of Agricultural Sciences (DipSA), University of Bologna, Viale Fanin 44, 40127 Bologna, Italy; Società Produttori Sementi Bologna (PSB), 40050 Argelato (BO), Italy; Semiarid Prairie Agricultural Research Centre, Agriculture and Agri-Food Canada, P.O. Box 1030, Swift Current, SK S9H 3X2 Canada; Crop Development Centre and Department of Plant Sciences, University of Saskatchewan, 51 Campus Drive, Saskatoon, SK S7N 5A8 Canada; Department of Evolutionary and Environmental Biology, Institute of Evolution, Faculty of Science and Science Education, University of Haifa, Mt. Carmel, Haifa, 31905 Israel; Department of Plant Sciences, University of California, Davis, CA 95616 USA; Howard Hughes Medical Institute, Chevy Chase, MD 20815 USA; USDA/ARS Cereal Crops Research Unit, NCSL 1605 Albrecht Blvd. N, Fargo, ND 58102 USA; International Maize and Wheat Improvement Center (CIMMYT), Apartado Postal 6-641, 06600, Mexico D.F., Mexico. Carretera Mexico-Veracruz KM. 45, 56130 Texcoco, Mexico; Center for Agricultural Research, Hungarian Academy of Sciences (ARI-HAS), Brunszvik U. 2, H-2462, Martonvasar, Hungary

**Keywords:** *Triticum durum* Desf, Consensus map, Linkage disequilibrium, Genome-wide association mapping, Heading date, QTL

## Abstract

**Background:**

Durum wheat (*Triticum durum* Desf.) is a tetraploid cereal grown in the medium to low-precipitation areas of the Mediterranean Basin, North America and South-West Asia. Genomics applications in durum wheat have the potential to boost exploitation of genetic resources and to advance understanding of the genetics of important complex traits (e.g. resilience to environmental and biotic stresses). A dense and accurate consensus map specific for *T. durum* will greatly facilitate genetic mapping, functional genomics and marker-assisted improvement.

**Results:**

High quality genotypic data from six core recombinant inbred line populations were used to obtain a consensus framework map of 598 simple sequence repeats (SSR) and Diversity Array Technology^®^ (DArT) anchor markers (common across populations). Interpolation of unique markers from 14 maps allowed us to position a total of 2,575 markers in a consensus map of 2,463 cM. The *T. durum* A and B genomes were covered in their near totality based on the reference SSR hexaploid wheat map. The consensus locus order compared to those of the single component maps showed good correspondence, (average Spearman’s rank correlation rho *ρ* value of 0.96). Differences in marker order and local recombination rate were observed between the durum and hexaploid wheat consensus maps. The consensus map was used to carry out a whole-genome search for genetic differentiation signatures and association to heading date in a panel of 183 accessions adapted to the Mediterranean areas. Linkage disequilibrium was found to decay below the *r*^2^ threshold = 0.3 within 2.20 cM, on average. Strong molecular differentiations among sub-populations were mapped to 87 chromosome regions. A genome-wide association scan for heading date from 27 field trials in the Mediterranean Basin and in Mexico yielded 50 chromosome regions with evidences of association in multiple environments.

**Conclusions:**

The consensus map presented here was used as a reference for genetic diversity and mapping analyses in *T. durum*, providing nearly complete genome coverage and even marker density. Markers previously mapped in hexaploid wheat constitute a strong link between the two species. The consensus map provides the basis for high-density single nucleotide polymorphic (SNP) marker implementation in durum wheat.

**Electronic supplementary material:**

The online version of this article (doi:10.1186/1471-2164-15-873) contains supplementary material, which is available to authorized users.

## Background

Cultivated tetraploid wheat (durum wheat, *Triticum durum* Desf.) is genetically differentiated from hexaploid wheat (*Triticum aestivum* L.) by as little as ca. 10,000 years of evolution. Both species derived from domesticated emmer (*Triticum dicoccum* Schrank) through a rather long human-driven selection process, including distinct and sequential domestication bottlenecks and continuous gene flow from wild emmer (*Triticum dicoccoides* (Körn. ex Asch. Graebner) Schweinf) [1]. In the past two decades, molecular genetics and genomics of durum wheat, including breeding applications such as marker-assisted selection (MAS), have largely relied on the molecular tools specifically developed for hexaploid wheat [2–5]. The restriction fragment length polymorphisms (RFLPs) and the PCR-based molecular markers such as the simple sequence repeats (SSRs), amplified fragment length polymorphisms (AFLPs), and sequence tagged sites (STSs) developed for hexaploid wheat performed rather efficiently in tetraploid wheat based on the commonalities between the two shared A- and B-genomes [6–8]. However, marker’s polymorphism information content had to be re-assessed directly in the durum wheat germplasm [9, 10].

In the 2000’s, intra- and inter-specific durum wheat and durum wheat × wild emmer genetic linkage maps were developed mainly using SSR loci [11, 12] as well as the microarray-based Diversity Array Technology (DArT^®^) markers [13–15].

Integration of the SSR-based linkage map data into consensus maps was first pursued in hexaploid wheat by Somers et al. [16] and Crossa et al. [17], and then in durum wheat by Trebbi et al. [18], Marone et al. [19] and Letta et al. [20]. In durum wheat, the limited number of maps available to these studies has not yet allowed for full coverage of the genome, an important prerequisite for genome-wide association mapping, meta-analysis and positional cloning. The most recently published consensus map specific to tetraploid wheat [19] is composed of 1,898 loci arranged in 27 linkage groups, a number that exceeds the 14 nuclear chromosomes of this species.

The hexaploid wheat SSR-based consensus map (Ta-SSR-2004) by Somers et al. [16] has been widely used as a reference for wheat genomics studies for more than a decade. In hexaploid wheat, several mapping studies were published using SSR and DArT technologies, revealing major genes and QTLs for grain yield, yield components, adaptive traits, response to abiotic and biotic stresses and quality (reviewed in [3, 21–23]). Collectively, these studies laid the foundation for MAS applications in wheat (http://maswheat.ucdavis.edu). Within this dynamic scenario, the availability of a high-quality consensus map more exhaustively covering the entire genome is a valuable asset for genetic studies and breeding applications. Furthermore, powerful association mapping (AM) and meta-QTL analysis require reference genetic maps of a quality standard higher than that commonly adopted for coarse mapping in bi-parental populations. AM and meta-QTL analysis are being increasingly adopted in wheat genomics (162 records for association mapping studies and 7 records for meta- QTL analysis in NCBI-PubMed), including durum wheat [20, 24–27].

The development of integrated maps were approached based on: (*i*) ‘consensus’ mapping, where *de novo* maps are produced by merging all the single segregation data-sets available to develop a novel unified map, (*ii*) ‘interpolation’ mapping (or ‘projection-based’ mapping), where integrated maps are built by regressing the single component map marker positions on an initial reference map that provides the framework for sequential interpolation. The latter method is computation-and-time efficient but relies on the availability of an accurate reference with strong prerequisites such as comprehensive genome coverage and proven representativeness.

Consensus mapping has the potential to overcome the major limitations typical of mapping information based on single maps, such as local aberrations in crossover rates [28, 29] and presence of chromosome regions with locally low marker density/lack of polymorphism due to the occurrence of identity by descent [30]. Moreover, consensus mapping provides more accurate mapping information thanks to the higher number of progenies considered. Several software packages were developed for consensus mapping, from the early ones that pool single component segregation data and calculate consensus maps based on the assumption of homogeneity of marker order and recombination rate across populations, such as Joinmap [31] and Carthagene [32], to others that implement graph-theoretic models with marker removal in case of conflicting orders (e.g. MergeMap by [33]) or attempt to define the optimal consensus order of shared markers by a synchronous optimization of the local orders in single component maps (e.g. Multipoint Consensus, [34]).

In this study, segregation data from six core mapping populations of durum wheat were analysed based on a common procedure with stringent quality criteria. Following identification and removal from the global computation of chromosome regions showing local deviations from the homogeneity assumptions, mainly identified in single component maps only, the segregation data from markers shared among populations were pooled in Carthagene to obtain a consensus framework map that proved to be highly robust compared to the single component maps. The value of this consensus map, enriched in density by the interpolation of the unique markers, was assessed through the identification of signatures of genetic differentiations among sub-populations of the durum wheat germplasm and the identification of QTLs controlling heading date in the Mediterranean cultivation areas at the genome-wide scale.

## Results

### Linkage maps from the individual populations (component maps)

The genotypic data of 14 mapping populations of tetraploid wheat (*2n* = AABB), either recombinant inbred lines (RILs) or double haploids (DHs), were made available by collaborating Institutions or were downloaded from the GrainGenes database. The genotypic data were used to re-construct the maps of the original populations following a common mapping procedure. The reconstructed maps are thereafter reported as *component maps*. Details of the parental accessions, cumulative genetic distances and marker composition for each of the component maps are reported in Table 1.

**Table 1:** **Component maps used for consensus mapping and map integration: markers and map features**

Cross		Population type	Population size	Markers and map features							
Acronym	Detailed	DH/RIL/F _2_	(no.)	SSR (no.)	DArT/AFLP (no.)	SNP/STS (no.)	Biochemical/morphological loci (no.)	Total marker (no.)	Linkage group (no.)	Total length (cM)	Inter-marker distance (cM/marker)
**Mapping populations used for consensus framework mapping**											
Kf × Sv	Kofa × Svevo	RIL	249	303	0	8	0	311	25	1,258.7	4.04
Co × Ll	Colosseo × Lloyd	RIL	176	197	393	118	1	709	21	1,810.3	2.55
Mr × Cl	Meridiano × Claudio	RIL	181	202	653	86	0	941	25	1,940.4	2.06
Sm × Lv	Simeto × Levante	RIL	180	170	403	2	0	575	27	1,514.7	2.63
Ln × G18-16	Langdon × G18-16	RIL	93	127	171	2	1	301	21	1,536.7	5.10
Kf × UC	Kofa × UC1113	RIL	152	225	0	20	2	247	27	787.4	3.18
Average								514	24	1,474.7	3.26
**Mapping population used for marker interpolation only**											
Gl × Dm	Gallareta × Demetra	DH	127	19	128	0	0	147	15	1,017.3	6.92
DT707 × DT696	DT707 × DT696	DH	127	68	68	0	0	136	18	861.1	6.33
DT712 × Bl	DT712 × Blackbird	DH	89	392	0	0	0	392	23	1,848.2	4.71
Lb × P749	Lebsock × PI94749	DH	146	239	0	0	1	240	16	1,463.4	6.09
Sv × Cc	Svevo × Ciccio	RIL	120	165	0	0	4	169	24	1,214.8	7.19
PDW1216xMvTD10-98	PDW1216 x MvTD10-98	RIL	182	0	440	0	0	440	18	984.3	2.23
Average								254	19	1,231.5	5.57
**Mapping population used for marker interpolation only, no segregation data available**											
PDW × Bh	PDW233 × Bhalegaon 4	RIL	140	128	0	18	12	158	18	1,839.1	11.64
Lt × Pr	Latino × Primadur	F_2_	121	45	66	9	0	120	6	422.3	3.51

For each map, the cross, population type (recombinant inbred line, RIL or double haploid, DH ), number of mapped markers listed by marker type (simple sequence repeat, SSR; Diversity Array Technology, DArT; amplified length polymorphisms, AFLP; single nucleotide polymorphisms, SNPs; sequence tagged sites, STSs; Mendelian loci), number of linkage groups, total map length (cM) and average inter-marker distance (cM/marker) are reported.

A core-set of six RIL-based component maps was selected to obtain a robust framework map subsequently used for interpolation of unique markers. All but one of these six RIL populations were obtained from intraspecific *T. durum* crosses, with parental lines chosen among diverse groups of the elite durum germplasm (Italian, CIMMYT, North and South Western USA breeding groups, as reported in Table 1). One of the six populations included in the core set was obtained from the cross of Langdon × *T. dicoccoides* (G18-16), the wild progenitor of *T. durum*. In total, the core set of RIL populations included 1,031 lines. The single maps were constituted by 21 to 27 linkage groups, with total length ranging from 788 (Ln × G18-16) to 1,940 cM (Mr × Cl), with an average of 1,475 cM, and inter-marker distance varying from 2.6 (Mr × Cl) to 5.10 (Ln × G18-16) cM, with an average of 3.26 cM/marker.

Eight additional populations were used for marker interpolation. Four of the populations included in this second set were DHs obtained from Agriculture and Agrifood Canada, University of Saskatchewan and USDA. Three additional populations were recombinant inbred and one was a population of F_2:3_ progenies (Latino × Primadur, [35]). For six out of these eight populations the original segregation data were available and thus the linkage maps were reconstructed according to the common procedure used for the common maps. The two PDW233 × Bhalegaon 4 [36] and Latino × Primadur maps [35], for which the original segregation data were not available, were inspected for their homogeneity in marker distribution and inter-marker distances as compared to the *component* maps before to integrate their map information data into the consensus map. The marker density of all of these eight maps was inferior to that reached in the six core set RIL populations (Table 1), while retaining a good level of genome coverage (total map length comprised between 861 and 1,848 cM).

### Consensus map features

The genotypes from the core set of six RIL populations were used to build a reliable consensus framework map based on common markers only, including 295 and 281 anchor SSR and DArT markers, respectively, 21 anchor SNP/STSs and one morphological locus. These markers were grouped into 17 linkage groups (Table 2). Representative estimates of recombination rate were obtained across mapping populations, yielding a framework map of common markers with total length of 2,239 cM (Table 2), with an average inter-marker distance of 3.74 cM per common marker. The consensus framework map was thus only 15.4% longer than the longest among the six component maps (Mr × Cl), with the number of independent linkage groups decreased from 21 to 17.

**Table 2:** **Consensus framework and interpolated map marker summary**

Pop acronym	Marker and map features							
	(SSR)	DArT/AFLP (no.)	SNP/STS (no.)	Biochemical/morphological loci (no.)	Total marker (no.)	Linkage group (no.)	Total length cM	Inter-marker distance cM/marker
Consensus framework	295	281	21	1	598	17	2,239.4	3.74
Interpolated	960	1,385	225	5	2,575	17	2,463.1	0.971

Number of mapped loci (simple sequence repeat, SSR; Diversity Array Technology, DArT; amplified length polymorphisms, AFLP; single nucleotide polymorphisms, SNPs; sequence tagged sites, STSs; Mendelian loci) included in the consensus framework map (obtained from the common markers from the six core-component maps) and in the final interpolated map (with all markers), number of linkage groups, total map length (cM) and average inter-marker distance (cM/marker) for both maps.

The six core RIL maps and the consensus framework map are reported in Additional file 1: Figure S1 and Additional file 2: Table S1, with anchor markers reported in red font. In the computation of the consensus map, portions of linkage groups from single maps were not included in the merged data-set due to either excessive heterogeneity of recombination rate (*P* < 0.001) or low density of anchor markers (highlighted by dashed boxes in the figure).

Frequency of common framework markers (subdivided by marker class) and map length of each of the framework linkage groups are reported in Table 3. The 17 framework linkage groups covered most of the14 durum wheat chromosomes. Two separate linkage groups were obtained for chromosomes 1A, 2A and 3A. A relatively low number of anchor markers was observed for chromosomes 1A, 4B and 5A (23, 28 and 28 markers, respectively) while up to 78 common markers were mapped on chromosome 7B, followed by 59 common markers on chromosome 6B. The inter-marker distance between common markers ranged from 1.30 cM/marker for linkage group 3A1 and 6.95 cM/marker for 5B. The two-point mapping LOD score of framework markers averaged 47.8 across all chromosomes and ranged from 25.5 for 1A to 62.1 for 4A (Table 3).

**Table 3:** **Consensus framework and interpolated map features summarized by linkage group**

LG	Consensus framework map							Consensus interpolated map		
	Common SSR (no.)	Common DArT (no.)	Common others (no.)	Total (no.)	LOD	LG Length (cM)	Inter-marker distance (cM/marker)	All loci (no.)	LG length (cM)	Inter-marker distance (cM/marker)
1A1	5	5	0	10	25.5 ± 6.1	47.1	5.85	61	65.4	1.09
1A2	12	1	0	13	41.9 ± 9.4	54.7	4.95	32	57.0	1.84
1B1	24	17	1	40	47.7 ± 5.1	154.8	3.97	219	164.3	0.76
2A1	17	9	2	28	52.1 ± 6.7	77.1	2.86	87	104.9	1.22
2A2	6	10	1	17	57.1 ± 7.8	24.9	1.56	57	51.4	0.94
2B	20	33	5	58	47.1 ± 3.7	225.7	3.96	232	233.9	1.01
3A1	2	4	0	6	42.2 ± 12.8	6.5	1.30	33	11.6	0.36
3A2	19	16	2	37	43.2 ± 5.9	150.9	4.19	103	156.2	1.53
3B	26	18	1	45	28.9 ± 4.3	217.2	4.94	281	218.8	0.78
4A	13	35	0	48	62.1 ± 5.6	140.0	2.98	186	143.0	0.77
4B	19	5	4	28	43.2 ± 7.4	73.7	2.73	120	94.9	0.78
5A	24	3	1	28	38.7 ± 4.0	161.5	5.98	106	168.9	1.61
5B	18	11	0	29	35.1 ± 4.9	194.7	6.95	122	197.2	1.63
6A	21	8	1	30	49.4 ± 6.6	137.1	3.52	178	145.0	0.82
6B	22	37	0	59	53.5 ± 3.7	158.0	2.70	268	170.4	0.64
7A	16	25	1	42	60.1 ± 7.0	215.5	5.26	178	223.0	1.26
7B	31	44	3	78	50.8 ± 4.1	200.0	2.60	242	207.1	0.86

Number of mapped loci (simple sequence repeat, SSR, Diversity Array Technology, DArT, amplified length polymorphisms, and other markers) included in the consensus framework and in the final interpolated map, detailed by linkage group (LG).

The subsequent interpolation of the unique loci allowed us to map 2,575 loci in total, for a total map length of 2,463 cM. This final consensus interpolated map was 27.0% longer (also in terms of genome coverage) than the longest component maps Mr × Cl.

The category and number of markers mapped in the interpolated map are summarized in Table 2 and detailed in Table 3. Depending on the chromosome/linkage group, the interpolated map showed average inter-marker distances ranging from less than 1 cM/marker, e.g. 0.36-0.94 cM/marker for linkage groups 1B, 2A2, 3A1, 3B, 4A, 4B, 6A, 6B and 7B, to 1.61-1.84 cM/marker for linkage groups 1A2, 5A, 5B (Table 3). The number of markers mapped to the A genome (1,021) was considerably less than those mapped to the B genome (1,484) while the cumulative genetic distances of the linkage groups of the two genomes were balanced (1,126 cM and 1,287 for the A and B genomes, respectively).

The degree of monotony of consensus marker order as compared to the original component maps (colinearity between the interpolated map and the single core component maps) was checked by projection plots reported in Additional file 3: Figure S2. Few inconsistencies were observed for closely linked markers mapped on genetic intervals of less than 2 cM.

For most of the detected order conflicts, the original data of the component populations were re-inspected and, in most cases, it was found that the consensus marker order showed an overall LOD score and probability close to the one present in the single discordant maps; therefore, the consensus marker order was retained. Pair-wise Spearman rank correlations rho (*ρ*) of marker order between the consensus and the single component maps were generally very high across chromosomes. Out of 84 pair-wise comparisons, 76 showed *ρ* values higher than 0.99, with 50 comprised between 0.999 and 1. When considering the regression *r*^2^ values, which highlight not only departure from colinearity but also local divergence in recombination frequency, 51 out of 84 pair-wise comparisons showed values ≥ 0.99, 25 were comprised between 0.95 and 0.99 and 8 were < 0.95. Overall the *r*^2^ values were thus slightly lower as compared to rank correlation values. Local deviations from colinearity were observed only for a few chromosomes and populations as follows: in chromosomes 1A (Kf × Sv and Sm × Lv), 2A (Sm × Lv), 3A (Lg × G18-16) and 4A (Kf × Sv).

The projection plots clearly showed that (i) marker density of the single durum wheat maps differed markedly along the linkage groups and (ii) the interpolated map effectively integrated the marker information from the component maps with improved marker density and genome coverage along the consensus chromosomes, with only a slight increase in linkage group total length. The single component maps showed a high frequency of locally low marker density or even no marker coverage at all (gaps in the maps), particularly those derived from the elite × elite *T. durum* crosses. These regions were irregularly scattered across maps on most of the chromosomes (mosaic pattern). Examples can be observed in the projection plots of chromosomes 1B (Kf × Sv, Mr × Cl, Sm × Lv and Ln × G18-16 maps), 2B (Kf × Sv, Co × Ll, Mr × Cl and Sm × Lv maps), 3B (Kf × Sv, Co × Ll, Mr × Cl and Sm × Lv maps), 5B (Kf × Sv, Co × Ll, Mr × Cl, and Sm × Lv maps), 6A (Mr × Cl and Sm × Lv maps), 7A (Kf × Sv and Cl × Ll maps) and 7B (Kf × Sv map). The linkage map obtained from *T. durum* Langdon × *T. dicoccoides* G18-16 was much less affected by uneven marker density distribution as compared to the intra-specific *T. durum* maps.

In some cases, chromosome regions showing low marker density were observed consistently across the majority of the component maps and were thus considered as a constitutive feature. Examples were the centromeric portion of chromosome 1A, the proximal region of 2A, and the distal regions of 3A and 7A. As a result, these four chromosome regions also showed a lower-than-average marker density in the consensus map.

The Durum interpolated map was compared with the reference SSR consensus map of hexaploid wheat (Ta-SSR-2004 [16]; A and B wheat genomes only) (Table 4 and Additional file 4: Figure S3A). The comparison was based on the SSR markers shared between the two maps, which varied from 19 to 43 depending on the linkage group. Spearman rank correlations rho (*ρ*) values were lower than those observed within the intra-specific maps of durum wheat; the average (*ρ*) value was 0.955, ranging from 0.93-0.96 (chromosomes 1A, 1B, 6A and 6B) to 0.98 (chromosomes 2A, 2B, 5B and 7A). Coefficient of determination *r*^2^ values averaged 0.93 and ranged from less than 0.90 (chromosomes 1A, 3Aand 6A) up to 0.98-0.99 (chromosomes 1B, 2A, 3B, 5B and 7B). As compared to the Ta-SSR-2004 map, the durum-specific map herein reported provided genome coverage of 100% for the majority of the chromosomes, with the exception of chromosomes 1B and 5B were genome coverage of the durum consensus was 12.8 and 18.0% less than Ta-SSR-2004 due to low coverage of common markers on the distal ends of short arms. In chromosome 3A and 6B the durum consensus extended the genome coverage in the distal chromosome regions as compared to the Ta-SSR-2004.

**Table 4:** **Comparison between the durum consensus interpolated map and the hexaploid consensus maps**

	Durum consensus interpolated map				
	Common markers			Collinearity	
	Framework	Non framework	Total	Spearman rank correlation	Regression
Chromosomes	(no.)	(no.)	(no.)	( ***ρ*** )	***(R*** ^2^ )
**Ta-SSR-2004** ^**(1)**^					
1A	11	11	22	0.956	0.896
1B	16	14	30	0.930	0.950
2A	13	27	28	0.979	0.950
2B	15	27	42	0.983	0.935
3A	11	15	26	0.921	0.889
3B	15	26	41	0.967	0.975
4A	6	16	22	0.939	0.950
4B	12	13	25	0.963	0.900
5A	17	26	43	0.956	0.938
5B	12	17	29	0.982	0.977
6A	12	7	19	0.931	0.880
6B	12	20	32	0.940	0.921
7A	10	20	30	0.979	0.934
7B	21	14	35	0.957	0.981
Mean	21	18	30	0.956	0.934
**Chr. 3B consensus map** ^**(2)**^					
3B	21	45	66	0.981	0.961

^(1)^ Ta-SSR-2004 reported in Somers et al. (2004); [16].

^(2)^ Chr. 3B consensus map reported in Paux et al. 2008; [37].

Noticeable differences in local recombination rate were observed between the hexaploid and the durum maps. For instance, the strong decline in marker density found in the durum peri-centromeric regions of chromosomes 1A and 2A was not observed in the corresponding hexaploid regions.

As for chromosome 1A, the high recombination rate (close to independence) observed in durum wheat between the peri-centromeric regions of the two distal linkage groups (tagged by anchor markers *gwm164* and *cfa2129*) was not observed in Ta-SSR-2004, where the map distance between *gwm164* and *cfa2129* was only 6.3 cM and the marker density in the local centromeric region was not affected. Similarly, in chromosome 3A, the low-marker density, highly recombinogenic region detected between anchor markers *gwm71* (upper-side of LG) and *gwm294* (lower-side of LG) spanned only 22.8 cM in the hexaploid wheat consensus map. The updated consensus map for chromosome 3B published by Paux et al. [37] and derived from the analysis of 13 mapping populations was also considered. Sixty-six markers (mainly SSRs) were common to both the hexaploid and tetraploid 3B maps. The correlation coefficient between our map and the Paux et al. [37] map was high (*r* = 0.980).

The durum interpolated map was then compared with the most recent tetraploid consensus map [19] obtained with different software (JoinMap). In this case, while the number of mapped markers was only slightly lower than in our case (1,898 and 2,575 markers, respectively), the results were different in terms of number of LG obtained (27 *vs*. 17, respectively), and total map length (3,058 *vs*. 2,575 cM, respectively). Overall conservation of colinearity of the two durum consensus maps and Ta-SSR-2004 was better for our map (Additional file 4: Figure S3A) than for the Marone et al. [19] map (Additional file 5: Figure S3B).

### LD decay rate and molecular diversity in elite durum wheat germplasm

**Figure 1:**
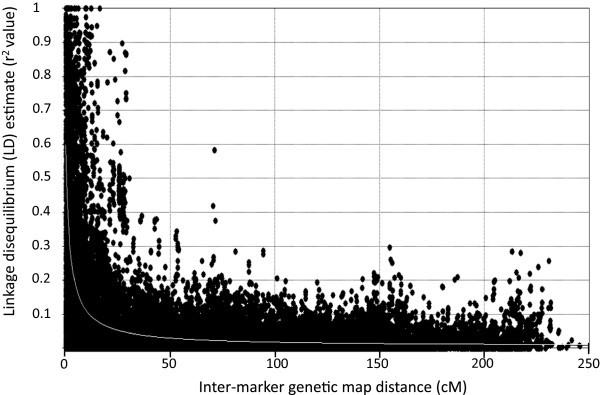
**Intra-chromosomal linkage disequilibrium (LD) decay plot as a function of genetic distance (cM).** LD decay assessed in a durum wheat panel of 183 elite (cultivars and advanced lines) accessions adapted to the Mediterranean environments. LD estimates are reported as squared correlations of allele frequencies (*r* ^2^). Inter-marker genetic distances are from the durum wheat consensus map. Non-linear modeling of LD was performed using the Sved [39] equation.

**Figure 2:**
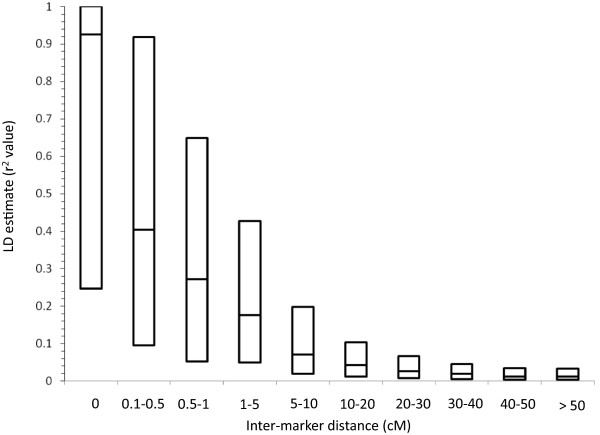
**Boxplot charts of linkage disequilibrium (LD) estimates for categorized inter-marker genetic map distances (cM).** Pairwise LD estimates are reported as squared correlations of allele frequencies. Inter-marker genetic distances are from the durum wheat consensus map.

A panel of 183 elite durum accessions representative of the major gene pool cultivated in the Mediterranean region for which LD and population structure were previously investigated based on low-density genotyping with SSR markers (DurumPanel, [38]) was re-investigated in depth based on the profiles of 1,200 markers included in the consensus map. Of these 1,200 markers, 957 showed minor allele frequency (MAF) equal or higher than 0.10 and were thus considered informative for LD and AM analysis. This set of markers, including 334 SSR/STSs and 623 DArT, generated 35,145 pairwise combinations of intra-chromosomal loci for which LD significance and *r*^2^/*D*’ estimates were calculated. LD decay was investigated through fitted regression and box plot distribution, (Figures 1 and 2, respectively), with a focus on the short inter-marker distances (Figure 2 and Table 5). Non-linear modeling of LD decay reported in Figure 1 showed an overall *R*^2^ fit value of 42%, an α value of 1.69 and an effective population size (Ne) of 19.36. Based on the fitted model, at 0 cM genetic distance the expected *r*^2^ value was equal to 0.6 while the *r*^2^ half-value decline (*r*^2^ = 0.3) was reached at 2.20 cM. The upper 95^th^ percentile of the *r*^2^value distribution for unlinked marker pairs (inter-marker distance ≥ 50 cM), widely considered as a threshold useful to separate the true linkage LD from the background LD due to population structure, was equal to 0.096 (close to 0.10). This trend was confirmed by inspecting the LD statistics for discrete marker pairwise classes of incremental map distances (Table 5 and Figure 2). The class of completely linked markers (with inter-marker distance = 0 cM, including 504 pairs) showed mean *r*^2^ = 0.67, median LD *r*^2^ = 0.92, inter-quartile range from 0.25 to 1.0 and D’ = 0.86. The mean and median LD *r*^2^ decreased by half in the 0.5-1 cM inter-marker distance class (mean *r*^2^ = 0.36 and median LD *r*^2^ = 0.27) and more than half in the 1-5 cM class, respectively; the median LD *r*^2^ values dropped below 0.10 in the 5-10 cM class (mean *r*^2^ was 0.15 and median *r*^2^ = 0.07, respectively). The LD estimates calculated for independent markers (pairs of markers more than 50 cM apart; 18,139 pairs in total) showed mean *r*^2^ = 0.025 and D’ = 0.23.

**Table 5:** **Linkage disequilibrium estimates for the elite durum wheat panel based on mapped SSR and DArT markers**

Marker pairs		Linkage disequilibrium estimate	
Distance (cM)	Count (no.)	Average ***r*** ^2^	Average ***D*** ’
0	504	0.674	0.858
0 - 0.5	392	0.473	0.767
0.5 - 1.0	306	0.357	0.658
1.0 - 5.0	2,677	0.275	0.663
5.1 - 10.0	2,437	0.150	0.525
10.1 - 20.0	3,256	0.087	0.398
20.1 - 30.0	2,667	0.066	0.323
30.1 - 40.0	2,386	0.036	0.283
40.1-50.0	2,381	0.029	0.254
> 50	18,139	0.025	0.232

LD decay in a collection of 183 elite durum wheat accessions from the Mediterranean, CIMMYT and ICARDA germplasm included in the association mapping Durum Panel.

Detailed pair-wise LD *r*^2^ values for all the consensus linkage groups are projected as heat-plot triangular matrices in Additional file 6: Figure S4, together with the polymorphism index content plots along the chromosomes (reported as standardized average PIC values of a three-marker sliding window) and the distributions of markers with highly significant (*P* ≤ 0.01) genetic differentiation (*F*_ST_ indexes) among the main sub-populations represented in the panel. When detectable, LD blocks with sizeable *r*^2^ values (≥0.40) were in most cases observed within a 5 cM window size; relatively small LD blocks were detectable across the full length of durum wheat chromosomes, from distal to proximal regions. However, a large number of closely linked adjacent markers showed LD *r*^2^ values in the range of 0.3 or less.

A few long-range LD blocks extending more than 10 cM were also observed, particularly in the proximal regions of chromosomes 1B, 6A and 7B. The polymorphism information content (PIC) plots showed a trend for higher values in the distal regions of the chromosomes and lower values in the peri-centromeric regions (e.g. chromosomes 1B, 3B, 6A and 7B). Chromosome 1A exhibited the most extended peri-centromeric region with both low marker density and diversity in the elite germplasm, causing a gap in the continuity of the chr. 1A consensus map in this region. A similar phenomenon was observed for peri-centromeric regions of chromosomes 2A, 2B and in 3AS. None of the six core RIL maps showed a marker density sufficiently high to guarantee the continuity of the consensus linkage groups in these four chromosomes. Additionally, numerous locally defined sharp decreases of PIC values extending over stretches of 5 to 10 adjacent markers were commonly observed in both centromeric and distal regions of several chromosomes (e.g. in chromosomes 1B, 2B, 3B, 4A, 5B, 6B, 7A and 7B, see Additional file 6: Figure S4), even in chromosome regions where the marker density was not locally affected.

The population structure of the elite panel of accessions was previously investigated based on a non-redundant set of 96 highly informative SSR markers (Maccaferri et al. [20, 38]). Hierarchical and non-hierarchical model-based cluster analyses showed that the population structure could be described based on five main sub-populations corresponding to landraces- and breeding-derived germplasm founders that originated from the Italian, CIMMYT and ICARDA breeding programs distinct by time period (e.g. early-, mid- and late-CIMMYT derived sub-populations) or target environment (e.g. the ICARDA germplasm for dryland *vs*. temperate areas). The founders were widely used in the national breeding programs of Mediterranean countries and a high degree of admixture was detected among sub-populations [38].

The increased density of genetically mapped markers based on the consensus map allowed for a more in-depth investigation of the patterns of genetic differentiation present within and among the five main sub-populations. AMOVA showed that the genetic variation within was higher than the variation among sub-groups (82.8 *vs*. 17.2, Table 6), with the latter (summarized by the pair-wise F_ST_, Table 6) reflecting known temporal, geographic and pedigree differences. A medium level of genetic differentiation was observed between the two ICARDA sub-populations for dryland and temperate areas (pair-wise F_ST_ = 0.172, a value equal to the overall FST value). More differentiation was found between the ICARDA sub-population for dryland areas and the Italian and early-period CIMMYT germplasm (pair-wise F_ST_ = 0.261). Lower-than-average differentiation was observed between the ICARDA-temperate and the Italian-early CIMMYT as well as the mid-CIMMYT. The late-CIMMYT germplasm (*F*_ST_ = 0.361 and 0.306) was the most differentiated from the ICARDA-dryland and Italian-early CIMMYT sub-populations.

**Table 6:** **Analysis of molecular variance (AMOVA) for the elite durum wheat panel based on mapped SSR and DArT markers**

Sub-populations		Variance components				
(no.)		Within subpopulations	Among subpopulations			
5		82.8%	17.2%	FST = 0.172		
**Sub-population pairwise F** ST						
**Sub-population**	**Founder**	**(1)**	**(2)**	**(3)**	**(4)**	**(5)**
ICARDA-dryland (1)	Omrabi 5	-				
ICARDA-temperate (2)	Cham 1	0.172	-			
Italian, early CIMMYT (3)	Valnova, Mexicali 75	0.261	0.109	-		
Mid CIMMYT, ICARDA (4)	Yavaros 79	0.249	0.067	0.239	-	
Late CIMMYT (5)	Altar 84	0.361	0.163	0.306	0.156	-
**Pairwise differences (no.)**		**216.3**	325.6	351.7	323.1	337.0
		64.0	**306.9**	336.7	303.4	314.3
		97.5	37.2	**292.1**	359.6	362.2
		85.5	20.5	84.0	**259.0**	281.6
		121.5	53.5	108.8	44.8	**214.7**

Analysis of molecular variance for the elite durum wheat panel of 183 accessions adapted to Mediterranean areas based on the sub-population subdivision as from a previous population genetic structure analysis [20, 38]. Overall FST index among sub-populations and specific sub-population pairwise FST indexes are reported. Average population pairwise differences are reported as follows: (*i*) above diagonal: average number of pairwise differences between populations (P_*i*_ XY), (*ii*) diagonal elements (**bold**): Average number of pairwise differences within population (P_*i*_ X), (*iii*) below diagonal: Corrected average pairwise difference (P_*i*_ XY-(P_*i*_ X + P_*i*_ Y) /2).

The improved marker density of the consensus map allowed us to search genome-wide for chromosome regions showing evidence of high genetic differentiation among sub-populations (locus by locus AMOVA). Single markers or chromosome regions (stretches of two or more adjacent markers, i.e. blocks) with sub-population pair-wises *F*_ST_ values greater than the *F*_ST_ thresholds set at *P* 0.01 were observed in most of the chromosome arms and projected on the consensus map (Additional file 6: Figure S4). Three main genetic differentiation patterns were observed as follows: (*i*) F_ST_ differentiation “pattern 1”: significant differentiation across the majority of sub-populations indicating differentiation by time and by target environments (highly significant F_STs_ in 7 or more of the 10 possible sub-population pairwise comparisons, (*ii*) F_ST_ differentiation “pattern 2”: significant differentiation between ICARDA-dryland *vs*. all the other subpopulations (highly significant F_STs_ in 3 or more of 4 pair-wise comparisons), (*iii*) F_ST_ differentiation “pattern 3”: significant differentiation of either or both ICARDA-temperate and Italian, early-CIMMYT subpopulations *vs.* the mid- and late-CIMMYT subpopulations (highly significant F_STs_ in 3 or more out of 4 sub-population pair-wises). There were 11, 22 and 54 single markers/chromosome regions in the durum wheat genome that showed genetic differentiation pattern 1, 2 and 3, respectively (reported in Additional file 6: Figure S4). F_ST_ differentiation pattern 1 was mainly found on chromosomes 1B, 2A, 2B, 4B, 5A, 5B and 6B. High frequency of both pattern 2 and 3 were observed in chromosomes 3A and 7B. Pattern 3 was also frequently found in chromosomes 1B, 3B and 7A.

In chromosome 2A, *Ppd-A1* and four closely associated flanking markers showed highly significant F_ST_ differences for all sub-population pair-wises (F_ST_ differentiation pattern 1). In chromosome 2B, coincident with *Ppd-B1*, (wPt-7320 at 47.7 cM position and associated markers) a strong differentiation was observed between the Italian and ICARDA populations (bred in temperate zones) *vs*. the CIMMYT sub-populations bred at sub-tropical latitudes (F_ST_ differentiation pattern 3). As expected, *Rht-B1* on chromosome 4B showed a type 2 F_ST_ differentiation pattern with peaks of *F*_ST_ values for the pair-wise comparisons including the ICARDA dryland population (with high frequency of tall accessions) *vs*. all the other populations (nearly fixed for the semi-dwarfing *Rht-B1b* allele).

### GWAS for heading date

The consensus map was used for a genome-wide association scan (GWAS) to detect association of the 957 mapped SSR and DArT markers with MAF > 0.10 with time to heading in the durum wheat association panel. Time to heading data were available for 27 field trials carried out in the Mediterranean region and in Mexico. The AMMI biplot analysis showed that the heading date phenotypes from the 27 environments could be grouped according to five macro-environments: Southern Europe (North to Southern Italy and Spain), North Africa-Tunisia, North Africa-Morocco, West-Asia (Syria and Lebanon) and Mexico (data not shown). The association scan was performed for each environment as well as for the adjusted means of each of the five macro-environments.

Broad-sense heritability (*h*^2^) values of heading date calculated across environments were high for Southern European (0.85), Asian (0.89) and Mexican macro-areas (0.91), including 10, 8 and 5 environments, respectively, but lower for the North African Moroccan (0.69) and Tunisian (0.68) macro-environments, each with two environments. The phenotypic data of six genotypes known to carry the vernalization-sensitive *vrn-1* allele at *VRN-A1* were excluded from the analysis.

The initial GWAS analysis showed strong experiment-wise significant associations at the two chromosome regions corresponding to the location of *Ppd-A1* and *Ppd-B1* loci and numerous chromosome regions with highly significant (*P* ≤ 0.01) marker-wise associations across environments. Detailed results are reported in Table 7.

**Table 7:** **Association results for heading date in the elite Durum Panel at the two major photoperiod-response homeologous** ***Ppd-1*** **loci**

	Causative locus: ***Ppd-A1***						Causative locus: ***Ppd-B1***					
	Single envs.				Adj. means across environments		Single envs.				Adj. means across environments	
**Macro-environmental area**	*P* 1E-4	*P* 0.001	*P* 0.01	*P* 0.05	*P*-value	*R* ^2^	*P* 1E-4	*P* 0.001	*P* 0.01	*P* 0.05	*P*-value	*R* ^2^
	(no.)	(no.)	(no.)	(no.)		(%)	(no.)	(no.)	(no.)	(no.)		(%)
**Southern Europe (10 envs.)**												
Across envs.					3.76E-08	18.76					8.68E-04	6.46
Single env. (range)	4	2	1	3	6.02E-12	(3.74 - 31.05)	1	2	1	5	1.56E-06	(4.64 - 12.71)
**North Africa – Tunisia (2 envs.)**												
Across envs.					2.65E-04	8.58					0.016	3.60
Single env. (range)	0	2	0	0	1.95E-05	(6.78 - 8.91)	0	0	1	1	0.010	(1.80 - 4.75)
**West Asia (8 envs.)** **(8 envs.)**												
Across envs.					4.76E-08	17.90					0.003	4.98
Single env. (range)	5	3	0	0	2.40E-06	(7.99 - 14.42)	0	0	6	1	0.001	(4.10 - 6.61)
**North Africa – Morocco (2 envs.)**												
Across envs.					1.16E-10	31.17					0.008	4.74
Single env. (range)	2	0	0	0	6.30E-10	(26.53 - 28.30)	0	0	1	1	0.009	(4.50 - 4.55)
**Mexico (5 envs.)**												
Across envs.					4.60E-13	34.31					0.007	4.19
Single env. (range)	5	0	0	0	1.69E-14	(24.66 - 39.98)	0	0	2	0	0.001	(4.61 - 5.97)

Association analysis based on 183 accessions from the durum panel, evaluated for heading date across 27 environments grouped in five macro-environmental areas in the Mediterranean Basin and Mexico. The *Ppd-A1* specific assay [40] and wpt-7320 DArT marker tightly associated to *Ppd-B1* have been used. Significance of *P* ≤1.00E-4 refers to the experiment-wise significance threshold of *P* ≤ 0.05. Results are reported for single environments and for the adjusted means across macro-environmental areas.

At *Ppd-A1*, association peaks were detected in coincidence of the diagnostic marker developed from the causal locus. Considering the adjusted means of macro-environmental areas, *P*-values of association ranged from a minimum of *P* = 3.76E-08 (*R*^2^ value = 18.8%) for the Southern European area (that grouped the environments at higher latitudes among those tested), to a maximum significance of *P* = 4.60E-14 (*R*^2^ value = 34.3%) in Mexico (with environments located at lower latitudes). Among the four markers *wmc177*, *cfa2201*-2A, *gwm1198*-2A and wPt-7026 located within 4.5 cM from the *Ppd-A1* locus and with LD *r*^2^ values between 0.2 and 0.4, significant associations with heading date were observed for *cfa2201*-2A only (*P* ≤ 0.05, at 2.6 cM from *Ppd-A1*). For the region harboring *Ppd-B1*, highly significant marker- and experiment-wise associations with heading date were detected for 10 DArT and one SSR markers mapped within an interval of 8.3 cM (from wPt-5513 at position 45.2 to wPt-6199 at position 53.5 on chromosome 2B). The majority of these markers formed a unique linkage block with strong inter-marker LD *r*^2^ values (from 0.5 to 1.0, except for *gwm1198*-2B). Peaks of association to heading date across environments were observed for wPt-7320, the QTL-representative marker, at position 47.7 cM. This marker showed a range of *P*-values from a minimum of *P* = 0.007 (*R*^2^ value = 4.19%) in Mexico to a maximum association of *P* = 0.868E-04 (*R*^2^ value = 6.46%) in Southern Europe. According to Hanocq et al. [41–43] and Bennett et al. [44], the small linkage block includes markers associated with the predicted position of *Ppd-B1*.

**Figure 3:**
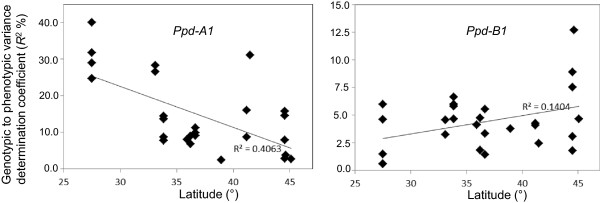
**Scatterplot of the relationship between latitude and genotypic to phenotypic variance coefficient of determination (R** ***2*** **) values for** ***Ppd-A1*** **and** ***Ppd-B1*** **in 27 Mediterranean and Mexican environments.**

As expected, the level of significance and magnitude of effects associated with the *Ppd* loci were strongly influenced by latitude. The genotypic to phenotypic proportion of variance of *Ppd*-QTLs, as estimated by *R*^2^ values on a single-environment basis, was significantly correlated with latitude (correlation coefficient *r* = -0.60 for *Ppd-A1* and *r* = 0.35 for *Ppd-B1*) as presented in Figure 3. *Ppd-A1* showed a strong trend towards increased *R*^2^ values at latitudes lower than 40° (Southern Italy, Syria and Lebanon, Tunisia, Morocco and Mexico) while the markers tagging *Ppd*-*B1* showed an opposite trend.

**Figure 4:**
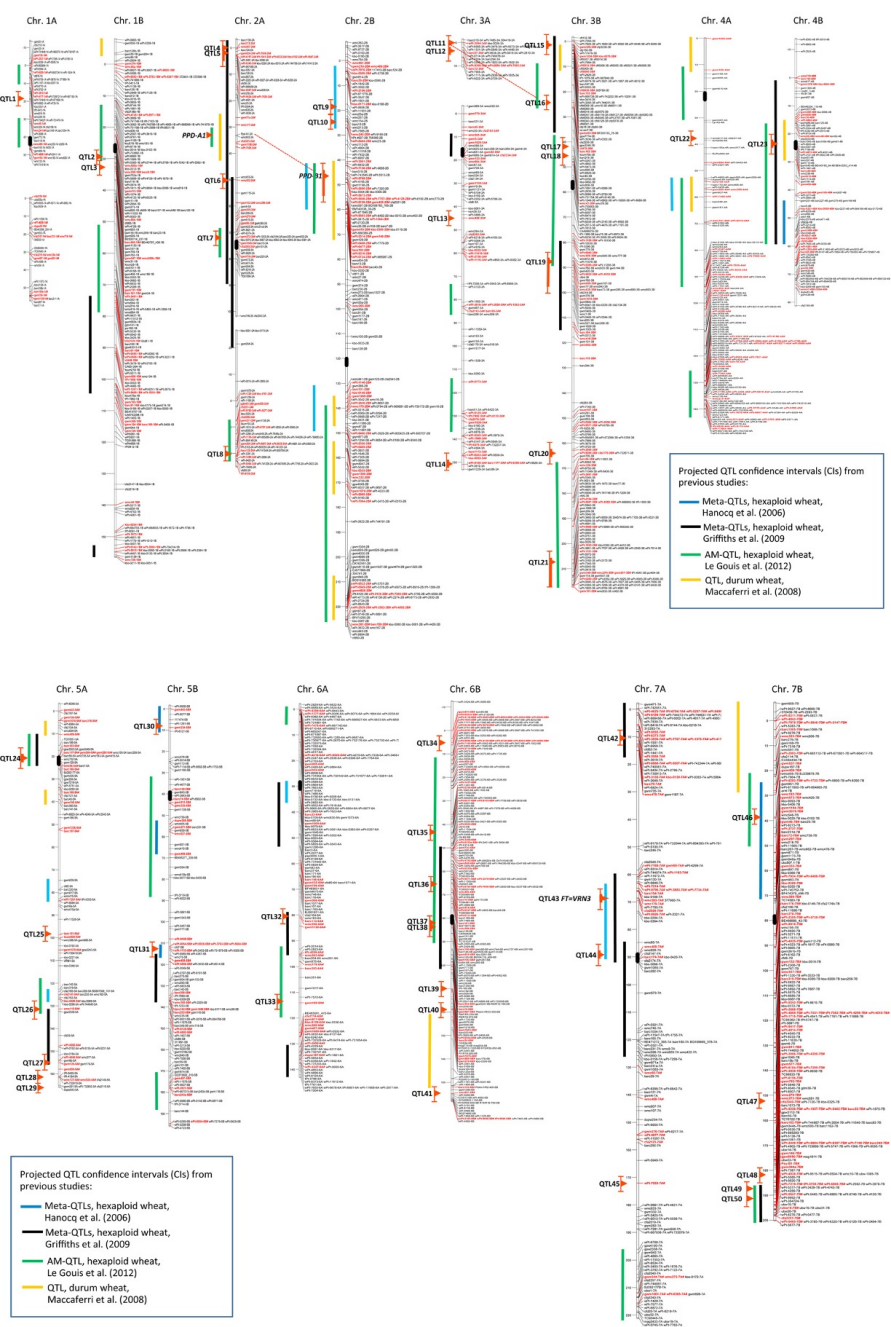
**Association mapping QTLs (AM-QTLs) for time to heading in the Mediterranean regions reported on the tetraploid SSR and DArT** ^**®**^ **marker consensus map.** AM-QTLs are reported as red bars (significance intervals) on the left side of the chromosomes. Red thin wiskers extending the red bars corresponds to the confidence intervals (CIs) of AM-QTLs. Projected QTL confidence intervals from previous studies are reported as vertical bars of various colors other than red.

The GWAS was repeated after including *Ppd-A1* and *Ppd-B1* representative markers as covariates in the MLM association model. Based on this analysis, 50 unique chromosome regions showed highly significant (*P* ≤ 0.01) association to the heading date adjusted means of at least one or more of the five macro-environmental areas. Marker-features of these regions are summarized in Additional file 7: Table S2 and Figure 4. None of these QTL-harboring regions reached the experiment-wise adjusted Bonferroni significance threshold based on the number of independent LD blocks (*P* ≤ 0.0001). However, the reported associations were all supported by marker-wise significances (*P* ≤ 0.05) over multiple environments (9.2 ± 4.1 on average, out of 27 tested environments), as detailed in Additional file 7: Table S2. Out of the 50 AM-QTLs, 29 consisted of single-marker associations (singletons) while 21 consisted of small blocks of multiple adjacent markers all associated to the phenotype as well as to each other with LD *r*^2^ values ≥ 0.3. From the consensus map, these QTLs included markers that were either coincident (i.e. at 0 cM distance) or closely linked within a 3-4 cM interval. There were only three QTLs with a significance interval of 5 cM or more on chromosomes 2B (QTL-10, tagged by *gwm1027*, interval = 5.3 cM) and 3B (QTL-19, tagged by wPt-9510, interval = 10.8 cM and QTL-21, tagged by wPt-9989, interval = 8.5 cM). Based on the predicted LD decay in the collection, confidence interval boundaries of 2.2 cM, corresponding to the genetic distance over which the LD decays to *r*^2^ = 0.3, were attributed to both sides of the marker’s blocks significantly associated with the phenotype. Thus, a confidence interval of 4.4 cM was attributed to the single markers associated with the phenotype. The *R*^2^ values of the associations on a macro-environmental basis were mostly between 1.5 and 4.5% (Additional file 7: Table S2). The proportion of phenotypic variance explained by simultaneously considering all the reported QTL-representative markers (including the two *Ppd-1* homeologs) ranged from a minimum of 59.6% for the Tunisian macro-area to a maximum of 79.6% for Mexico, with *R*^2^ values of 64.6, 67.3 and 68.9% for the Southern-European, Asian and Moroccan macro-areas, respectively.

AM-QTLs were distributed in both distal and pericentromeric regions of the chromosomes. Out of 50 AM-QTLs, 16 were identified in regions within 20 cM from the centromeres while 21 were positioned in the very distal telomeric chromosome regions. Thirty-two AM-QTLs were positioned within the CIs of the hexaploid wheat meta-QTLs or the Kofa × Svevo QTLs, although in many cases the CIs of hexaploid wheat meta-QTLs were excessively wide (intervals ≥ 20 cM) to support QTL coincidence. The map location of the *Ppd-1* homeologs and 50 AM-QTLs (representative marker position and CIs) on the consensus map is reported on Figure 4, along with the projections of the CI of the meta-QTLs and AM-QTLs found in hexaploid wheat [41, 42, 45] and the QTLs from the durum wheat RIL population Kofa × Svevo [24]. Co-locations between AM-QTLs and hexaploid wheat meta-QTLs with CIs shorter than 20 cM or Kf × Sv QTLs were observed for 16 AM-QTLs (QTL7, 9, 10, 15, 16, 23, 24, 26, 30, 31, 32, 42, 43, 46, 49 and 50; see Figure 4). Apart from *Ppd-A1* and *Ppd-B1*, homoelogous effects between A- and B-genome QTLs could be hypothesized for QTL11 and QTL16 as shown by significant effects at both of the homeologous copies tagged by the SSR marker *cfd79*. QTL43 (tagged by *cfa2028* and wPt-7785) has been positioned very close (within 3 cM) to the location of *TaFT1* in chromosome 7A of hexaploid wheat reported by Bonnin et al. [46]. The homeolog of *TaFT1* in the B-genome, mapped by Yan et al. [47] at 5 cM away from *gwm569* in the distal portion of 7BS, was not identified with this survey, although in this region a major QTL for heading date was reported in the Kofa × Svevo mapping population (QTL projections reported in Figure 4).

## Discussion

### Integration of SSR and DArT in a common framework

The consensus framework and the final interpolated map reported here integrates a large portion of the mapping efforts carried out in durum wheat. Our integrated data of 14 maps is based on a combination of segregation- and interpolation (‘projection’)-based methods.

Efficient projection-based integrations requires a pre-existing reference (fixed) framework map with a large number of high-quality markers and nearly complete genome coverage that serves as a backbone for marker projection from the component maps. For durum wheat, this resource was not available. The reference maps initially assembled with RFLP and SSR markers [6, 11] were limited either in the number of progenies or markers used. Here, a core of six maps with adequate number of progenies, medium-to-high number of SSR and DArT mapped loci, homogeneous genetic constitution (recombinant inbred lines) and complete genotype datasets available were identified as the best resource for assembling a robust framework. All but one of these maps had parents derived from the cultivated durum wheat germplasm. Based on these commonalities, this segregation data-set was considered as the most suitable to produce a consensus framework based on actual genetic map distances calculated from a data-set of lines with relatively homogeneous constitutions that well represents the durum germplasm.

As compared to the projection-based method, building *de novo* consensus maps from a pooled set of segregation data was recently criticized [48, 49]. However, in our case it proved to be the best solution for obtaining a reference map with nearly complete genome coverage because none of the elite × elite durum wheat maps showed complete genome coverage, likely due to the scattered occurrence of identity by descent (IBD) in the genomes of parental genotypes. The detection of shared long-range haplotypes in components of cultivated durum wheat germplasm was previously reported based on multi-allelic mapped SSR markers [30, 50]. On the other hand, the presence of locally strong differences in recombination rate among populations, which can affect the precision of marker ordering in *de novo* consensus maps from pooled segregation data, was monitored and was not considered as a main perturbing factor as could be the case when using populations derived from crosses between more distantly-related materials [51]. The quality of the consensus framework map was checked based on the overall high two-point linkage LOD values for common markers throughout all chromosomes. Interpolation was then effectively used to project all the markers that were uniquely mapped in each of the 14 maps available.

Despite the integration of six core maps, gaps in the consensus framework map were observed for four regions on chromosomes 1A, 2A, 3A and 7A. At least for chromosomes 1A and 2A, the presence of gaps of marker coverage was reported in the consensus map of Marone et al. [19] and was found to correspond to the centromeric deletion-bins of wheat. The biological reasons at the basis of this molecular evidence deserves further work based on comparative genomics diversity studies among *T. aestivum*, *T. durum*, *T. dicoccum* and particularly the wild emmer *T. dicoccoides*. This would allow one to test the hypothesis of occurrence of local severe speciation and domestication-related bottlenecks/selective sweeps [52], or, alternatively, the presence of strong local differences in genomic constitution and/or alterations of the genetic to physical distance ratio among the *Triticum* species (in particular, hexaploid *vs*. tetraploid wheat).

The robustness of the marker order obtained in our final interpolated map is demonstrated by the overall high rank correlations (*ρ*) between the marker order of linkage groups from single component maps and the marker order of the consensus linkage groups. Correlation coefficients this high have rarely been observed in similar studies [48]. Comparison with the Ta-SSR-2004 map of Somers et al. [16] in hexaploid wheat revealed good but less conserved overall collinearity and low conservation of relative genetic distances. This could in part be due to the different algorithms used to produce the consensus maps. Joinmap software calculates consensus map by a regression algorithm only, while Carthagene allows one to work with the maximum likelihood even for integrated datasets, coupled with effective marker order refining procedures. This could also account for the lower-than-expected performance of the durum consensus map reported here compared with that of Marone et al. [19], as well as more obvious differences such as the raw data quality assessment and mapping procedures.

### Application of the consensus map for linkage disequilibrium and germplasm diversity analysis

The final interpolated map was used to investigate at relatively high level of genetic resolution important genomics and breeding applications in *T. durum* such as: (i) describing the LD pattern and LD decay rate in elite germplasm, (ii) exploring the pattern of genetic diversity among sub-populations of germplasm at the chromosome level, and (iii) performing GWAS. To these ends, the durum wheat elite accession panel previously assembled for AM studies was used as reference. The pattern of LD present in the panel was re-estimated after Maccaferri et al. [38], where only low-density SSR markers were used. The higher SSR and DArT marker density in the present map provided a more detailed and accurate picture of LD decay in the crucial 0 - 5 cM interval. In particular, LD in the elite Mediterranean panel decays below the *r*^2^ reference threshold of 0.3 within 2.20 cM. This *r*^2^ threshold is considered as a reference as regards to the power of GWAS to detect association to QTLs with medium to high effect [53]. Interestingly, the inferred average LD *r*^2^ value is 0.6 at 0 cM inter-marker distance, i.e. at the maximum resolution provided by the linkage studies, is far below the reference threshold of *r*^2^ = 0.8 currently accepted as the *r*^2^ value to select representative SNPs for GWAS (tagSNPs, [54]) in those species where this resource is available. These data indicate that actually the genetic resolution of AM in this elite germplasm might be higher than expected based on previous assumptions and that, based on these results and on the length of the durum consensus map, exhaustive GWAS in elite *T. durum* germplasm will require a marker density in the order of tens of thousands markers. The reported results indicate that AM had a mapping resolution of approximately 10 times higher in magnitude as compared to that obtained with conventional linkage mapping, even in the elite germplasm of an autogamous species such as durum wheat. The LD decay pattern of this elite Mediterranean germplasm panel compares favorably with that of populations with a more restricted genetic basis of elite durums from other areas. Somers et al. [55] for a global panel with North American, European, CIMMYT and Argentinian germplasm reported similar genetic distances of 2-3 cM for the LD decay in such germplasm [27, 55]. As a comparison, LD decay to 50% in US elite hexaploid winter and spring wheat was observed at larger genetic intervals (5 to 7 cM, respectively, [56]). Another interesting feature of the LD decay pattern observed along the chromosome is that, with only a few exceptions, the genetic resolution did not markedly decline in the peri-centromeric regions.

The interpolated map proved useful also for a first in-depth investigation of the distribution of the chromosome regions characterized by peaks of differentiation (high F_ST_ values) among the durum sub-populations represented in the AM panel. Evidence of differentiation was found for chromosome regions identified by three or more adjacent markers (LD blocks), and not only at the single-marker level. The approach performed effectively, because high differentiation was detected near the major *RhtB-1* and *Ppd-A1* loci, as expected. In hexaploid wheat, this approach was used based on SNPs at medium mapping density [56, 57]. With the durum panel, the three patterns of genetic differentiation among sub-populations that were most frequently found along the chromosomes might correspond to regions harboring genes governing the morpho-physiological, adaptive and qualitative traits that were differentiated among sub-populations, as demonstrated for the *PPD* homeologs. Interestingly, a relatively high number of chromosome regions were found to differentiate the more recently developed CIMMYT elite durum breeding lineages from the others (chromosome regions with F_ST_ differentiation pattern 3), suggesting that the breeding innovation process of adding new valuable genetic diversity to the existing ones operated successfully.

### Application of the consensus interpolated map for mapping QTLs controlling heading date

The evidence for an average LD decay in the cultivated durum wheat germplasm within less than 5 cM prompted us to assess the potential of GWAS analysis for an important adaptive trait such as heading date and to use the consensus map as a framework for positioning AM-QTLs.

The mapping resolution of AM-QTLs reported here (QTLs mapped with an average confidence interval of 4.4 cM) appeared to be similar or higher to that observed for the same trait in the cultivated germplasm of hexaploid wheat (10 cM confidence interval) investigated by Le Gouis et al. [45]. Mapping resolution estimated at the whole-genome level using the average LD decay rate was confirmed by the association results obtained for the markers closely mapped to the major effector loci *Ppd-A1* and *Ppd-B1*. The marker density used in the present study (861 markers with MAF ≥ 0.10) appeared adequate for medium-density genome-wide scans, as shown by the medium to high overall *R*^2^ values obtained by fitting the multiple-QTL model with all the highly significant QTL effects [58].

The potential of GWAS as an effective tool for QTL discovery, when coupled with sufficient marker density and reliable genetic map information, was evident from the high number of QTLs (up to 50) that were mapped consistently across environments. Heading date in the spring elite durum wheat germplasm was confirmed to be controlled by a few major loci (namely, the *Ppd-1* homeologs) as well as by a wide range of small-effect QTLs with *R*^2^ values below 5%, according to the ‘L-shaped’ distribution of QTL effects previously observed in most crop germplasm QTL analyses [58–62].

Although the number of mapped markers used here for GWAS (957) is among the highest reported for similar studies in wheat, it is also evident that reaching higher mapping precision and complete genome coverage (inter-marker LD *r*^2^ values = 0.8, [63]) will require a marker density at least ten times higher, even in cultivated germplasm. Currently the marker technology in wheat is changing to single nucleotide polymorphisms (SNPs), which hold great promise for high-throughput multiplex assays [18, 57, 64].

Until now, the SSR markers generally deployed in wheat genomics were mainly obtained from genomic libraries, and only a limited number of DArT markers (up to 2,000) have been sequenced in wheat (http://www.diversityarrays.com/DArT-map-sequences). Therefore, comparative mapping across species with these two marker classes has limited resolution in wheat. This notwithstanding, attempts to use new marker technologies such as SNPs have already been carried out [26]. The use of transcript-derived SNPs will greatly facilitate the investigation of the putative gene content in the QTL-harboring regions, eventually leading to a more rapid identification of candidate genes at least for those cases where direct links between the putative gene function and the phenotype can be established.

As shown from the comparison between the CIs of AM-QTLs and the projections of the CIs from previous meta-QTL analysis carried out in hexaploid wheat, it was evident that the meta-QTL CIs from bi-parental RIL studies were substantially wider than those of the AM-QTLs, in the magnitude of 10 (minimum) to 30 cM (maximum) thus making it difficult to accurately infer QTL co-locations. It is expected that in the near future the increasing number of QTL mapping results will allow for higher mapping resolution of meta-QTL analysis [65]. Additionally, the adoption of common high-density SNP genotyping assays will also greatly facilitate the comparison of QTL regions across studies, germplasm and laboratories.

From the comparison between the mapping locations of AM-QTLs and the QTLs for heading date found in the intra-specific Kofa × Svevo durum mapping population, it was also evident that the bi-parental population permitted mapping of several (up to 9) QTLs that were not identified in the AM study. This result underlines the concept that the compilation of the QTLs present in the cultivated germplasm is far from being complete by relying on a single AM study. In particular, linkage analysis has the advantage over AM of being more efficient in detecting alleles that are present in rare frequencies in the target germplasm, as could be the case for the QTLs detected in the Kofa × Svevo durum mapping population, and alleles whose distribution correlates to the genetic population structure. On the contrary, AM studies carried out with the necessary marker density and population size show a favorable trade-off between the phenotyping cost and the output in terms of QTL identification.

## Conclusions

Overall, results from this study provide an exhaustive consensus framework map of SSR and DArT markers for the durum wheat genome that was used to assess the genetic structure of cultivated durum wheat germplasm at the level of single chromosome regions and to assess the potential of GWAS to dissect the genetic basis of heading date. The reported results showed that the marker density provided by the consensus map was sufficient to identify chromosome regions harboring numerous small effect QTLs for heading date, at a resolution level not possible with traditional bi-parental mapping studies. More importantly, the results showed that there is scope for further refinement and expansion of these results as high-density and high-throughput genotyping SNP platforms become available. In view of this impending change in the commonly-applied genotyping technology, framework mapping data-sets that fit commonly accepted quality thresholds are valuable to provide a link between the QTL mapping results obtained in the last decade by means of SSR and DArT technology and the up incoming SNP-based technology.

## Methods

### Core maps used for assembling the framework consensus map

Six maps from core recombinant inbred line (RIL) mapping populations were chosen for building a reference consensus maps using common (anchor) loci. These maps were chosen for marker density, presence of both SSR and DArT^®^ markers and genome coverage (based on the map projections on the Ta-SSR-2004 consensus map used as a reference). Five populations were derived from *T. durum* intra-specific elite crosses and one population was derived from the inter-specific cross *T. durum* × *T. dicoccoides*. Segregation data were available for all the recombinant inbred lines.

Four mapping populations were developed and genotyped jointly with Produttori Sementi Bologna (PSB), University of Bologna, and University of Udine, Italy. The Kofa × Svevo population (Kf × Sv) included 249 RILs and was profiled with SSR markers [24, 66]. The Colosseo × Lloyd (Co × Ll), Meridiano × Claudio (Mr × Cl) and Simeto × Levante (Sm × Lv) populations included 176, 181 and 180 RILs, respectively, and were profiled with SSR and DArT markers from the Durum-specific *Pst*I/*Taq*I representation v. 2.0 Array (Triticarte, Yarralumla, AU). The Mr × Cl linkage map was subsequently enriched with DArT markers from the wheat high-density array v. 3.0. Further details are reported in Mantovani et al. [13] and Maccaferri et al. [12, 24, 38, 67]. The fifth population was developed at UC Davis from the cross Kofa × UC1113 (Kf × UC), it included 93 lines and was profiled with SSR and SNP markers [68]. The sixth mapping population was developed at University of Haifa, Israel, from *T. durum* Langdon (Ln) × G18-16 (*T. dicoccoides = T. turgidum* (L.) Thell ssp. *dicoccoides* Koern) and included 152 lines genotyped with SSR and DArT markers as reported in Peleg et al. [15]. Additional details on the parental lines and the molecular markers mapped in the populations are reported in Table 1.

### Additional component maps used for marker interpolation

An additional set of eight mapping populations (14 maps in total) was used for interpolating additional markers based on the markers common to the consensus framework map. These maps included three double haploid (DH) populations from AAFC Semiarid Prairie Agricultural Research Centre (SPARC; Swift Current, Saskatchewan, Canada), one DH population from USDA-ARS Northern Crop Science Laboratory (Fargo, North Dakota, USA) and two RIL populations from University of Bari (Italy) and ARI, Martonvasar, Hungary. The three DH populations from SPARC were obtained from the crosses Gallareta × Demetra (Gl × Dm, 127 DHs), DT707 × DT696 (127 DHs) and Strongfield × Blackbird (St × Bl 89 DHs). Blackbird is a *Triticum carthlicum* Nevski *in* Kom.), as described by Somers et al. [69]. The fourth DH population, obtained from the cross Lebsock × *T. carthlicum* PI94749 (Lb × P749), included 146 lines and was described in Chu et al. [70]. The RIL population from the University of Bari, obtained from the cross Svevo × Ciccio (Sv × Ci), included 120 F_7_ RILs that were profiled with SSR and expressed sequence tag (EST)-SSRs [71]. The RIL population from ARI, Martonvasar, Hungary, included 182 RILs from the cross PWD1216 × MvTD10-98 that were profiled with DArT and AFLP markers (Karsai et al., unpublished). Details of these additional DH and RIL mapping populations are reported in Table 1. Additionally, selected linkage groups for the populations PDW233 × Bhalegaon 4 (PDW × Bh, [36]) and Latino × Primadur (Lt × Pr, [35]), respectively, and for which no segregation mapping data were available, were also used for marker projection.

### Single map linkage analysis

The segregation mapping data of the mapping populations were used to recalculate the linkage maps using a combination of JoinMap v. 4.0 [31] for marker grouping and Carthagene v. 2.0 [32] for marker ordering and mapping. Low-quality data filtering was carried out as follows: (i) missing data were allowed to a maximum frequency of 0.15, (ii) marker segregation distortion (departure from the expected 1:1 segregation ratio) was allowed up to a probability threshold of *P* =1E-04, corresponding to a segregation distortion not exceeding the 0.7 : 0.3 ratio.

Haldane mapping function was used for all mapping calculations. Marker grouping was performed in JoinMap using the independence LOD method. Robust initial linkage groups (LG) were obtained at LOD = 6.0. The assignment and orientation of linkage groups to wheat chromosomes were carried out by checking for SSR loci common to the hexaploid wheat consensus map (Ta-SSR-2004, http://wheat.pw.usda.gov/GG2/index.shtml) and/or in the Triticarte website (http://www.triticarte.com.au). High quality LG maps were then obtained in Carthagene using stringent mapping parameters. Mapping was carried out through two iterative cycles of: (i) mapping, (ii) investigation of graphical genotypes for the presence of suspicious data points (e.g. single markers that appeared to have recombination events to both flanking marker sides within inter-marker distances of 5 cM or less, mostly derived from unlikely double-crossover events, (iii) checking of raw data and, in case, replacement with missing data. The inter-marker distance threshold for singleton data correction was raised to 10 cM for the DH linkage maps. After stringent mapping and correction of suspicious data, the segregation data belonging to each wheat chromosome were pooled and re-analysed in Carthagene using less stringent grouping thresholds (rec freq = 0.3 LOD = 3) . This produced final maps for each mapping population.

### Construction of the consensus framework map and marker interpolation

The consensus framework map was constructed based on the multipoint maximum likelihood algorithm for consensus mapping, using the integrative function of Carthagene implemented with the *dsmergen* command. This command merges the information from several independent data-sets into a single consensus set by estimating a single recombination rate for each marker pair based on all the available meiosis. The *dsmergen* command only accepts data-sets of homogeneous mapping populations and it was applied to the data set of a marker common to two (or more) individual maps (anchor markers) of the six core component RIL populations (Kf × Sv, Co × Ll, Mr × Cl, Sm × Lv, Kf × UC and Ln × G18-16). Prior to executing the framework mapping, heterogeneity of recombination rate between common marker pairs was tested among all the six core populations by means of the χ^2^ test available in Joinmap v. 4. In cases where strong heterogeneity of recombination rates was detected among populations, a pre-selection of LG from populations with homogeneous recombination rates, suitable for consensus framework mapping, was carried out. Mapping of the anchor markers was carried out in Carthagene with the same procedure used for the single component maps. This procedure produced a robust consensus framework with inter-marker genetic map distances that were estimated across populations.

The loci that were uniquely mapped in all the six component maps and in the other additional eight maps were projected by interpolation following the ‘neighbours’ mapping approach described by Cone et al. [72]. Briefly, the position of a unique locus was projected to the consensus reference map by interpolation of its relative position from the positions of the two closest flanking common markers.

### Linkage disequilibrium and genetic diversity analysis in a panel of elite durum wheat

A panel of 183 durum wheat accessions (mainly cvs. and advanced lines) bred for Mediterranean (Italy, Morocco, Spain, Syria, and Tunisia) countries, Southwestern USA and Mexico were used to demonstrate the utility of the consensus map for characterizing linkage disequilibrium (LD) and to carry out a genome-wide association analysis of loci for time to heading in Mediterranean environments. The collection, hereafter reported as “durum panel”, was assembled and maintained at the University of Bologna (Italy).

The durum panel was characterized with a core set of SSR and DArT markers, mostly included in the consensus map. The accessions were profiled with 350 SSRs and the Triticarte Durum *Pst*I/*Taq*I representation v. 2.0 Array that yielded 900 high-quality polymorphic DArT markers. In total, 1,211 markers were suitable for association mapping (minor allele frequency > 0.10) analysis. Among those, 334 SSR/STS and 623 DArT markers could be projected onto the consensus linkage map and were used for LD and marker-phenotype association tests (957 markers).

The extent of LD between markers and the average LD decay rate in the durum panel were estimated in TASSEL, v. 3.0 (http://www.maizegenetics.net, [73]), based on the consensus marker order and genetic distances. Pairwise LD squared correlation coefficient (*r*^2^) values were estimated for intra-chromosomal (syntenic) loci and were regressed on the pairwise genetic distances (cM). Significance was computed with 10,000 permutations. The decline of LD with distance (recombination rate in Morgans) was estimated by fitting the following equation [74]:


derived from the relationship between LD *r*^2^ values and effective population size (N_e_), with *β* = *k*N_e_:


where *LD*_*i*_ = (*r*^2^ – 1/n) is LD estimate adjusted for chromosome sample size (n). The *d*_*i*_ is the distance in Morgans for the syntenic marker pair *i* as from the consensus map. The *c* value is the recombination rate. The *k* value = 4 for autosomes. For curve fitting the parameter α was set to 1 (no mutations). Non-linear regression modeling was performed in Lab Fit Curve fitting software v. 7. The e_*i*_ residuals were estimated by non-linear fitting of the model; the α and *β* parameters were estimated iteratively by least squares method. The 95^th^ percentile of the LD *r*^2^ values distribution between non-syntenic markers was used as a threshold to distinguish the LD mostly attributable to linkage from the background LD caused by population structure [75].

Prior knowledge of the population structure of the durum panel was available from a previous analysis of 96 highly informative and evenly spread SSRs with two complementary cluster analysis methods: i) a distance-based analysis (NTSys software) and ii) a model-based quantitative cluster analyses (STRUCTURE software). Results are detailed in Maccaferri et al. [38] and in Letta et al. [20]. Briefly, the 183 durum accessions were grouped into five main sub-populations corresponding to breeding lineages as follows: (1) the ICARDA germplasm bred for the dryland areas, related to the West-Asian local landraces, (2) the ICARDA germplasm bred for the temperate areas; 3) the Italian and early-period (1970s) CIMMYT germplasm, (4) the mid-period CIMMYT germplasm (1980s), (5) the late-period CIMMYT germplasm (from late 1980s onwards). In total, 11, 55, 26, 56 and 35 accessions were associated to sub-populations 1 to 5, respectively (STRUCTURE analysis).

The genetic variation within and among the five sub-populations was assessed by analysis of molecular variance (AMOVA) and F_ST_ statistics in ARLEQUIN v 3.5 [76], using all the informative and mapped SSR and DArT marker genotype data available at one level of population hierarchy. One thousand permutations of accessions within sub-populations were used to estimate the significance of the differences. To further investigate the differentiation of sub-populations at the chromosome region level, single locus-AMOVA analysis and F_ST_ statistic calculation was performed for all sub-population pairwise comparisons (ten in total). Highly significant (*P* ≤ 0.01) F_ST_ values were plotted on the durum consensus map. The overall polymorphism information content (PIC) index of each locus, after standardization for the number of alleles [30], was also plotted on the durum consensus map.

### Association mapping for heading date across European and Mediterranean environments

Heading date records of the durum panel were available for 27 field trials carried out in multiple environments from 2003 to 2007 from (listed by decreasing latitude) Northern to Southern Italy and Spain for Europe, Tunisia and Morocco for North Africa, Syria and Lebanon for West Asia and Mexico. Single field trials (hereafter considered as single environments) were carried out based on a modified augmented design layout with three replicated checks (by rows and columns) and unreplicated accession plots of 4 m^2^. All trials were fall-sown (October to December) and managed according to locally adopted agronomic practices. Further details are reported in Maccaferri et al. [38]. Phenotypic data were analysed in SAS statistical package, PROCMIXED procedure, by restricted maximum likelihood (REML) to fit a mixed model with checks as a fixed effect and unreplicated entries as random effects, as in Maccaferri et al. [12, 38]. The best linear unbiased predictors (BLUPs) from the random model (REML variance component estimation) were used in AM analyses. The degree of relationships among environments was studied through an additive main effects and multiplicative interaction (AMMI) analysis coupled with biplot for visual representation in Genstat 16.0. Based on the results of the biplot analysis, groups of environments with uniform patterns of heading date were identified, corresponding to five macro-environmental areas. A combined analysis was carried out for each of the five main macro-environmental areas. Adjusted phenotypic means and heritability values (*h*^2^) were calculated across environments for each macro-environmental area. Broad-sense *h*^2^ values were calculated according to the formula:


where *n* is the number of environments.

Genome-wide association scan (GWAS) for loci governing heading date was conducted using single-environment BLUPs and adjusted means for each of the five macro-environmental groups in TASSEL program v 4.0 (http://www.maizegenetics.net), based on a compressed mixed linear model (MLM) that included the 5 × 183 *Q* main population structure estimate matrix from STRUCTURE analysis of the 96 non-redundant highly informative SSR markers and a 183 × 183 *K* kinship matrix (*Q* + *K* MLM; [73]). The kinship matrix was obtained using a simple-matching genetic similarity algorithm to estimate the identity by state (IBS) portion of the genome between all pairs of accessions [77]. For the association test, SSR alleles were converted to bi-allelic data and the association was performed by testing each allele (with MAF > 0.10) *vs*. the pool of all the other alleles. For the DArT markers, the test was carried out by contrasting the ‘allele presence’ status *vs*. the ‘allele absence’ status of two accession pools. Besides the marker-wise significance calculated based on the F test, the experiment-wise significance was set by counting the genome-wise number of unique marker linkage blocks; this was accomplished by using the consensus map order and the LD *r*^2^ threshold of 0.3 to define the LD blocks between adjacent markers (this threshold is widely recognized for partitioning the LD “useful” for mapping in experimental designs for continuous traits, [53, 78, 79]). A Bonferroni-corrected, experiment-wise significance threshold of *P* ≤ 0.05 was set by taking into account the genome-wide number of unique linkage blocks.

Because the results of the first run of GWAS analysis showed that control of heading date in the durum panel was dominated by the two *PPD-1* homeologs (*Ppd-A1* and *Ppd-B1*), GWAS targeted to small-effect loci was repeated with the *Ppd-A1* and *Ppd-B1* representative markers included as additional covariates in the mixed linear model. Due to the overall high stringency of the experiment-wise significance threshold (the *P*_*exp*_ ≤ 0.05 corresponded to a *P*_*marker*_ ≤ 0.0001 marker-wise), small-effect QTL regions were also reported for those markers/chromosome regions with highly significant (P ≤ 0.01) marker-wise associations to the adjusted phenotypic means of one or more macro-environmental area.

### Availability of supporting data

All the supporting data are included as additional files.

## Electronic supplementary material

Additional file 1: Figure S1: Representations of the consensus linkage map and of the six core component maps. Graphical representation of the consensus linkage map and of the six core linkage maps used to produce the consensus framework. The consensus linkage map is on the right side of the linkage graphics. Anchor (common) markers are in red font. Markers common to three or more maps are interconnected by dashed lines. (PPTX 3 MB)

Additional file 2: Table S1: List of the markers included in the consensus linkage map. The markers included in the consensus interpolated map are reported together with their chromosome and map distances. Anchor (common) markers are highlighted in bold. Unique (singleton) markers projected onto the consensus from single specific maps are reported together with the indication of the specific maps (cell background colours). (XLSX 84 KB)

Additional file 3: Figure S2: Projection plots of the six core component maps on the consensus. The core component linkage group maps are reported in the X axes of the projection plots while the consensus map is reported in the Y axes. Spearman rank correlations are reported as ro (*ρ*) coefficients. (PPTX 3 MB)

Additional file 4: Figure S3A: Projection plots of the tetraploid wheat consensus map on the hexaploid SSR reference map (Ta-SSR-2004). (PPTX 404 KB)

Additional file 5: Figure S3B: Projection plots of the tetraploid wheat consensus map on the tetraploid mappreviously reported by Marone et al. [19]. (PPTX 149 KB)

Additional file 6: Figure S4: Detailed pairwise linkage disequilibrium plots, distribution of loci with highly significant Fst differentiation patterns and of the polymorphism index content values of SSR and DArT^®^ markers along the tetraploid wheat consensus map, as estimated from the elite durum wheat germplasm accessions from world-wide. Pairwise linkage disequilibrium (LD) patterns are shown for the squared coefficients of determination (*r* ^2^) values between loci used to profile a panel of 183 elite durum accessions and mapped on the consensus map (left side of chromosome bars). Loci with highly significant F_ST_ differentiation values between durum sub-population groups are reported on the right side of the chromosome bars, as long with the locus polymorphism index content (PIC) plots, normalized for allele number. (PPTX 7 MB)

Additional file 7: Table S2: GWAS results for heading date in the elite Durum Panel across 27 Mediterranean and Mexican environments using *Ppd-A1* and *Ppd-B1* representative markers as covariates in the mixed linear model. Marker-trait associations, chromosome locations and significance intervals based on the consensus map, *P*-values and (*R* ^2^) coefficients of determination for the chromosome regions harboring QTLs significant for heading date adjusted means from field experiments conducted on five different macro-environment areas (Southern Europe with 10 field experiments [environments], North Africa (Tunisia) with 2 envs., West Asia with 8 envs., North Africa (Morocco) with 2 envs. and Mexico with 5 envs.). A QTL was declared when a marker-wise highly significant effect (P ≤ 0.01) was detected on the adj. means for at least one macro-environmental area. *R* ^2^ values are reported in brackets next to the *P*-values. The number of single environments where the QTLs showed significant effects is also reported for each macro-area. Macro-area are sorted from left to right based on decreasing latitude. (DOCX 47 KB)
