# Supplementary material for: A consensus framework map of durum wheat (Triticum durum Desf.) suitable for linkage disequilibrium analysis and genome-wide association mapping
Source: BMC Genomics. 2014 Oct 7;15(1):873. doi: 10.1186/1471-2164-15-873 (PMC4287192; doi:10.1186/1471-2164-15-873)

## Slide 1
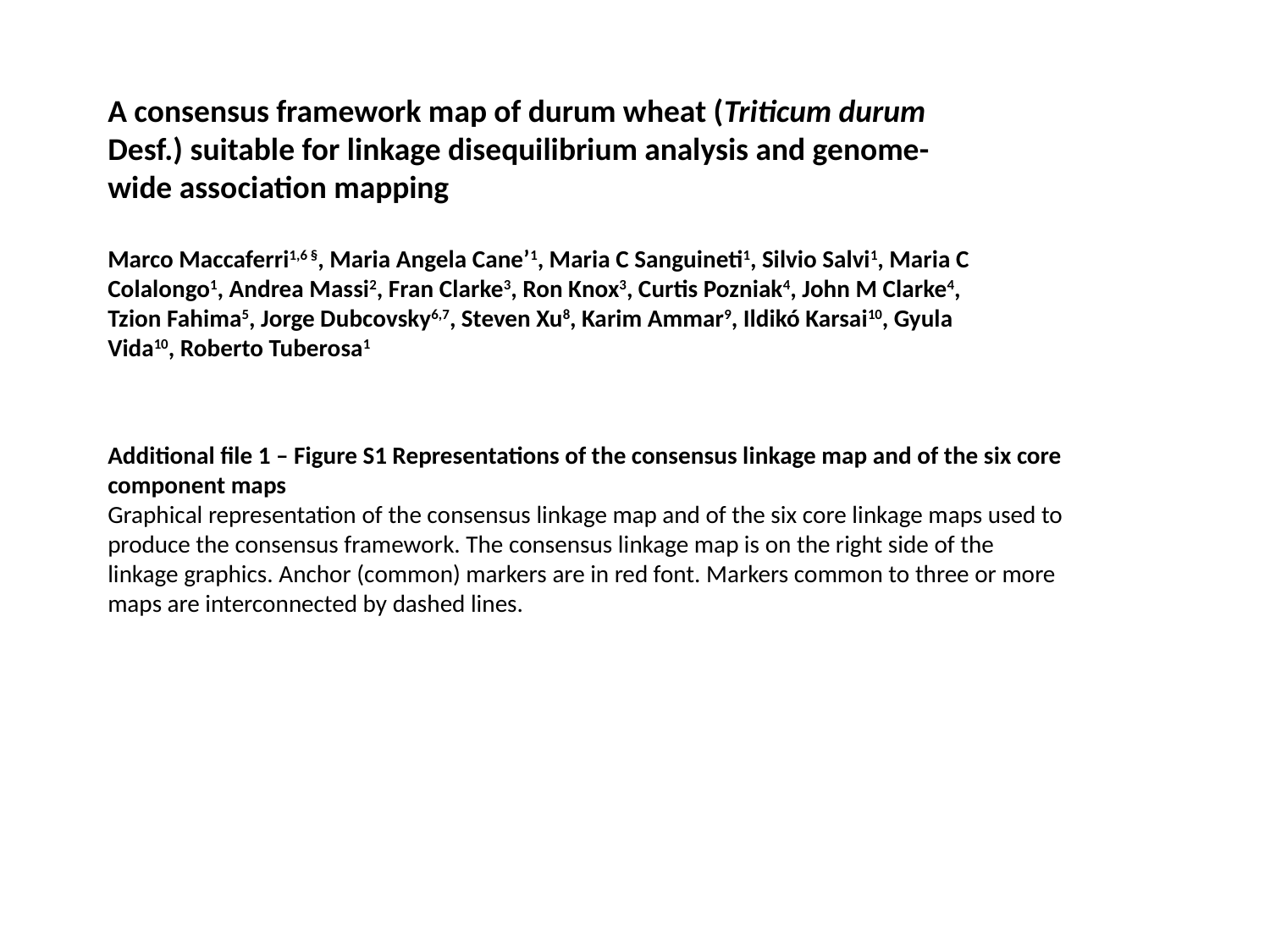

A consensus framework map of durum wheat (Triticum durum Desf.) suitable for linkage disequilibrium analysis and genome-wide association mapping
Marco Maccaferri1,6 §, Maria Angela Cane’1, Maria C Sanguineti1, Silvio Salvi1, Maria C Colalongo1, Andrea Massi2, Fran Clarke3, Ron Knox3, Curtis Pozniak4, John M Clarke4, Tzion Fahima5, Jorge Dubcovsky6,7, Steven Xu8, Karim Ammar9, Ildikó Karsai10, Gyula Vida10, Roberto Tuberosa1
Additional file 1 – Figure S1 Representations of the consensus linkage map and of the six core component maps
Graphical representation of the consensus linkage map and of the six core linkage maps used to produce the consensus framework. The consensus linkage map is on the right side of the linkage graphics. Anchor (common) markers are in red font. Markers common to three or more maps are interconnected by dashed lines.

## Slide 2
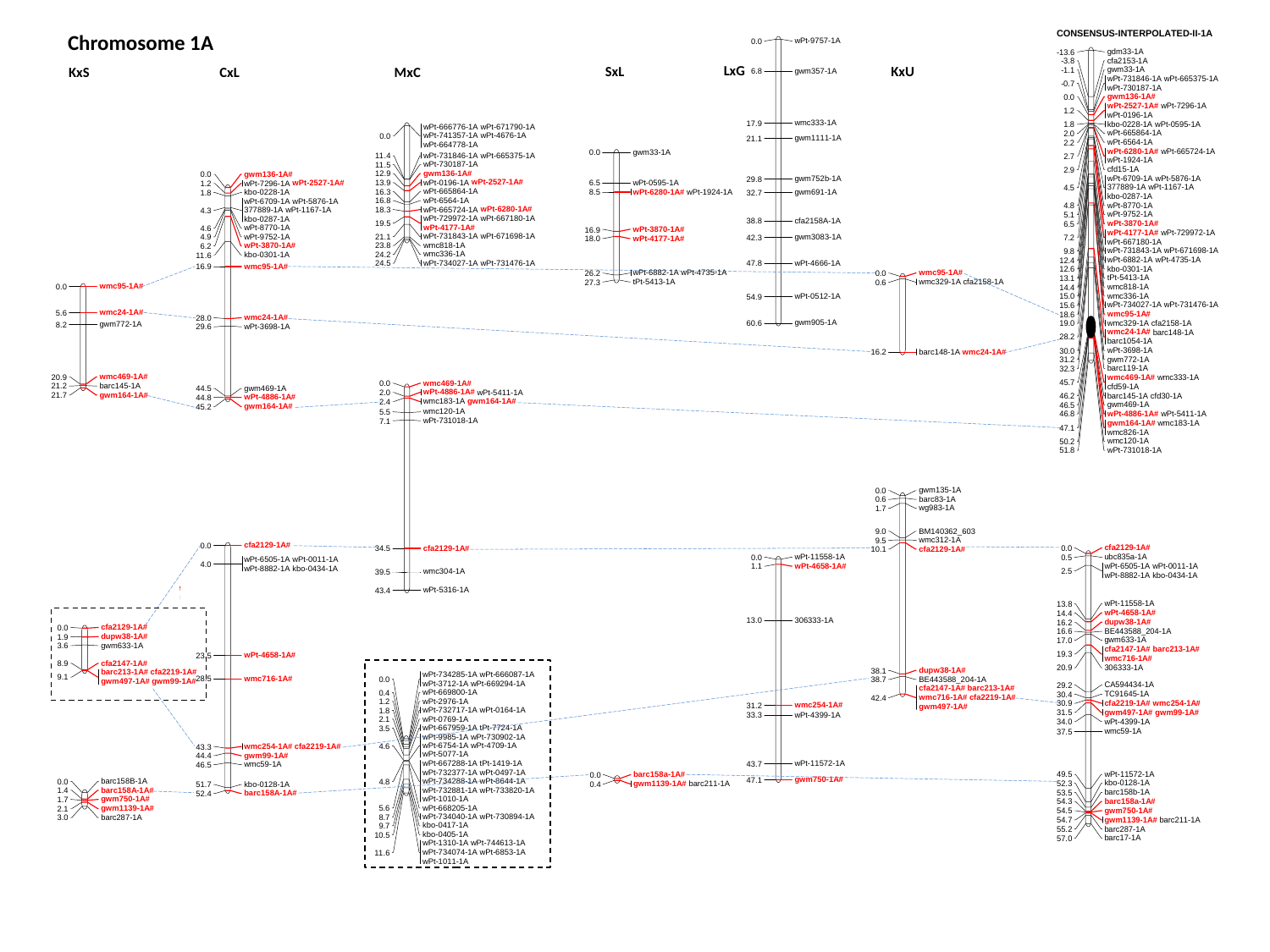

Chromosome 1A
LxG
SxL
KxU
MxC
KxS
CxL

## Slide 3
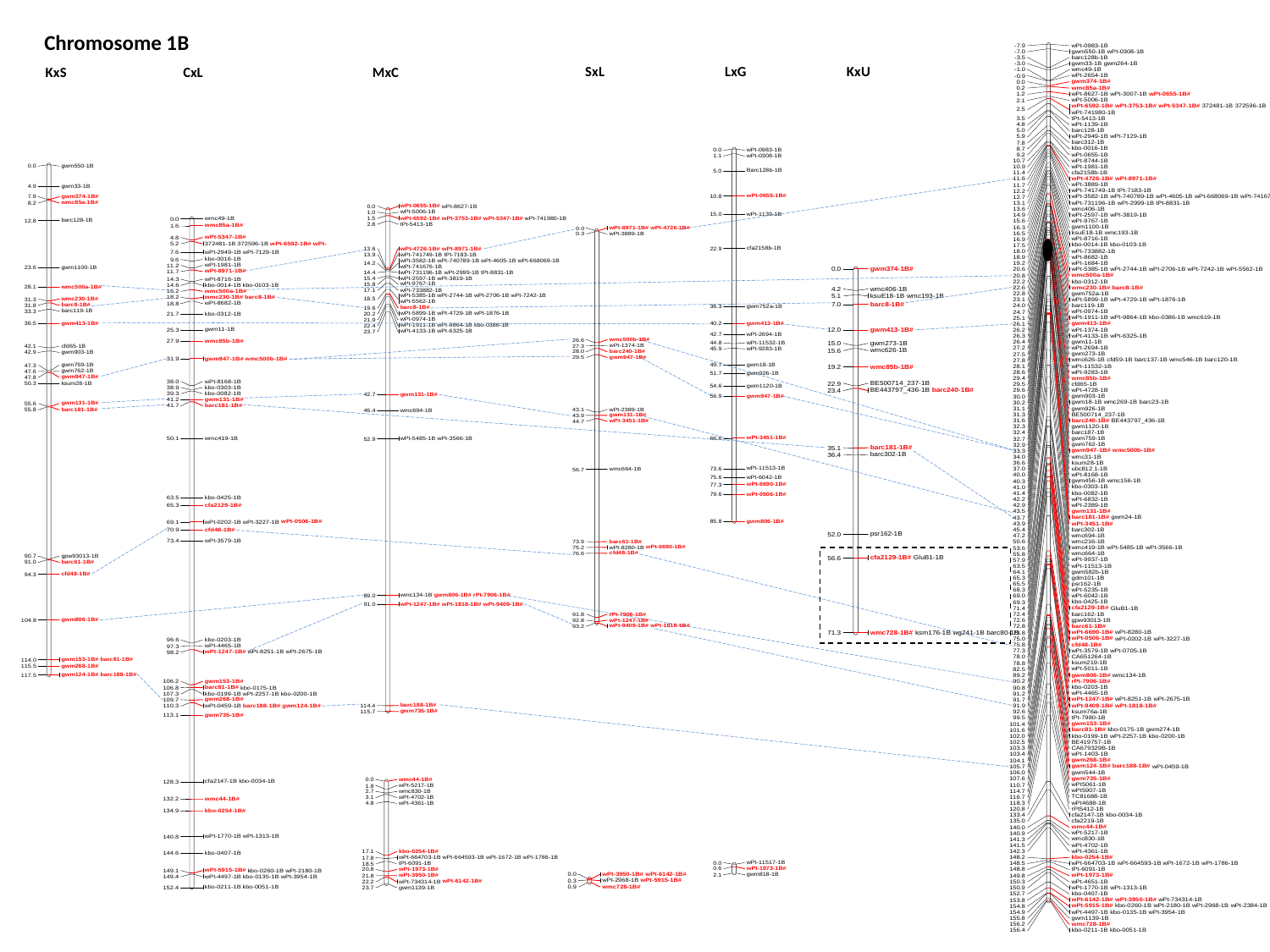

Chromosome 1B
SxL
LxG
KxU
MxC
KxS
CxL

## Slide 4
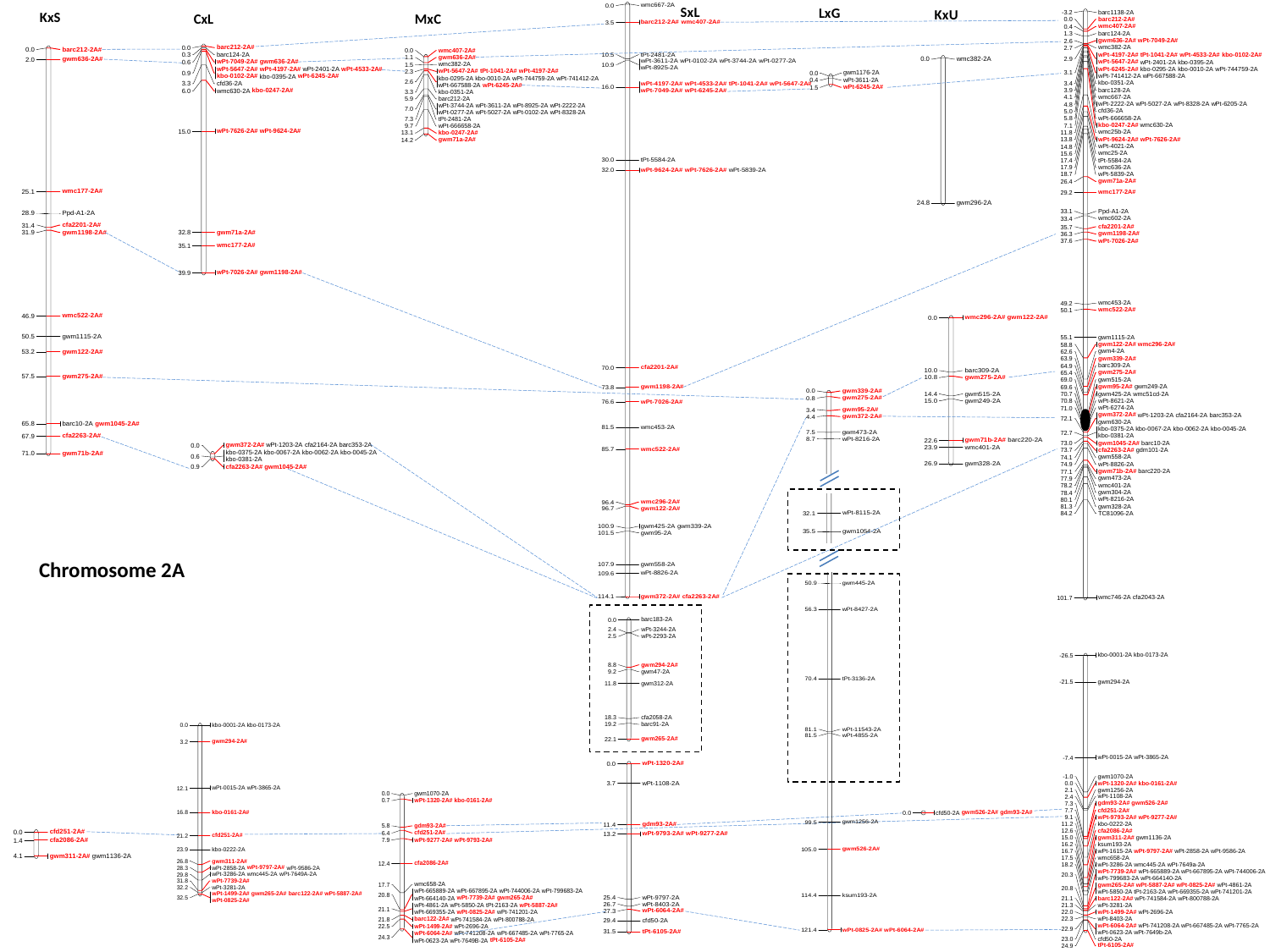

KxU
SxL
LxG
KxS
CxL
MxC
Chromosome 2A

## Slide 5
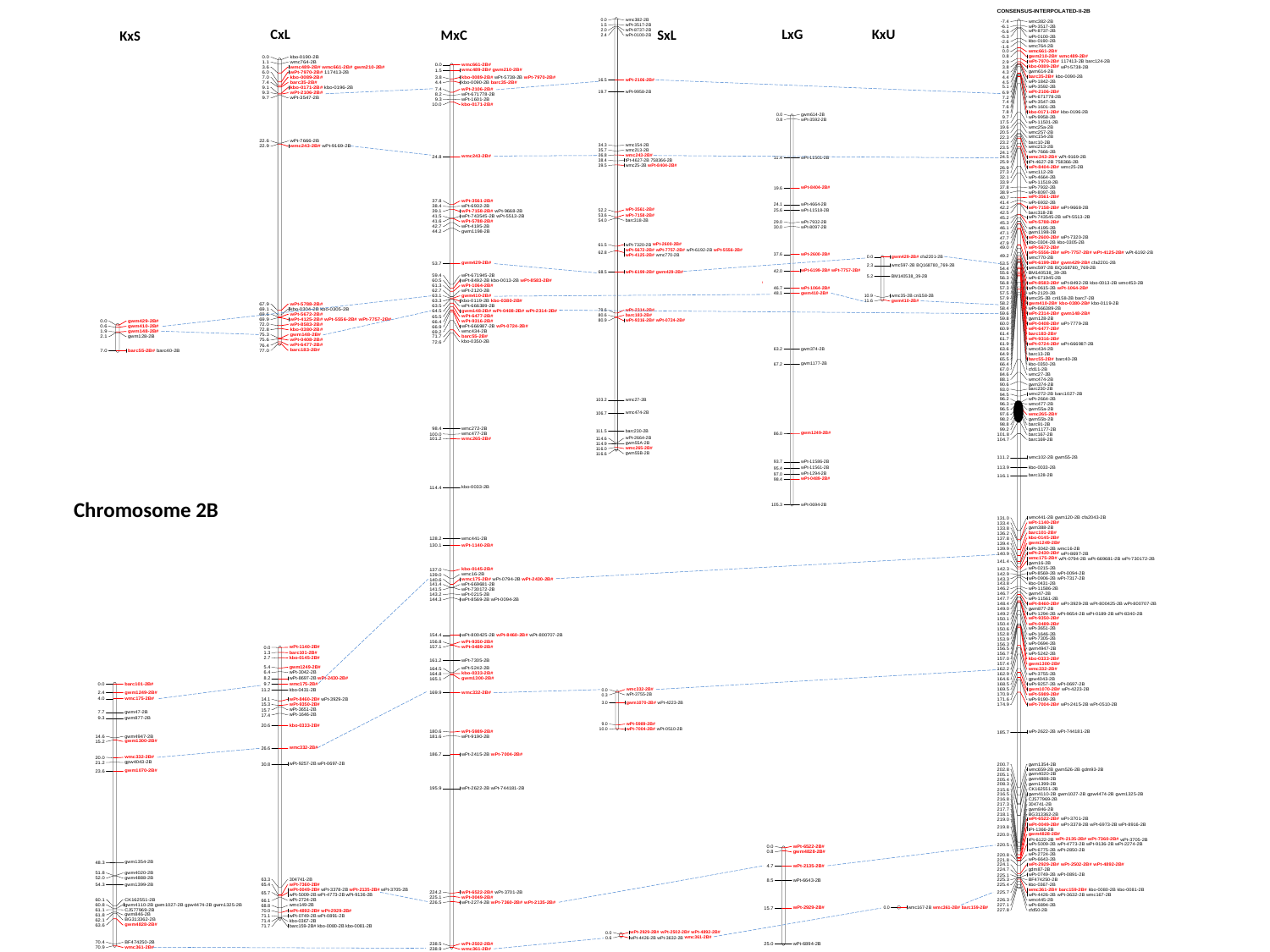

LxG
CxL
KxU
MxC
SxL
KxS
Chromosome 2B

## Slide 6
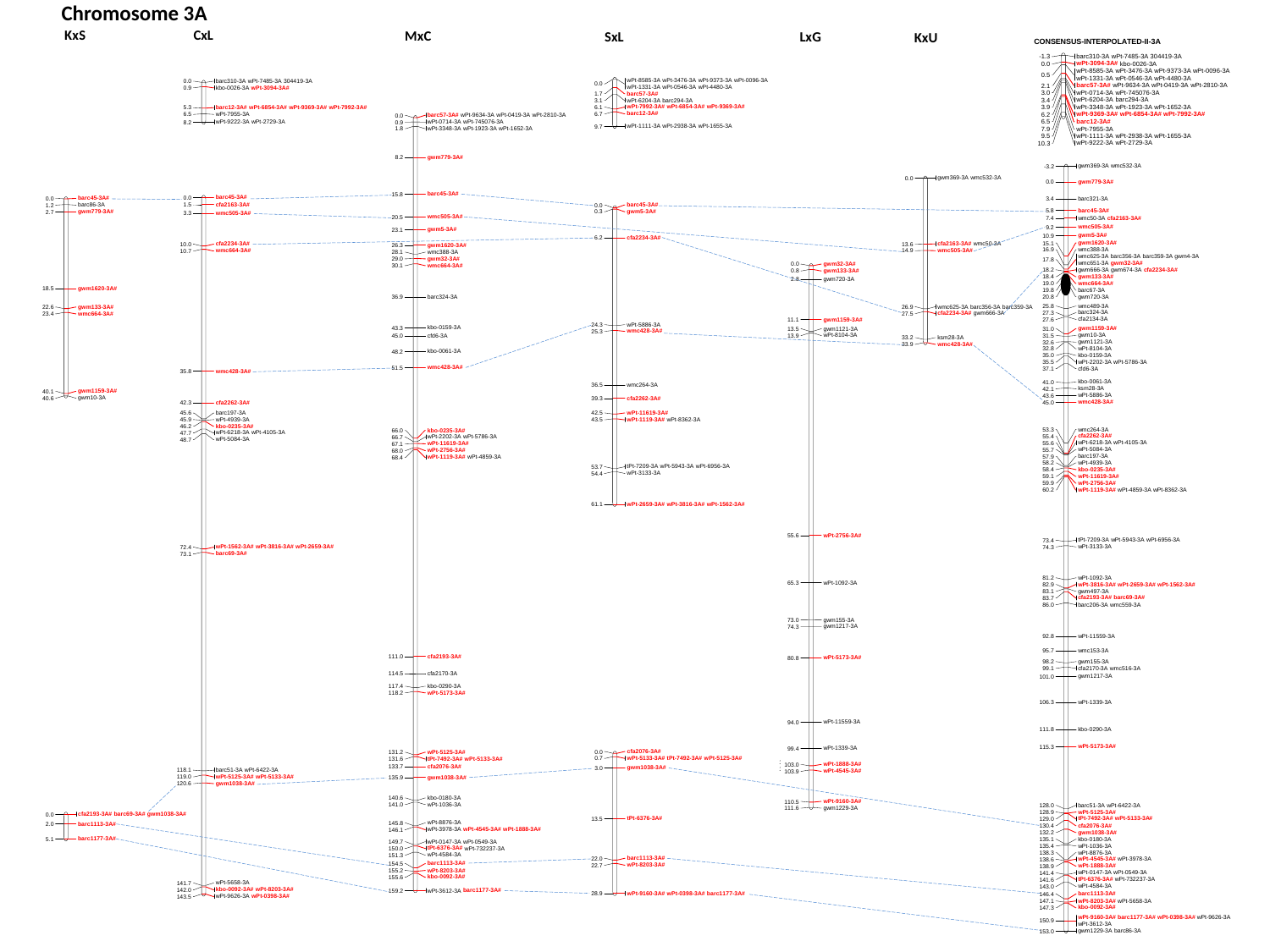

Chromosome 3A
CxL
KxS
MxC
SxL
LxG
KxU

## Slide 7
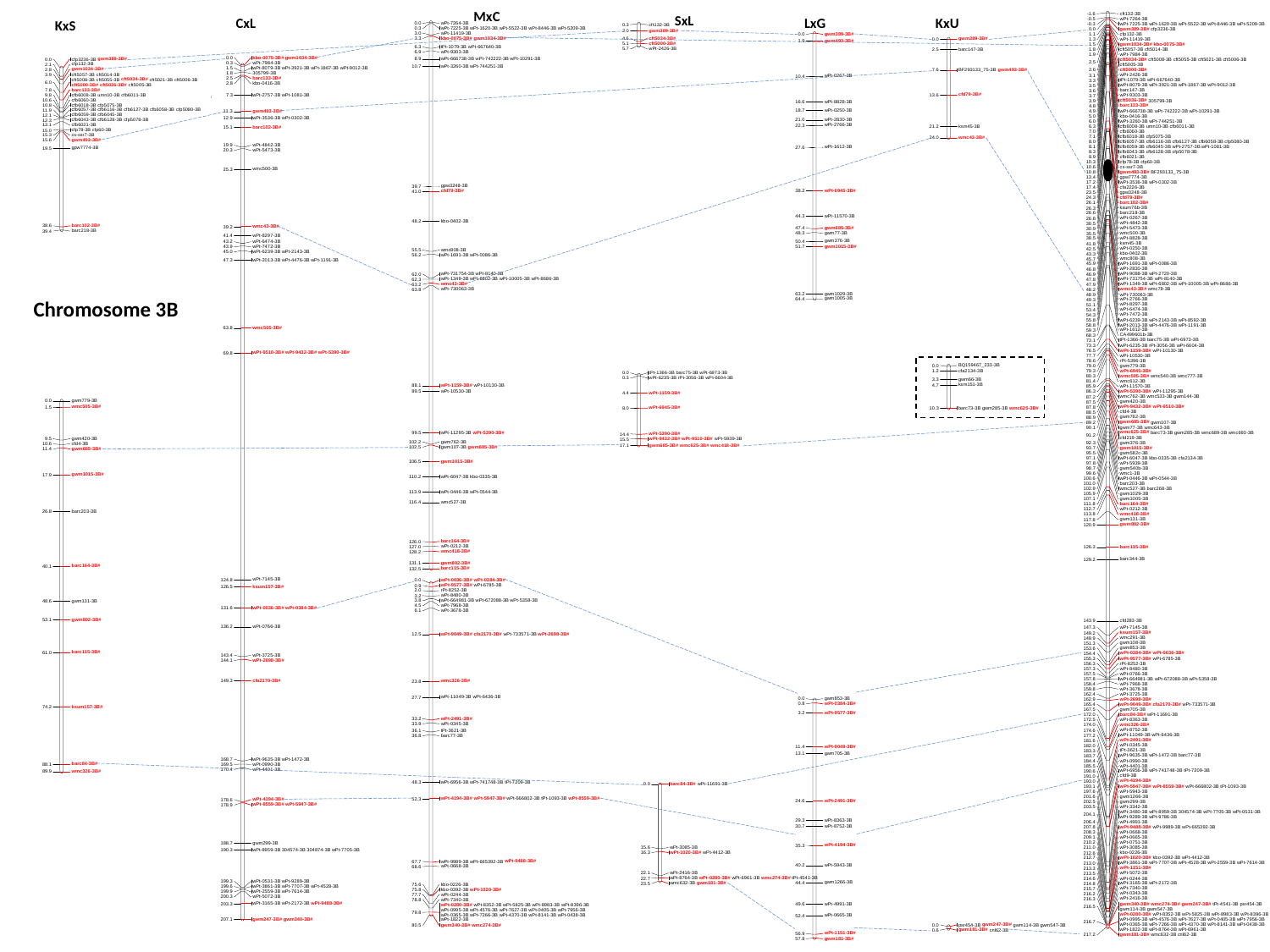

MxC
SxL
LxG
CxL
KxU
KxS
Chromosome 3B

## Slide 8
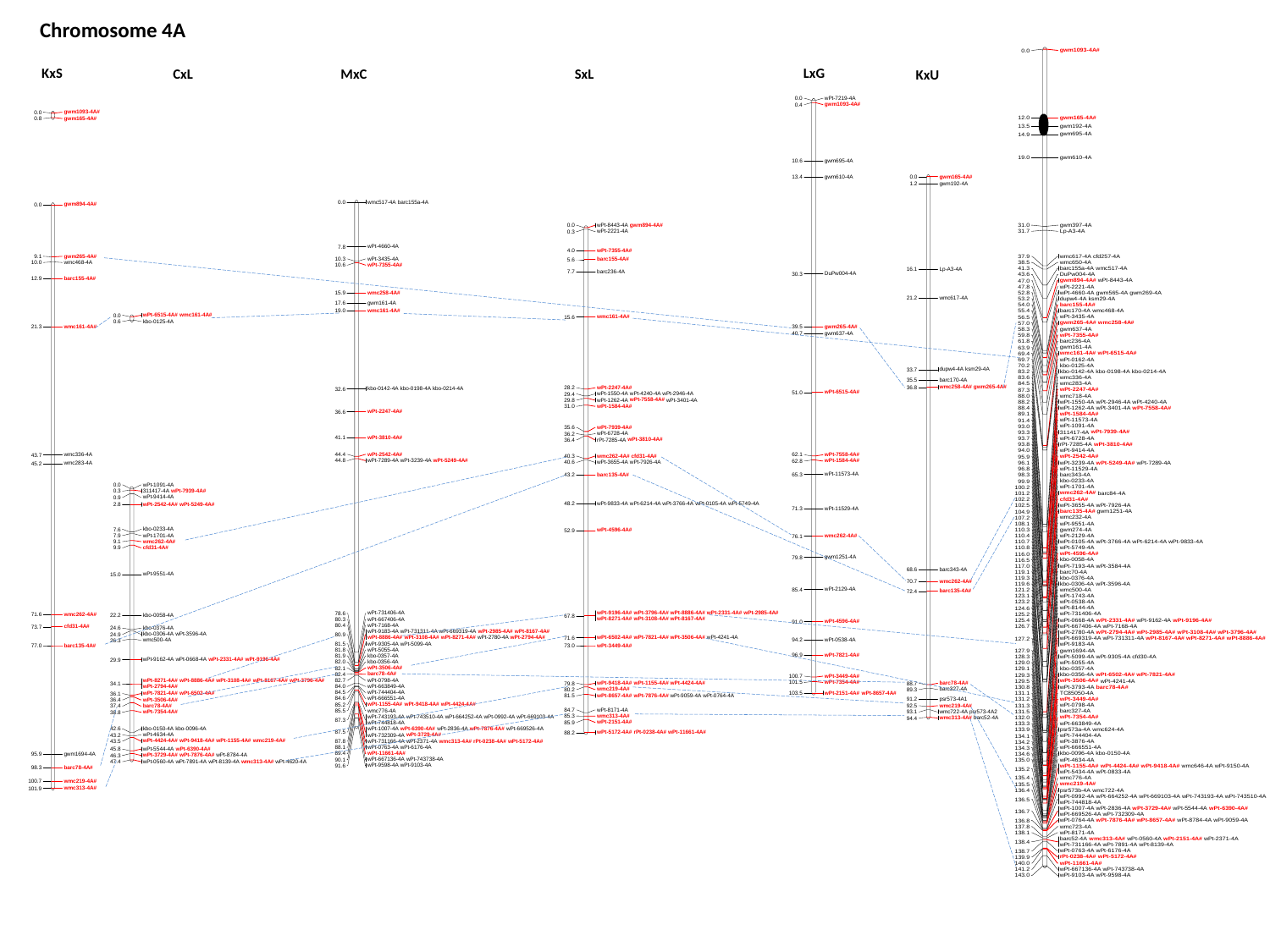

Chromosome 4A
LxG
KxS
CxL
MxC
SxL
KxU

## Slide 9
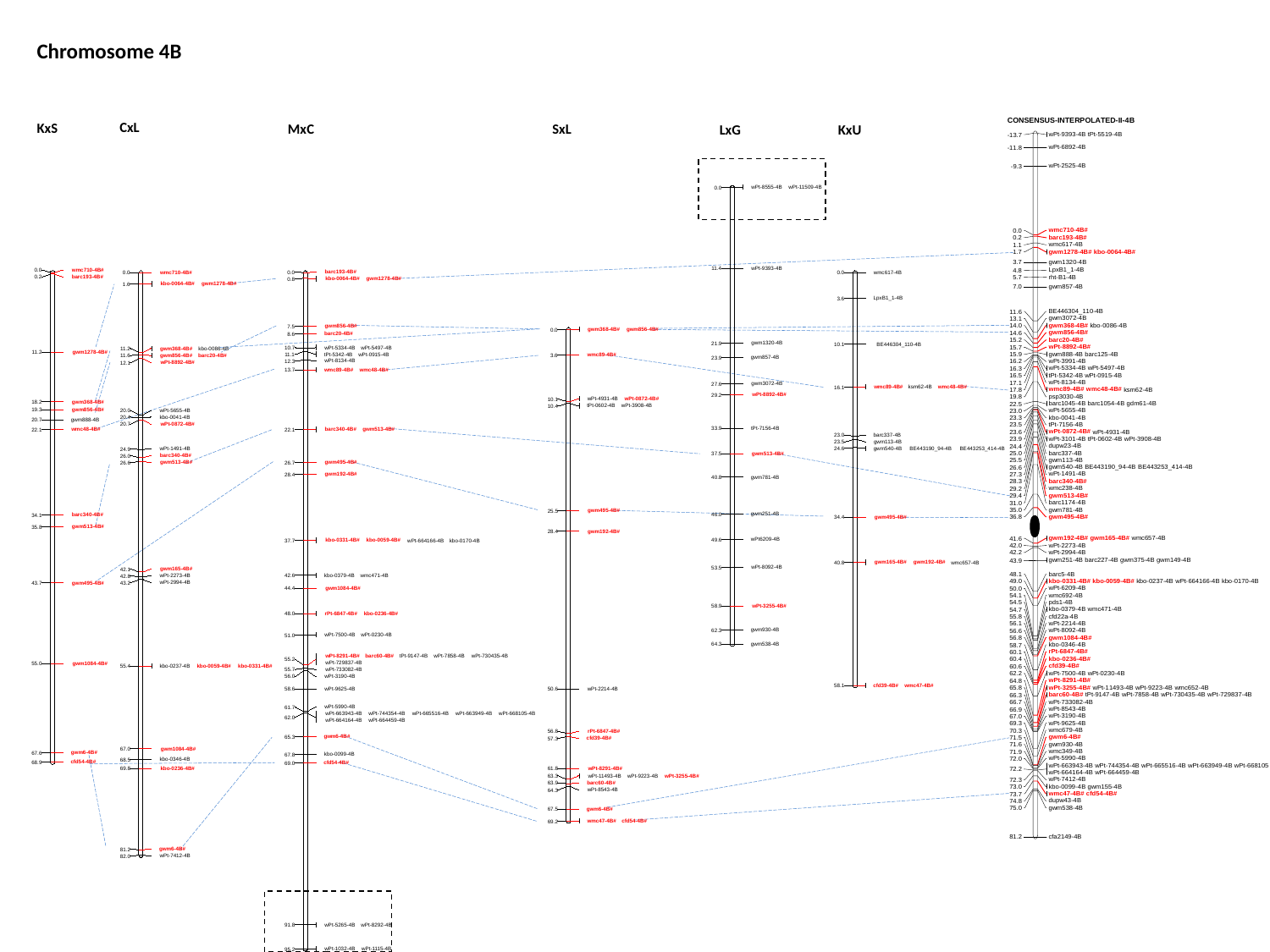

Chromosome 4B
CxL
KxS
MxC
SxL
LxG
KxU
wPt-8555-4B
wPt-11509-4B
0.0
wPt-9393-4B
11.4
gwm1320-4B
21.9
gwm857-4B
23.9
gwm3072-4B
27.6
wPt-8892-4B#
29.2
tPt-7156-4B
33.9
gwm513-4B#
37.5
gwm781-4B
40.8
gwm251-4B
46.0
wPt6209-4B
49.6
wPt-8092-4B
53.5
wPt-3255-4B#
58.9
gwm930-4B
62.3
gwm538-4B
64.3
wmc710-4B#
0.0
barc193-4B#
0.2
gwm1278-4B#
11.2
gwm368-4B#
18.2
gwm856-4B#
19.3
gwm888-4B
20.7
wmc48-4B#
22.1
barc340-4B#
34.1
gwm513-4B#
35.8
gwm495-4B#
43.7
gwm1084-4B#
55.0
gwm6-4B#
67.6
cfd54-4B#
68.9
barc193-4B#
0.0
kbo-0064-4B#
gwm1278-4B#
0.8
gwm856-4B#
7.5
barc20-4B#
8.6
wPt-5334-4B
wPt-5497-4B
10.7
tPt-5342-4B
wPt-0915-4B
11.1
wPt-8134-4B
12.3
wmc89-4B#
wmc48-4B#
13.7
barc340-4B#
gwm513-4B#
22.1
gwm495-4B#
26.7
gwm192-4B#
28.4
kbo-0331-4B#
kbo-0059-4B#
wPt-664166-4B
kbo-0170-4B
37.7
kbo-0379-4B
wmc471-4B
42.6
gwm1084-4B#
44.4
rPt-6847-4B#
kbo-0236-4B#
48.0
wPt-7500-4B
wPt-0230-4B
51.0
wPt-8291-4B#
barc60-4B#
tPt-9147-4B
wPt-7858-4B
wPt-730435-4B
55.2
wPt-729837-4B
wPt-733082-4B
55.7
wPt-3190-4B
56.0
wPt-9625-4B
58.6
wPt-5990-4B
61.7
wPt-663943-4B
wPt-744354-4B
wPt-665516-4B
wPt-663949-4B
wPt-668105-4B
62.0
wPt-664164-4B
wPt-664459-4B
gwm6-4B#
65.3
kbo-0099-4B
67.8
cfd54-4B#
69.0
wPt-5265-4B
wPt-8292-4B
91.8
wPt-1032-4B
wPt-1115-4B
95.2
wmc710-4B#
0.0
kbo-0064-4B#
gwm1278-4B#
1.6
gwm368-4B#
kbo-0086-4B
11.2
gwm856-4B#
barc20-4B#
11.6
wPt-8892-4B#
12.1
wPt-5655-4B
20.0
kbo-0041-4B
20.4
wPt-0872-4B#
20.7
wPt-1491-4B
24.9
barc340-4B#
26.0
gwm513-4B#
26.6
gwm165-4B#
42.1
wPt-2273-4B
42.8
wPt-2994-4B
43.2
kbo-0059-4B#
kbo-0331-4B#
kbo-0237-4B
55.4
gwm1084-4B#
67.0
kbo-0346-4B
68.5
kbo-0236-4B#
69.8
gwm6-4B#
81.2
wPt-7412-4B
82.0
wmc617-4B
0.0
LpxB1_1-4B
3.6
BE446304_110-4B
10.1
wmc89-4B#
wmc48-4B#
ksm62-4B
16.1
barc337-4B
23.0
gwm113-4B
23.5
gwm540-4B
BE443190_94-4B
BE443253_414-4B
24.6
gwm495-4B#
34.4
gwm165-4B#
gwm192-4B#
wmc657-4B
40.8
cfd39-4B#
wmc47-4B#
58.1
gwm368-4B#
gwm856-4B#
0.0
wmc89-4B#
3.6
wPt-0872-4B#
wPt-4931-4B
10.1
tPt-0602-4B
wPt-3908-4B
10.4
gwm495-4B#
25.5
gwm192-4B#
28.4
wPt-2214-4B
50.6
rPt-6847-4B#
56.8
cfd39-4B#
57.3
wPt-8291-4B#
61.8
wPt-11493-4B
wPt-9223-4B
wPt-3255-4B#
63.3
barc60-4B#
63.9
wPt-8543-4B
64.3
gwm6-4B#
67.5
wmc47-4B#
cfd54-4B#
69.2

## Slide 10
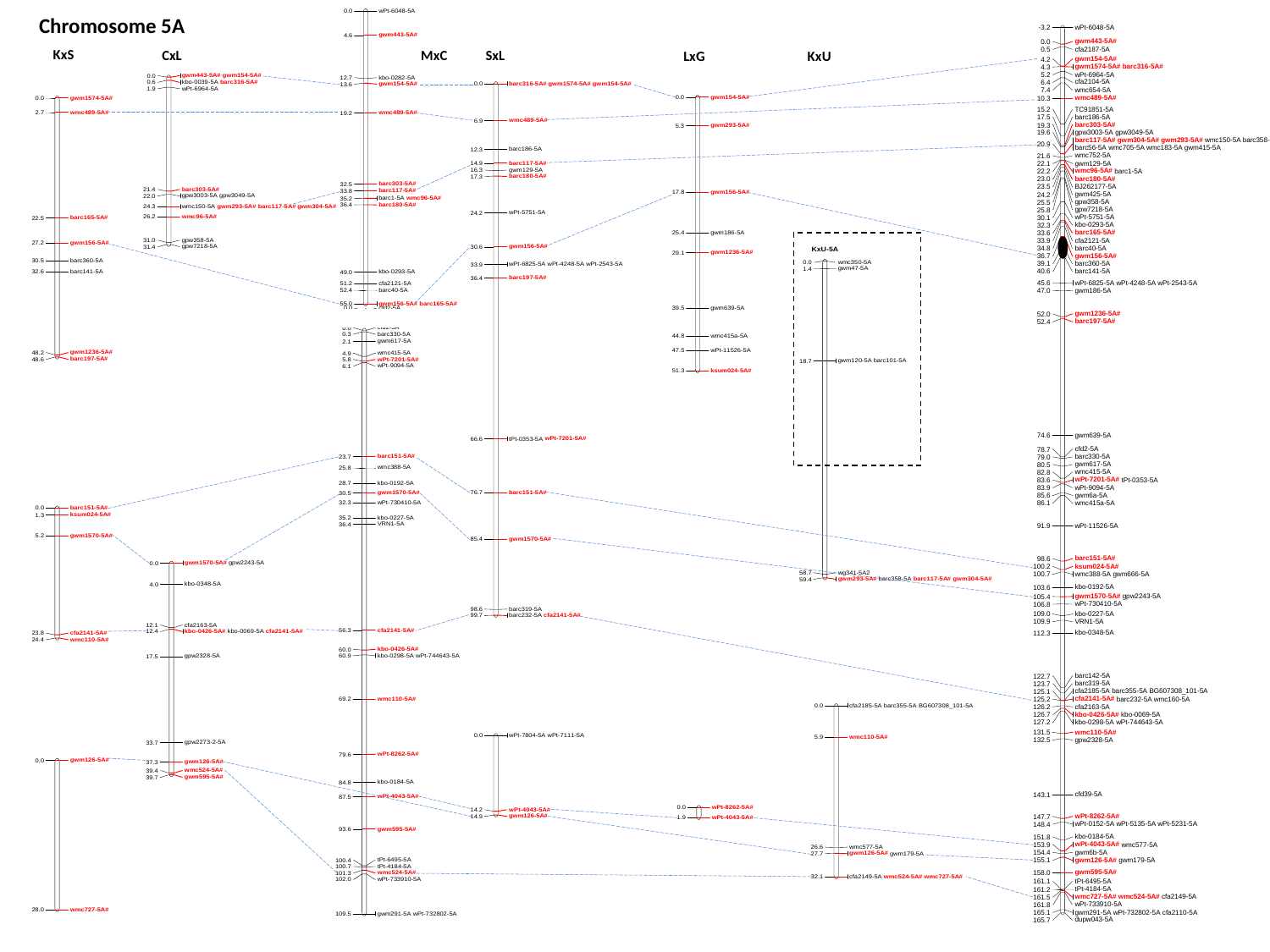

Chromosome 5A
KxS
CxL
MxC
SxL
LxG
KxU

## Slide 11
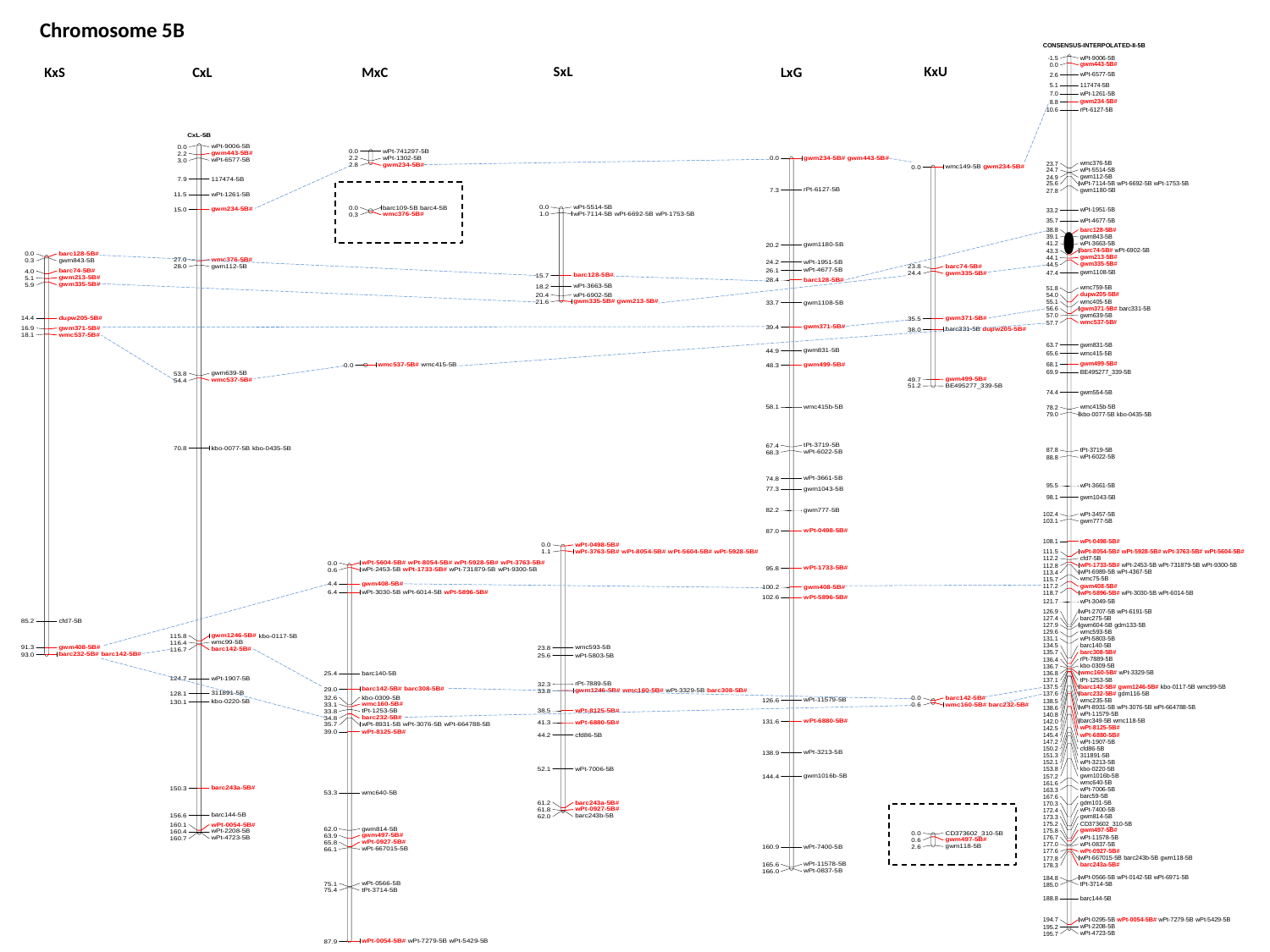

Chromosome 5B
SxL
KxU
MxC
KxS
CxL
LxG

## Slide 12
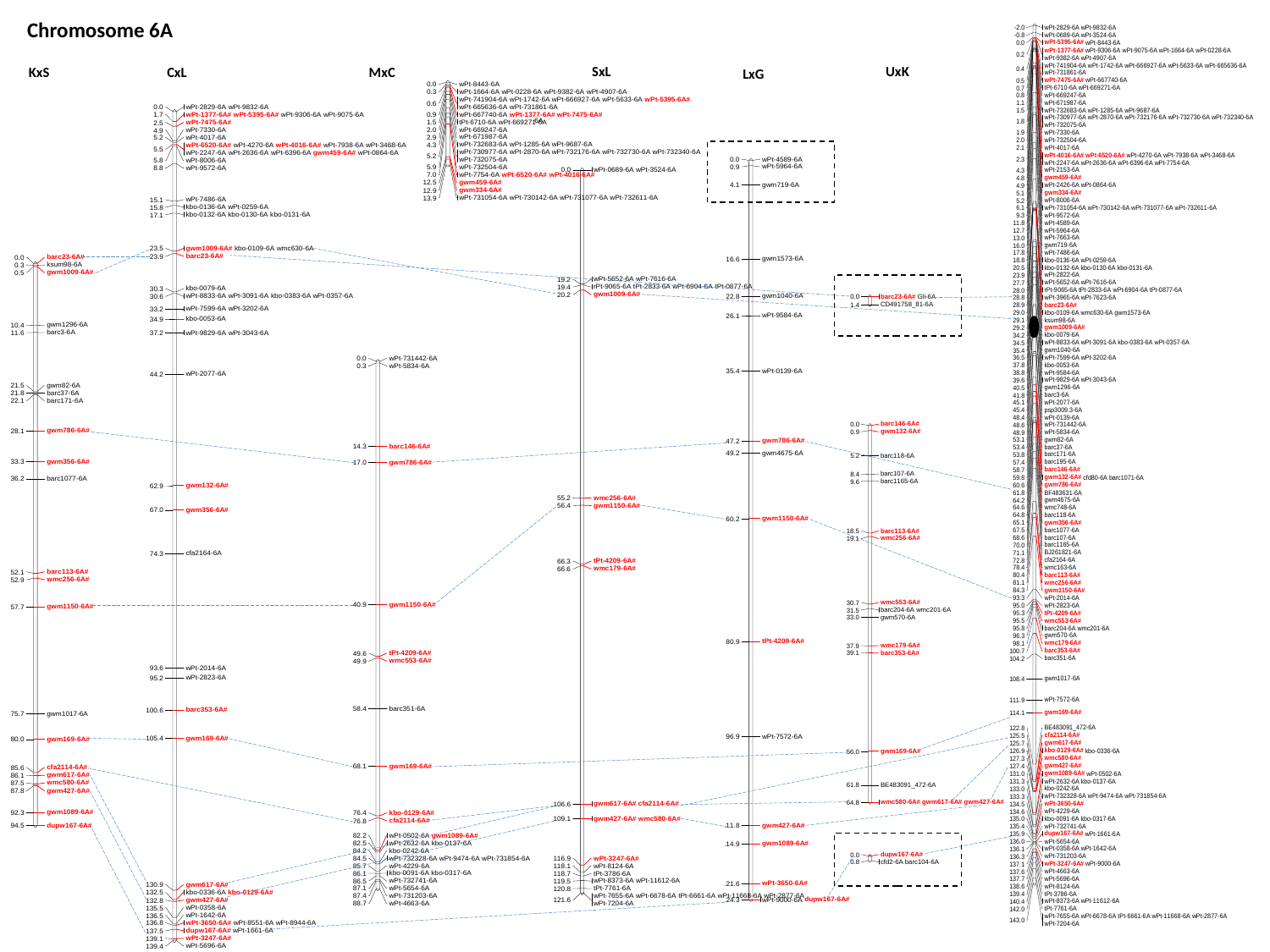

Chromosome 6A
SxL
UxK
MxC
KxS
CxL
LxG

## Slide 13
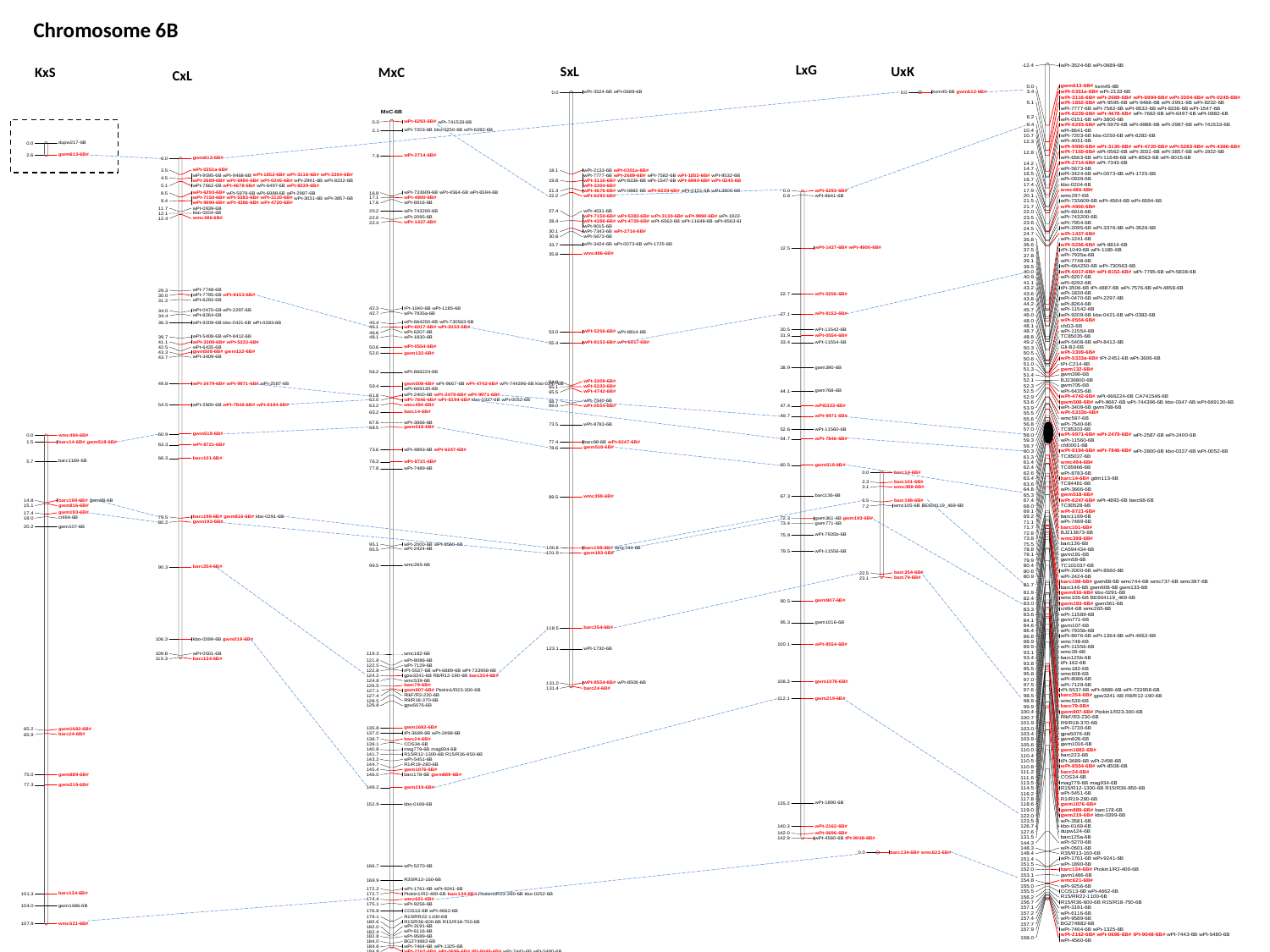

Chromosome 6B
LxG
SxL
UxK
MxC
KxS
CxL

## Slide 14
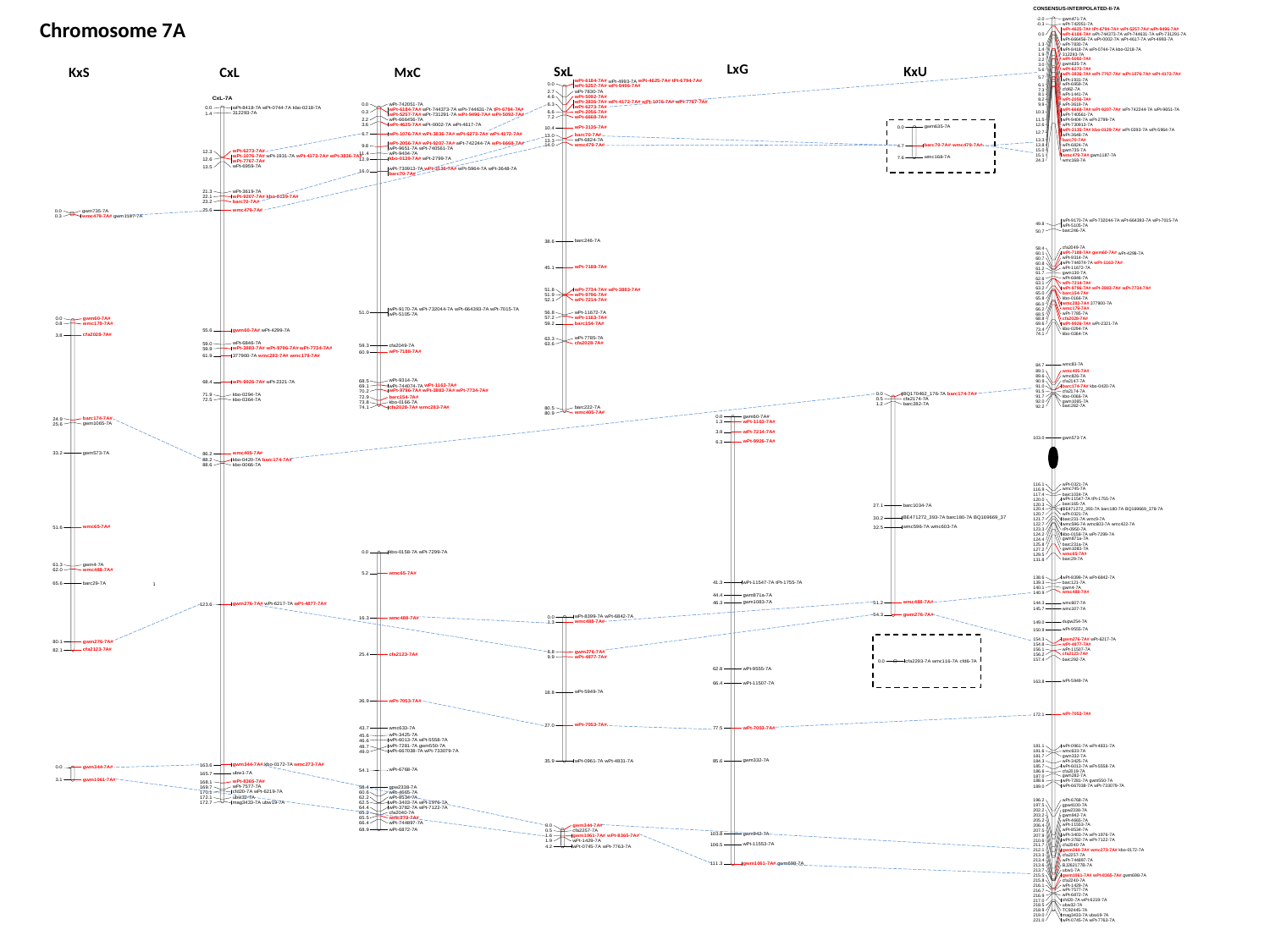

Chromosome 7A
LxG
SxL
KxU
MxC
KxS
CxL

## Slide 15
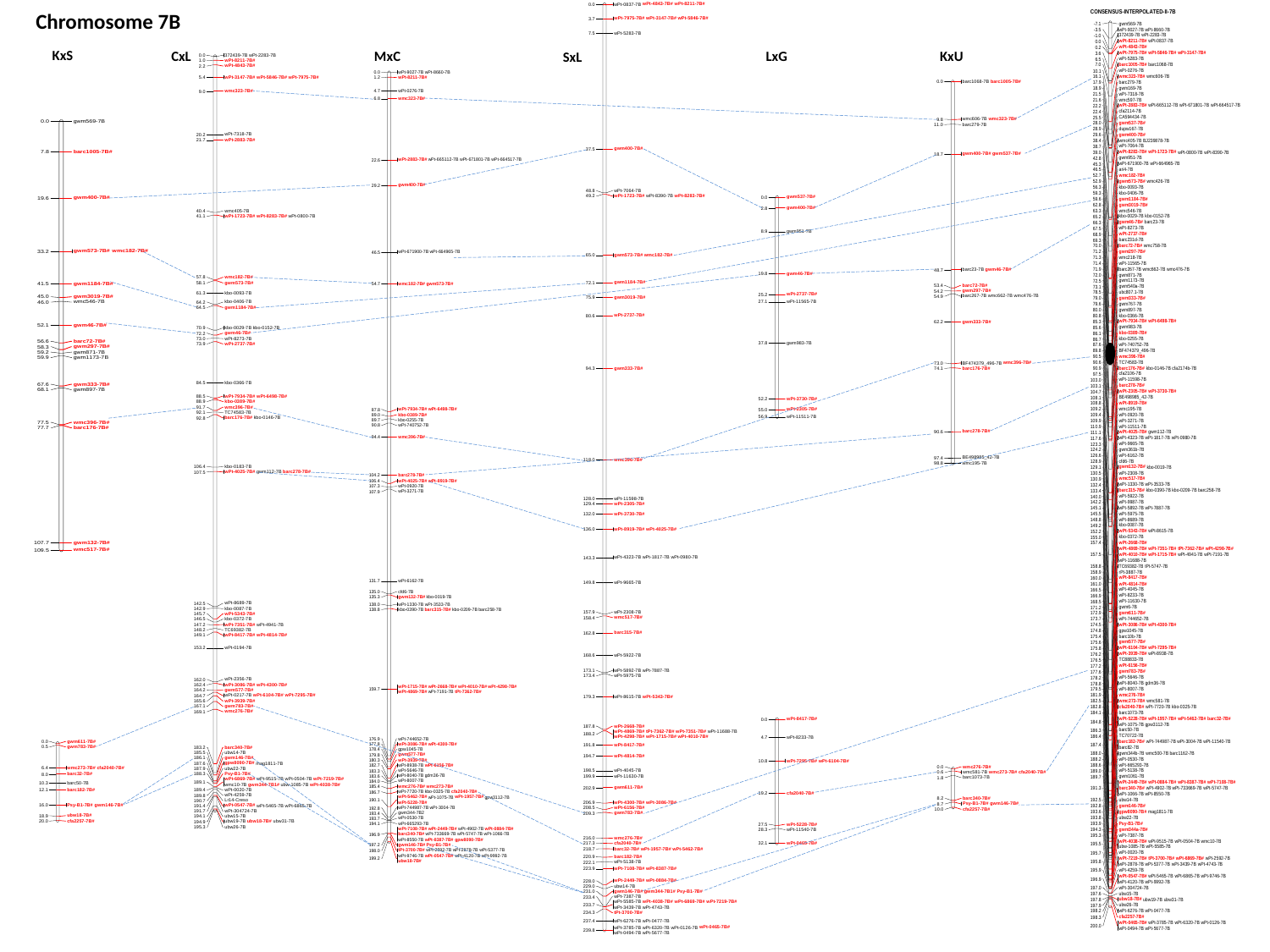

Chromosome 7B
KxS
MxC
CxL
KxU
LxG
SxL

## Slide 16
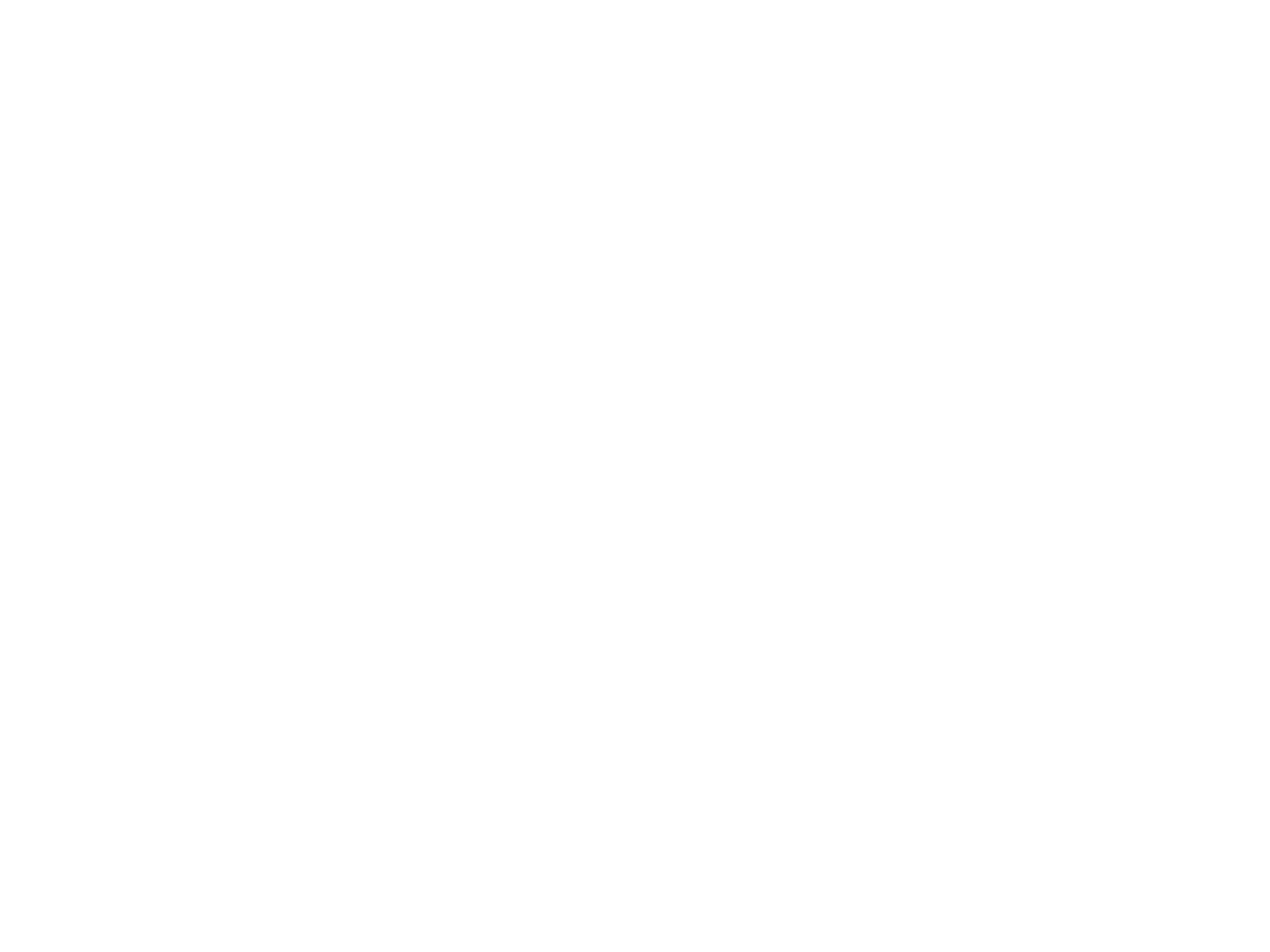

Supplement: Supplementary file 1 — Additional file 1: Figure S1: Representations of the consensus linkage map and of the six core component maps. Graphical representation of the consensus linkage map and of the six core linkage maps used to produce the consensus framework. The consensus linkage map is on the right side of the linkage graphics. Anchor (common) markers are in red font. Markers common to three or more maps are interconnected by dashed lines. (PPTX 3 MB) [file 12864_2014_6782_MOESM1_ESM.pptx]
