# Supplementary material for: A consensus framework map of durum wheat (Triticum durum Desf.) suitable for linkage disequilibrium analysis and genome-wide association mapping
Source: BMC Genomics. 2014 Oct 7;15(1):873. doi: 10.1186/1471-2164-15-873 (PMC4287192; doi:10.1186/1471-2164-15-873)
Supplement: Supplementary file 3 — Additional file 3: Figure S2: Projection plots of the six core component maps on the consensus. The core component linkage group maps are reported in the X axes of the projection plots while the consensus map is reported in the Y axes. Spearman rank correlations are reported as ro (ρ) coefficients. (PPTX 3 MB) [file 12864_2014_6782_MOESM3_ESM.pptx]

## Slide 1
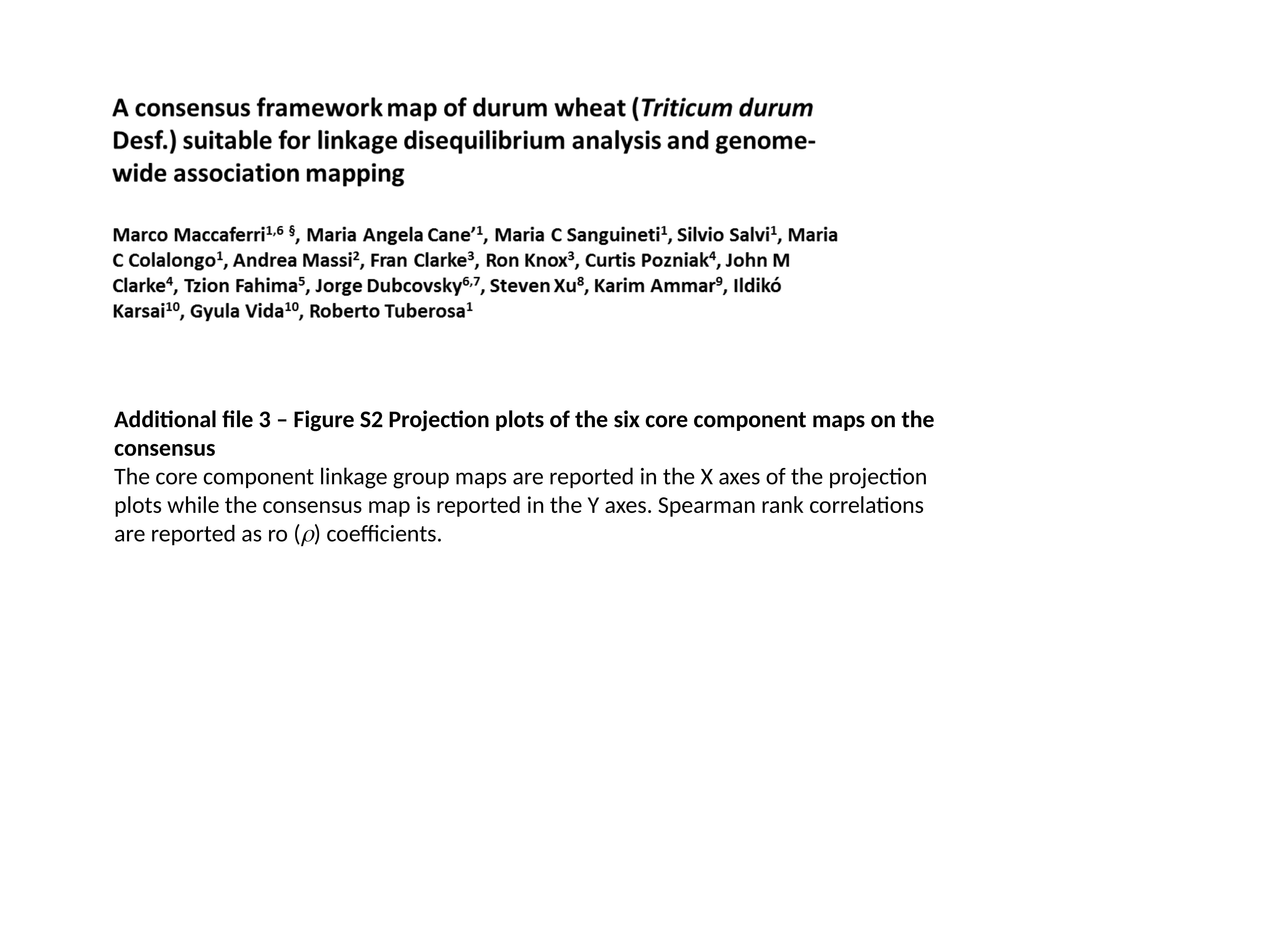

Additional file 3 – Figure S2 Projection plots of the six core component maps on the consensus
The core component linkage group maps are reported in the X axes of the projection plots while the consensus map is reported in the Y axes. Spearman rank correlations are reported as ro (r) coefficients.

## Slide 2
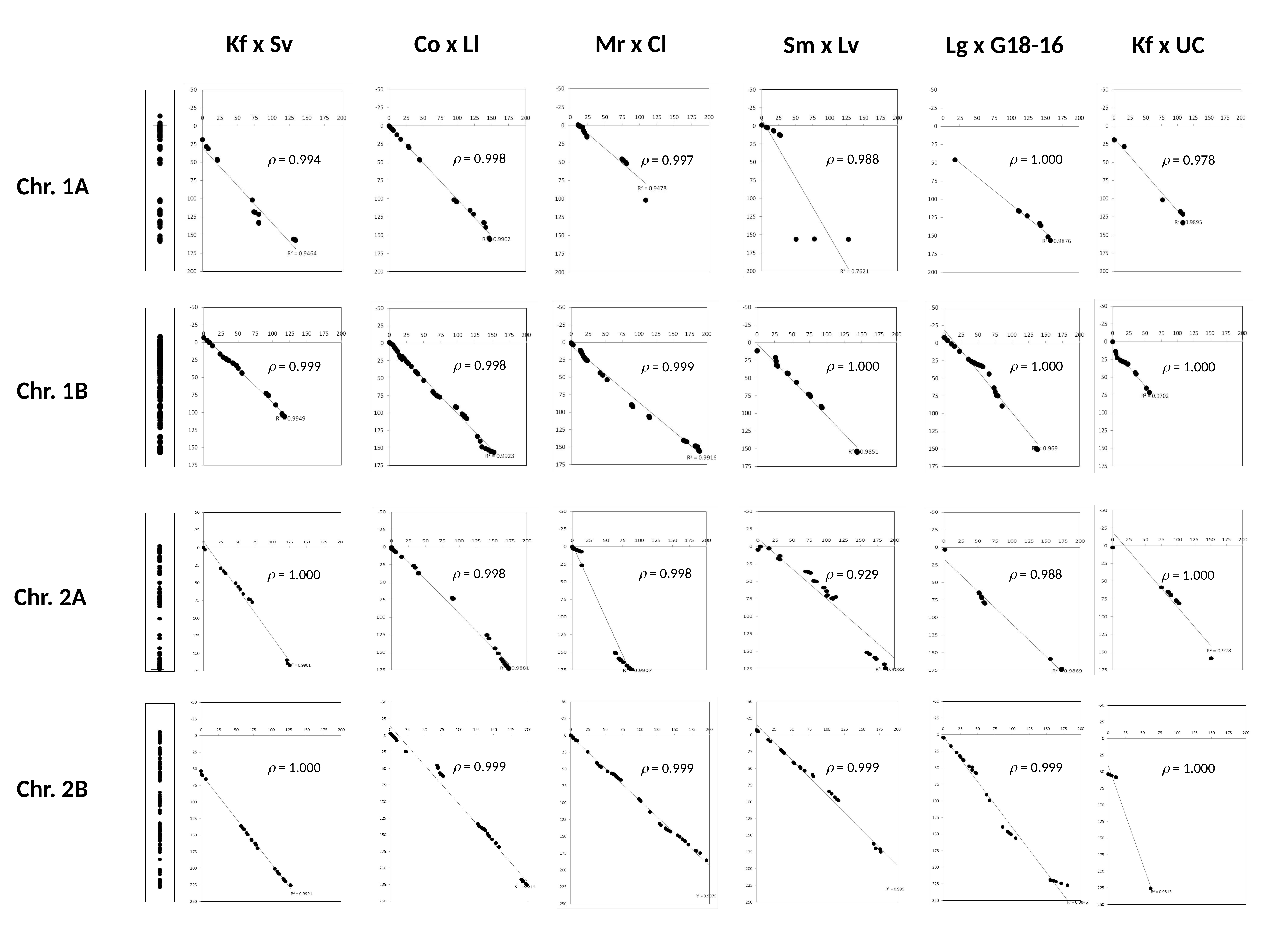

Kf x Sv
Co x Ll
Mr x Cl
Sm x Lv
Lg x G18-16
Kf x UC
r = 0.998
r = 0.988
r = 1.000
r = 0.994
r = 0.997
r = 0.978
Chr. 1A
r = 0.998
r = 1.000
r = 1.000
r = 0.999
r = 0.999
r = 1.000
Chr. 1B
r = 0.998
r = 0.998
r = 0.929
r = 0.988
r = 1.000
r = 1.000
Chr. 2A
r = 0.999
r = 0.999
r = 0.999
r = 1.000
r = 0.999
r = 1.000
Chr. 2B

## Slide 3
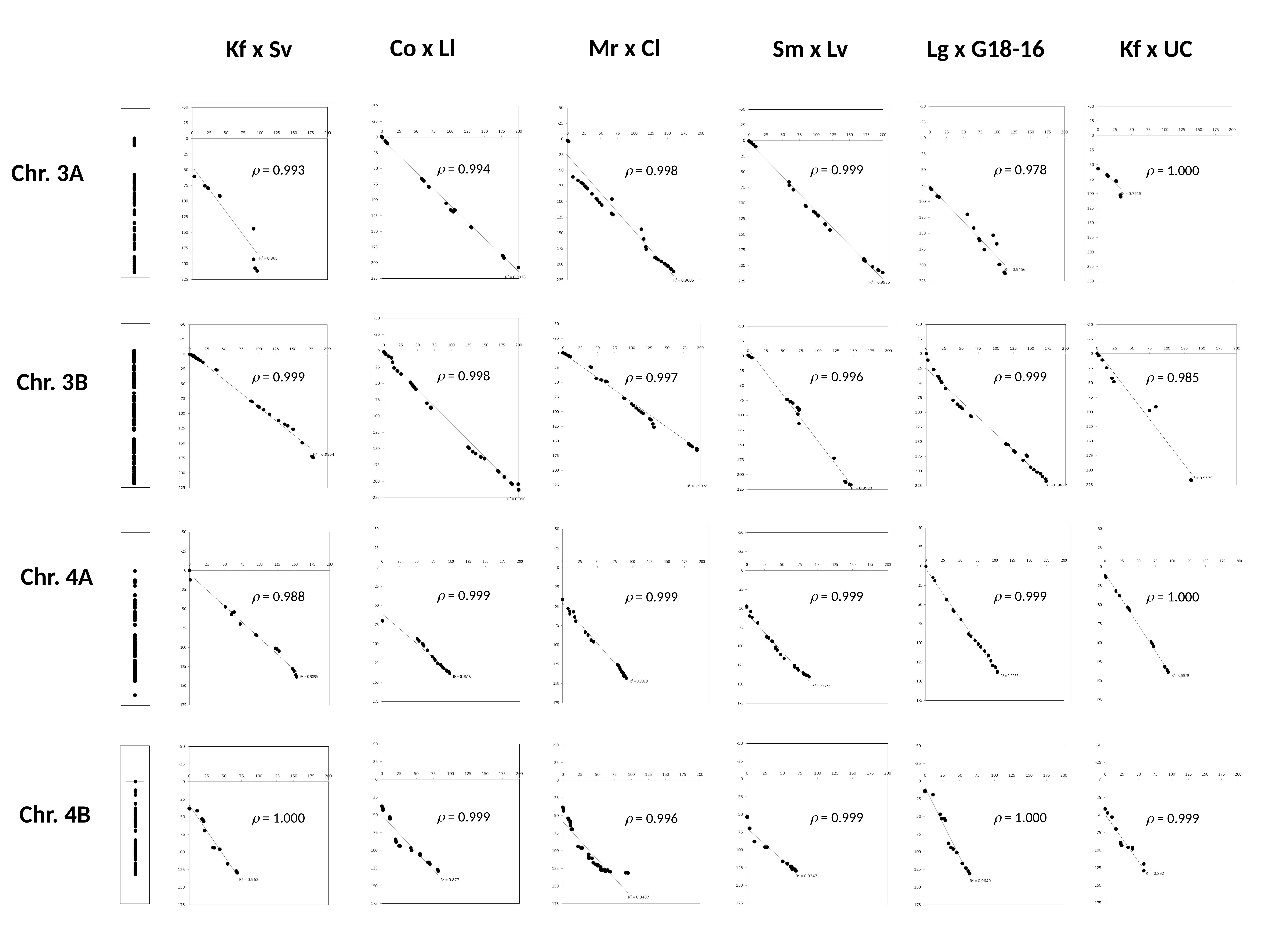

Co x Ll
Mr x Cl
Sm x Lv
Lg x G18-16
Kf x UC
Kf x Sv
Chr. 3A
r = 0.994
r = 0.999
r = 0.978
r = 0.993
r = 0.998
r = 1.000
Chr. 3B
r = 0.998
r = 0.996
r = 0.999
r = 0.999
r = 0.997
r = 0.985
Chr. 4A
r = 0.999
r = 0.999
r = 0.999
r = 0.988
r = 0.999
r = 1.000
Chr. 4B
r = 0.999
r = 0.999
r = 1.000
r = 1.000
r = 0.996
r = 0.999

## Slide 4
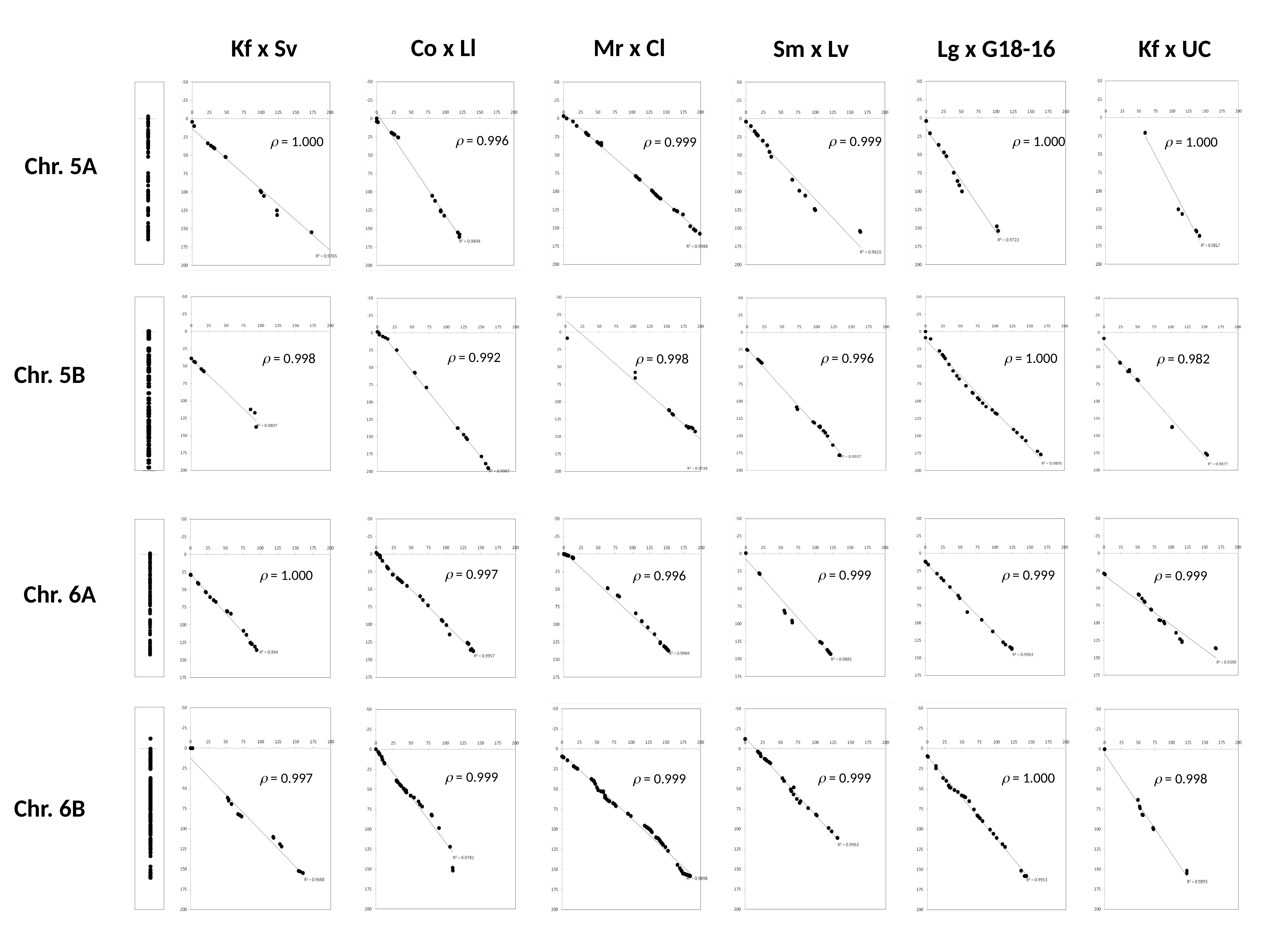

Co x Ll
Mr x Cl
Kf x Sv
Sm x Lv
Lg x G18-16
Kf x UC
r = 0.996
r = 0.999
r = 1.000
r = 1.000
r = 0.999
r = 1.000
Chr. 5A
r = 0.992
r = 0.996
r = 1.000
r = 0.998
r = 0.998
r = 0.982
Chr. 5B
r = 0.997
r = 0.999
r = 0.999
r = 1.000
r = 0.996
r = 0.999
Chr. 6A
r = 0.999
r = 0.999
r = 1.000
r = 0.997
r = 0.999
r = 0.998
Chr. 6B

## Slide 5
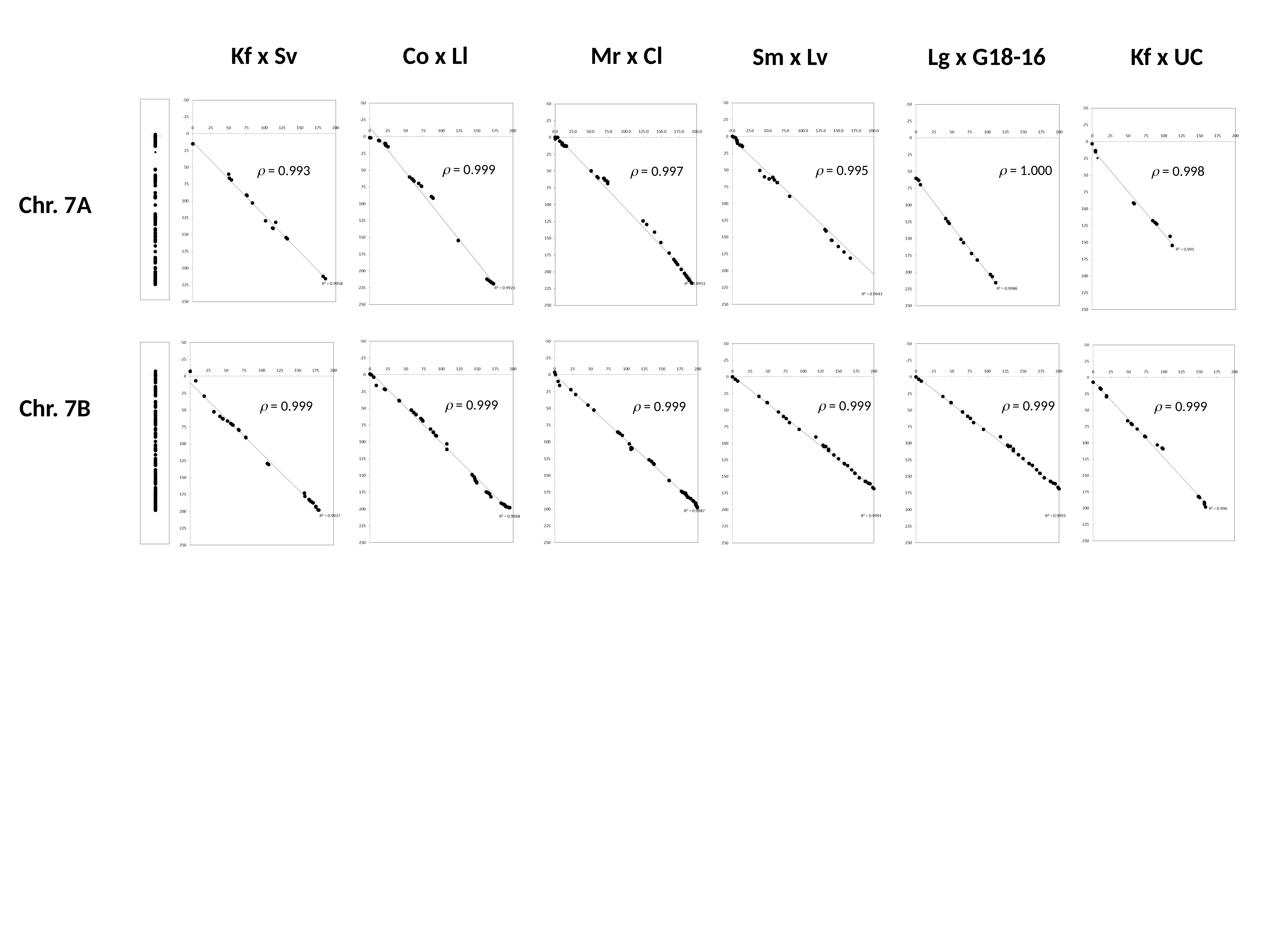

Kf x Sv
Co x Ll
Mr x Cl
Sm x Lv
Lg x G18-16
Kf x UC
r = 0.999
r = 0.995
r = 1.000
r = 0.993
r = 0.997
r = 0.998
Chr. 7A
Chr. 7B
r = 0.999
r = 0.999
r = 0.999
r = 0.999
r = 0.999
r = 0.999
