# Supplementary material for: A consensus framework map of durum wheat (Triticum durum Desf.) suitable for linkage disequilibrium analysis and genome-wide association mapping
Source: BMC Genomics. 2014 Oct 7;15(1):873. doi: 10.1186/1471-2164-15-873 (PMC4287192; doi:10.1186/1471-2164-15-873)
Supplement: Supplementary file 4 — Additional file 4: Figure S3A: Projection plots of the tetraploid wheat consensus map on the hexaploid SSR reference map (Ta-SSR-2004). (PPTX 404 KB) [file 12864_2014_6782_MOESM4_ESM.pptx]

## Slide 1
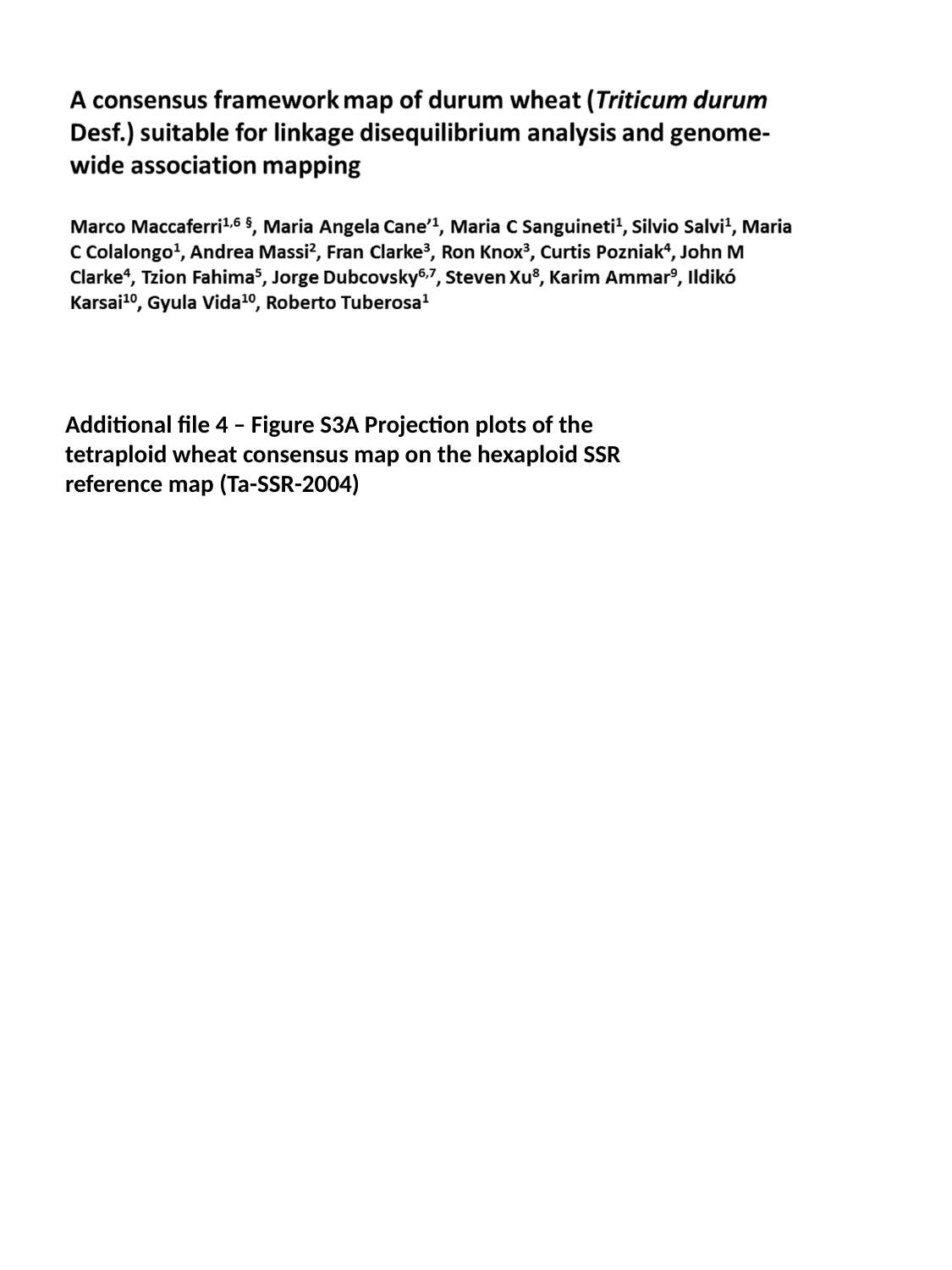

Additional file 4 – Figure S3A Projection plots of the tetraploid wheat consensus map on the hexaploid SSR reference map (Ta-SSR-2004)

## Slide 2
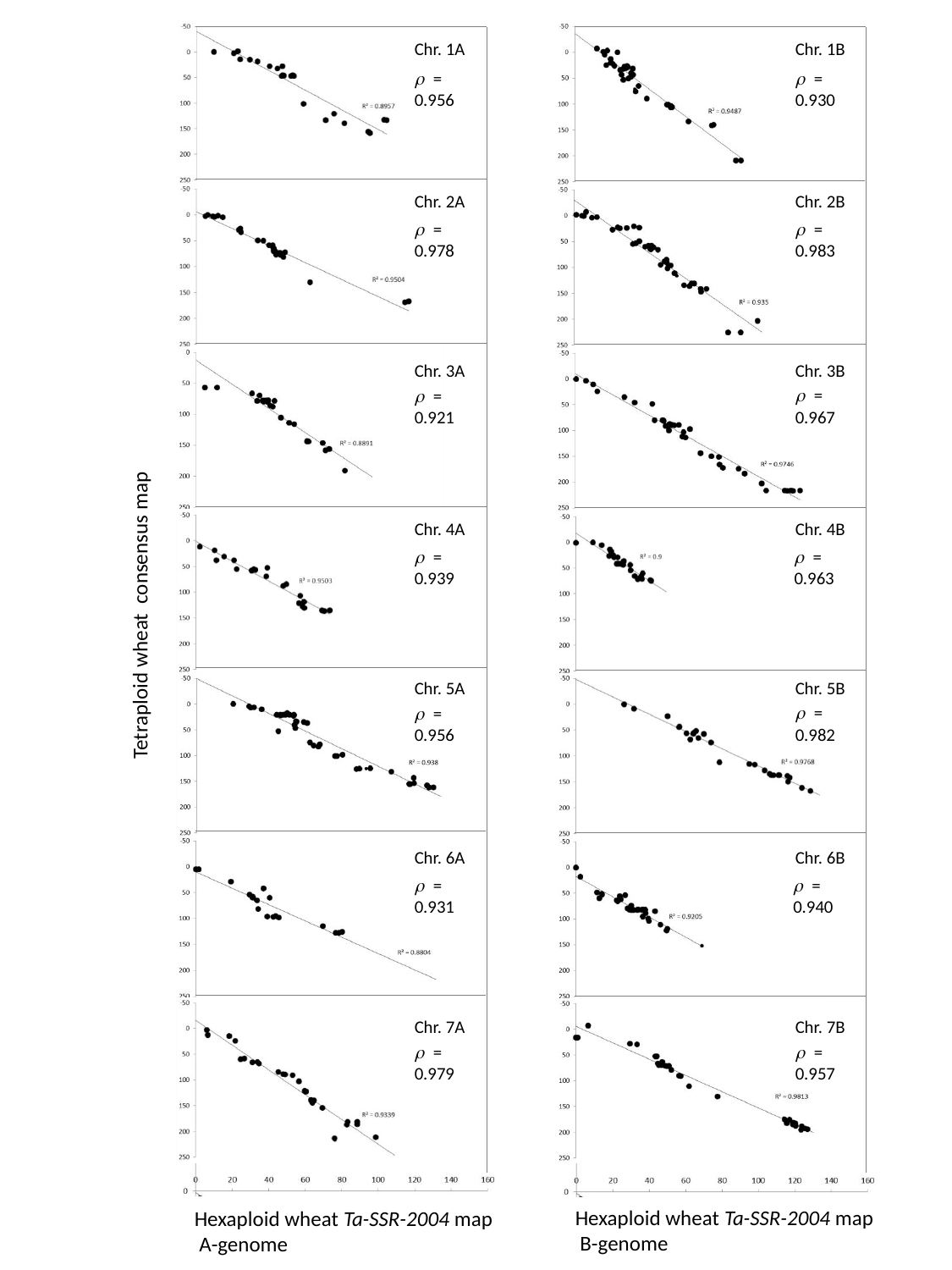

Hexaploid wheat Ta-SSR-2004 map B-genome
Chr. 1A
Chr. 1B
r = 0.956
r = 0.930
Chr. 2A
Chr. 2B
r = 0.978
r = 0.983
Chr. 3A
Chr. 3B
r = 0.967
r = 0.921
Chr. 4A
Chr. 4B
r = 0.939
r = 0.963
Tetraploid wheat consensus map
Chr. 5A
Chr. 5B
r = 0.982
r = 0.956
Chr. 6A
Chr. 6B
r = 0.931
r = 0.940
Chr. 7A
Chr. 7B
r = 0.957
r = 0.979
Hexaploid wheat Ta-SSR-2004 map A-genome
