# Supplementary material for: A consensus framework map of durum wheat (Triticum durum Desf.) suitable for linkage disequilibrium analysis and genome-wide association mapping
Source: BMC Genomics. 2014 Oct 7;15(1):873. doi: 10.1186/1471-2164-15-873 (PMC4287192; doi:10.1186/1471-2164-15-873)
Supplement: Supplementary file 5 — Additional file 5: Figure S3B: Projection plots of the tetraploid wheat consensus map on the tetraploid mappreviously reported by Marone et al. [19]. (PPTX 149 KB) [file 12864_2014_6782_MOESM5_ESM.pptx]

## Slide 1
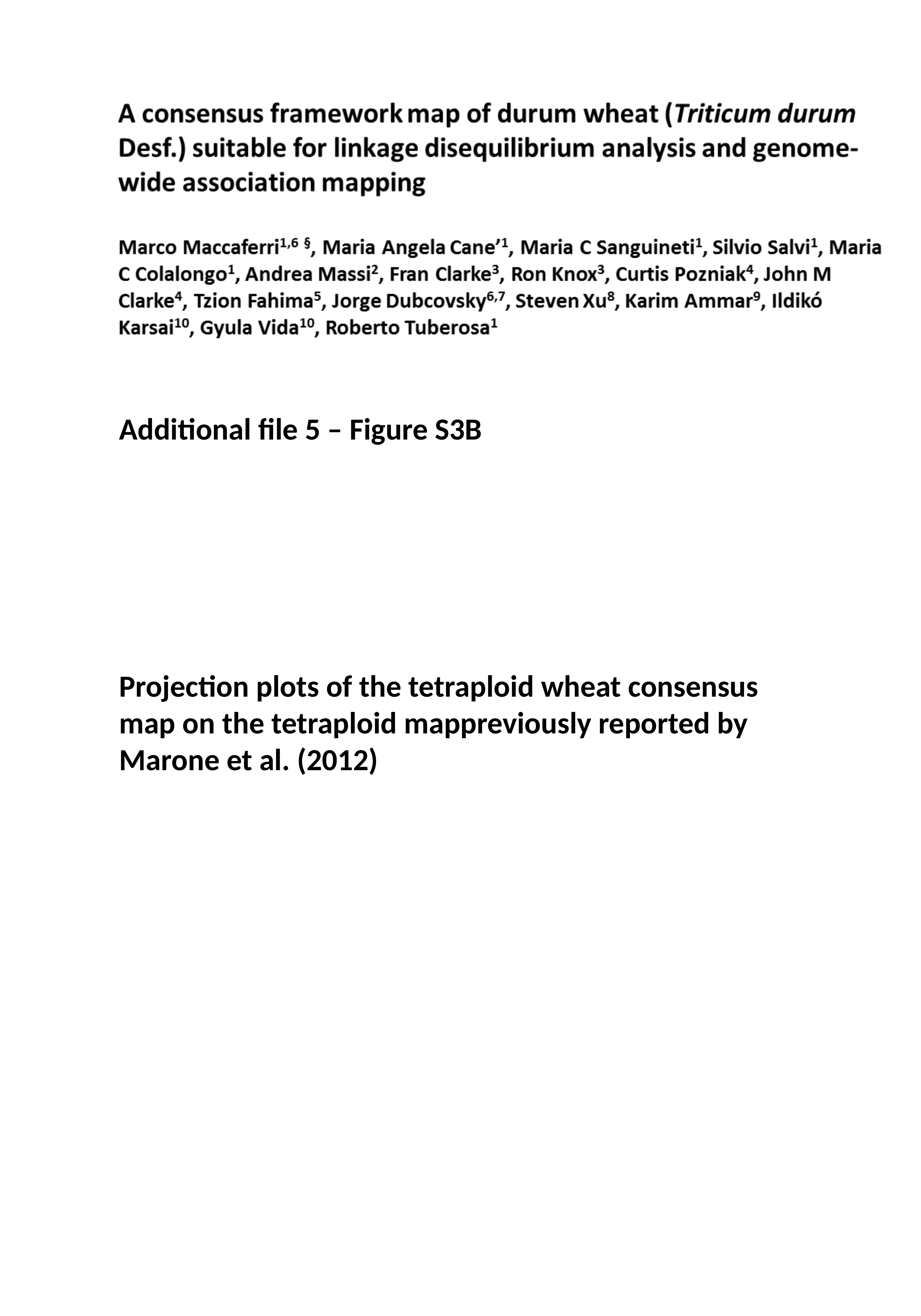

Additional file 5 – Figure S3B Projection plots of the tetraploid wheat consensus map on the tetraploid mappreviously reported by Marone et al. (2012)

## Slide 2
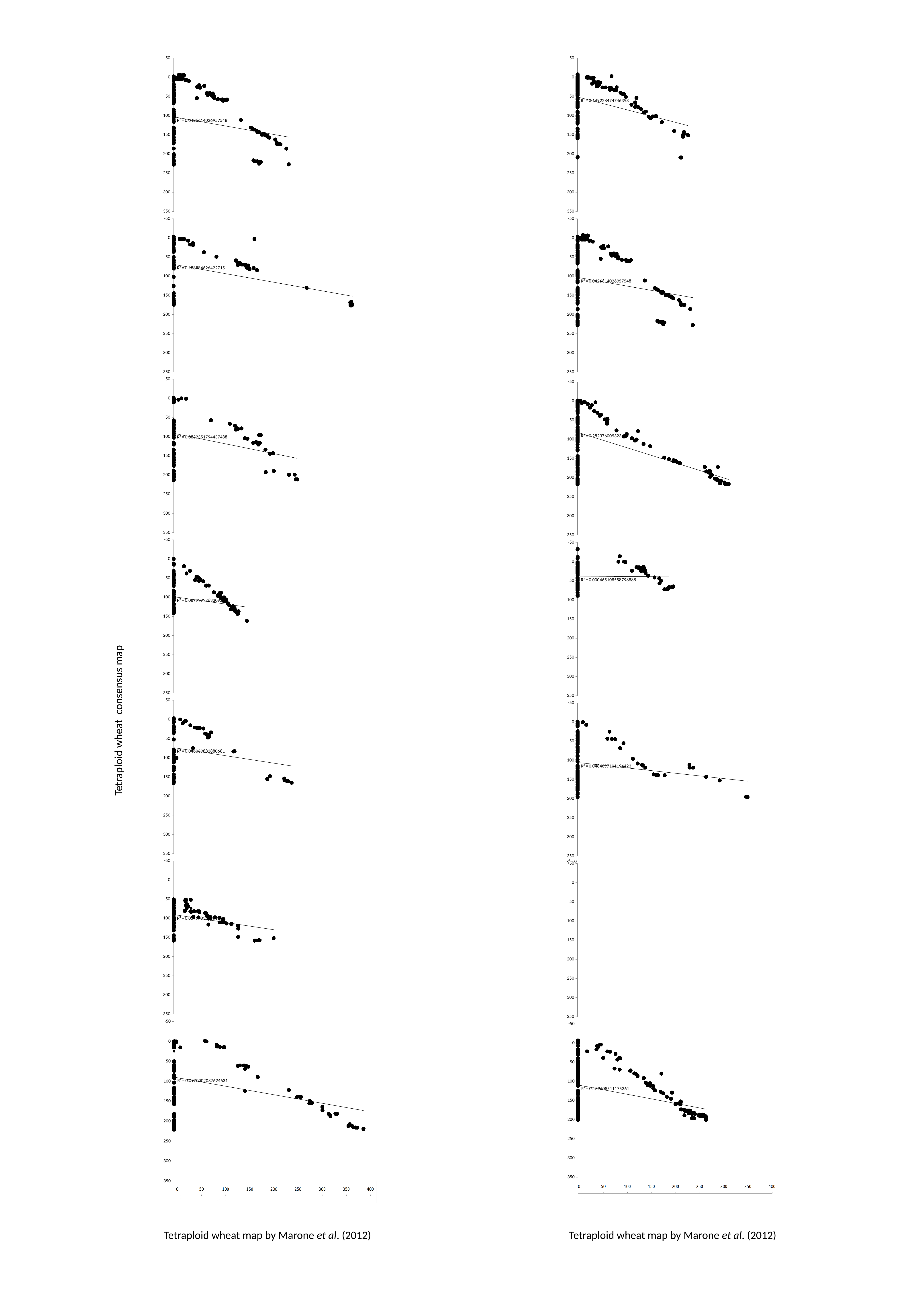

### Chart
| Category | |
|---|---|
### Chart
| Category | |
|---|---|
### Chart
| Category | |
|---|---|
### Chart
| Category | |
|---|---|
### Chart
| Category | |
|---|---|
### Chart
| Category | |
|---|---|
### Chart
| Category | |
|---|---|
### Chart
| Category | |
|---|---|
### Chart
| Category | |
|---|---|Tetraploid wheat consensus map
### Chart
| Category | |
|---|---|
### Chart
| Category | |
|---|---|
### Chart
| Category | |
|---|---|
### Chart
| Category | |
|---|---|
### Chart
| Category | |
|---|---|
Tetraploid wheat map by Marone et al. (2012)
Tetraploid wheat map by Marone et al. (2012)
