# Supplementary material for: A consensus framework map of durum wheat (Triticum durum Desf.) suitable for linkage disequilibrium analysis and genome-wide association mapping
Source: BMC Genomics. 2014 Oct 7;15(1):873. doi: 10.1186/1471-2164-15-873 (PMC4287192; doi:10.1186/1471-2164-15-873)
Supplement: Supplementary file 6 — Additional file 6: Figure S4: Detailed pairwise linkage disequilibrium plots, distribution of loci with highly significant Fst differentiation patterns and of the polymorphism index content values of SSR and DArT® markers along the tetraploid wheat consensus map, as estimated from the elite durum wheat germplasm accessions from world-wide. Pairwise linkage disequilibrium (LD) patterns are shown for the squared coefficients of determination (r 2) values between loci used to profile a panel of 183 elite durum accessions and mapped on the consensus map (left side of chromosome bars). Loci with highly significant FST differentiation values between durum sub-population groups are reported on the right side of the chromosome bars, as long with the locus polymorphism index content (PIC) plots, normalized for allele number. (PPTX 7 MB) [file 12864_2014_6782_MOESM6_ESM.pptx]

## Slide 1
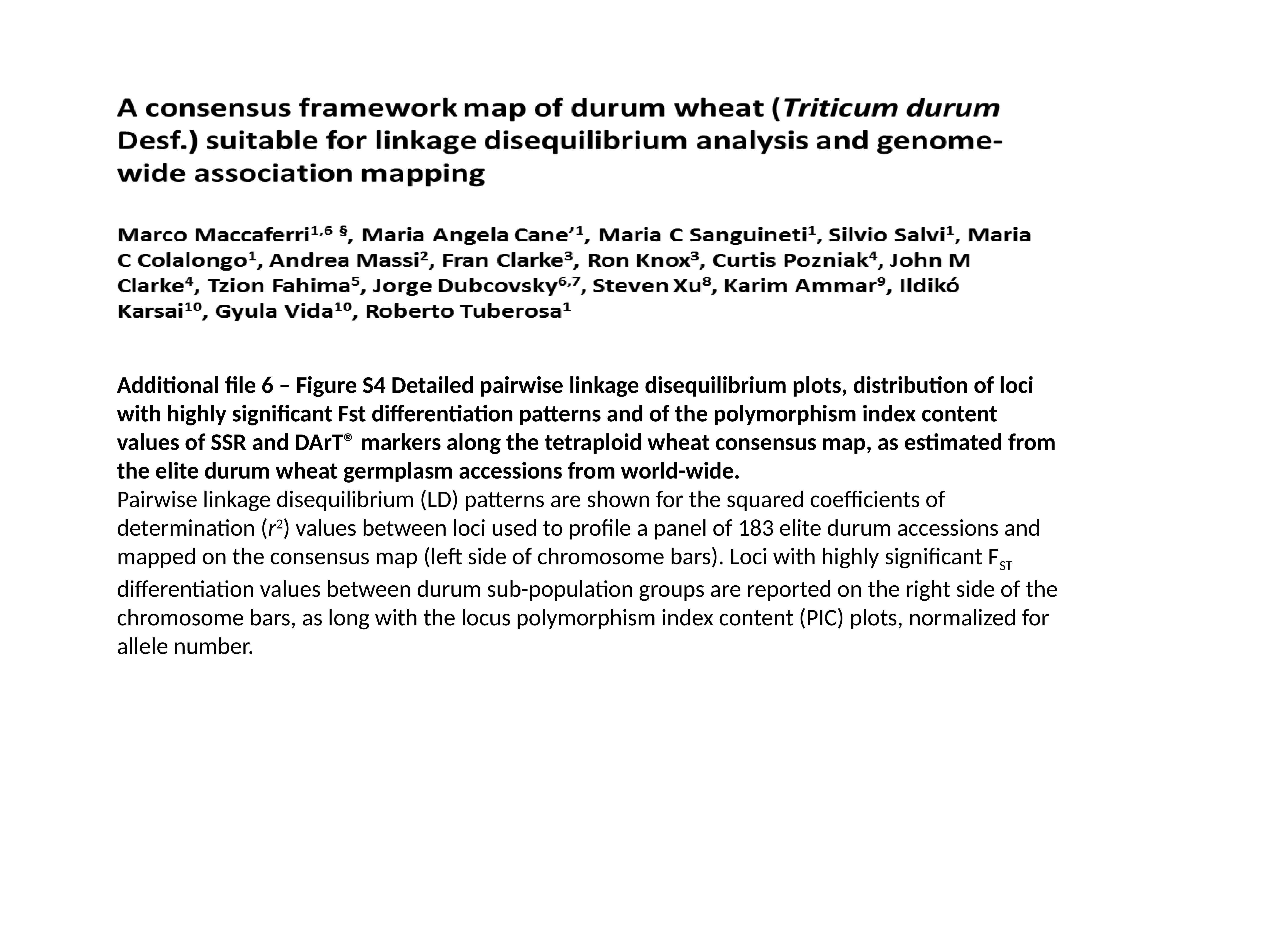

Additional file 6 – Figure S4 Detailed pairwise linkage disequilibrium plots, distribution of loci with highly significant Fst differentiation patterns and of the polymorphism index content values of SSR and DArT® markers along the tetraploid wheat consensus map, as estimated from the elite durum wheat germplasm accessions from world-wide.
Pairwise linkage disequilibrium (LD) patterns are shown for the squared coefficients of determination (r2) values between loci used to profile a panel of 183 elite durum accessions and mapped on the consensus map (left side of chromosome bars). Loci with highly significant FST differentiation values between durum sub-population groups are reported on the right side of the chromosome bars, as long with the locus polymorphism index content (PIC) plots, normalized for allele number.

## Slide 2
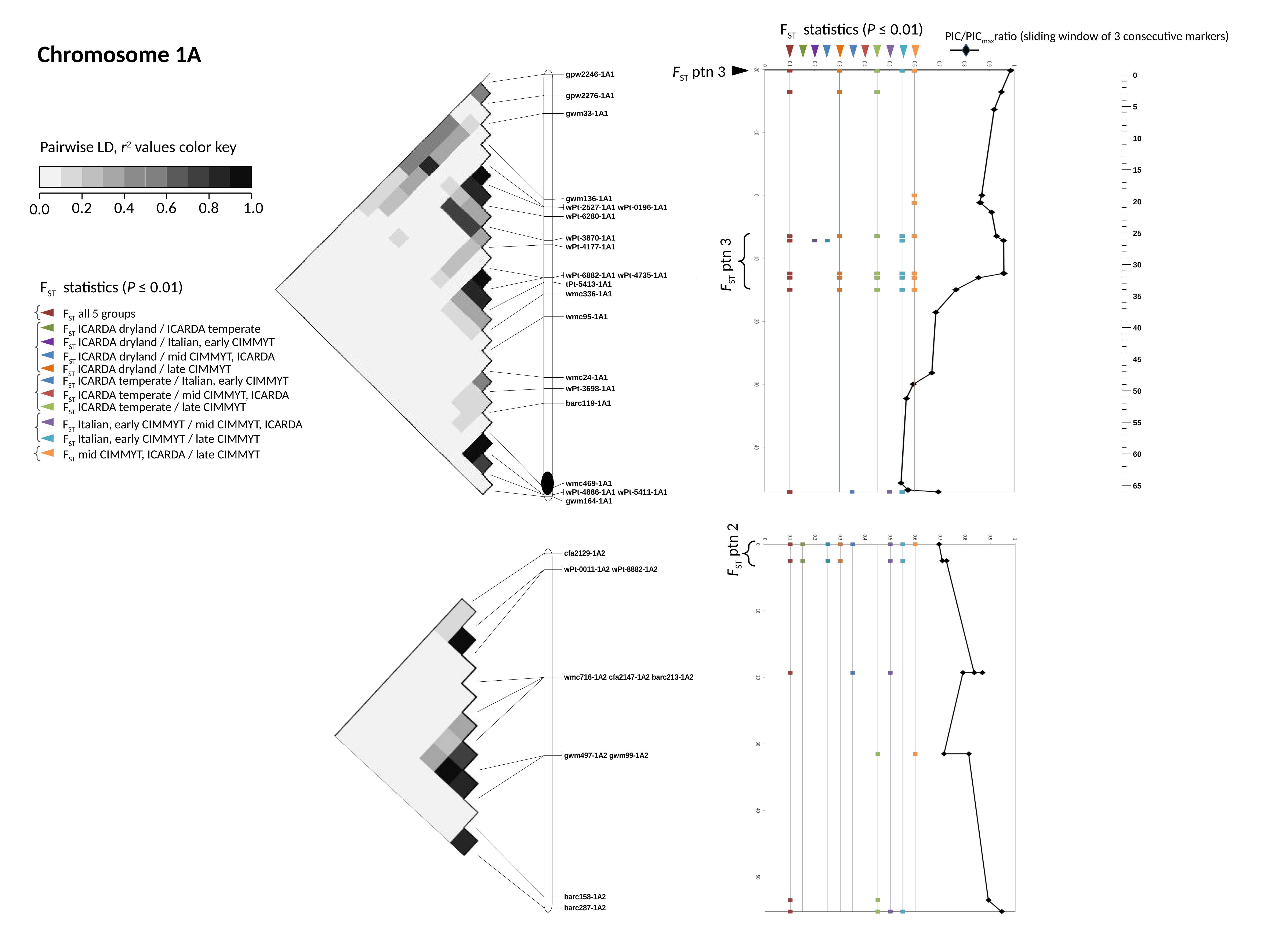

FST statistics (P ≤ 0.01)
PIC/PICmaxratio (sliding window of 3 consecutive markers)
Chromosome 1A
FST ptn 3
Pairwise LD, r2 values color key
0.2
0.4
0.6
0.8
1.0
0.0
FST ptn 3
FST statistics (P ≤ 0.01)
FST all 5 groups
FST ICARDA dryland / ICARDA temperate
FST ICARDA dryland / Italian, early CIMMYT
FST ICARDA dryland / mid CIMMYT, ICARDA
FST ICARDA dryland / late CIMMYT
FST ICARDA temperate / Italian, early CIMMYT
FST ICARDA temperate / mid CIMMYT, ICARDA
FST ICARDA temperate / late CIMMYT
FST Italian, early CIMMYT / mid CIMMYT, ICARDA
FST Italian, early CIMMYT / late CIMMYT
FST mid CIMMYT, ICARDA / late CIMMYT
FST ptn 2

## Slide 3
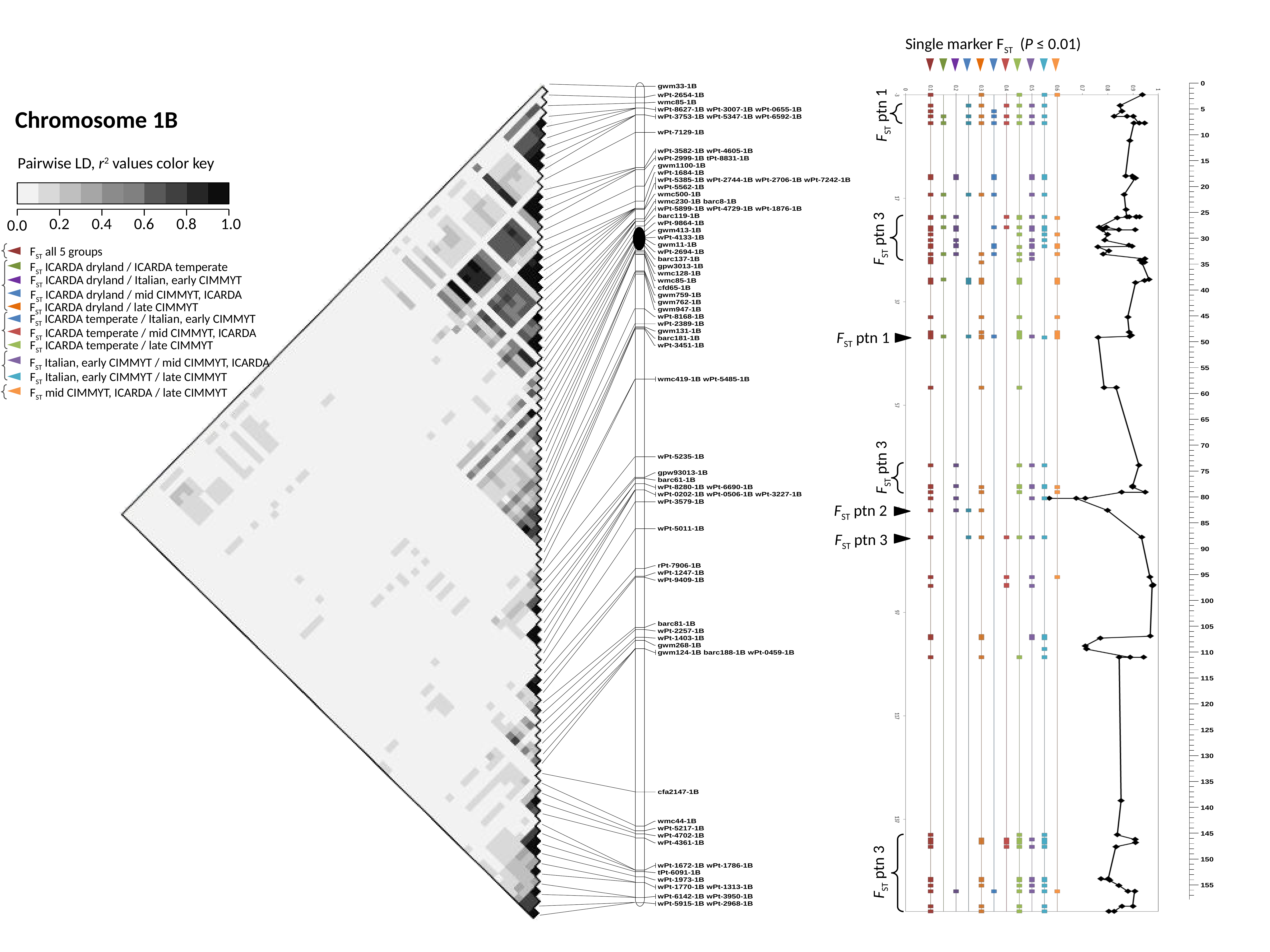

Single marker FST (P ≤ 0.01)
FST ptn 1
Chromosome 1B
Pairwise LD, r2 values color key
0.2
0.4
0.6
0.8
1.0
0.0
FST ptn 3
FST all 5 groups
FST ICARDA dryland / ICARDA temperate
FST ICARDA dryland / Italian, early CIMMYT
FST ICARDA dryland / mid CIMMYT, ICARDA
FST ICARDA dryland / late CIMMYT
FST ICARDA temperate / Italian, early CIMMYT
FST ICARDA temperate / mid CIMMYT, ICARDA
FST ptn 1
FST ICARDA temperate / late CIMMYT
FST Italian, early CIMMYT / mid CIMMYT, ICARDA
FST Italian, early CIMMYT / late CIMMYT
FST mid CIMMYT, ICARDA / late CIMMYT
FST ptn 3
FST ptn 2
FST ptn 3
FST ptn 3

## Slide 4
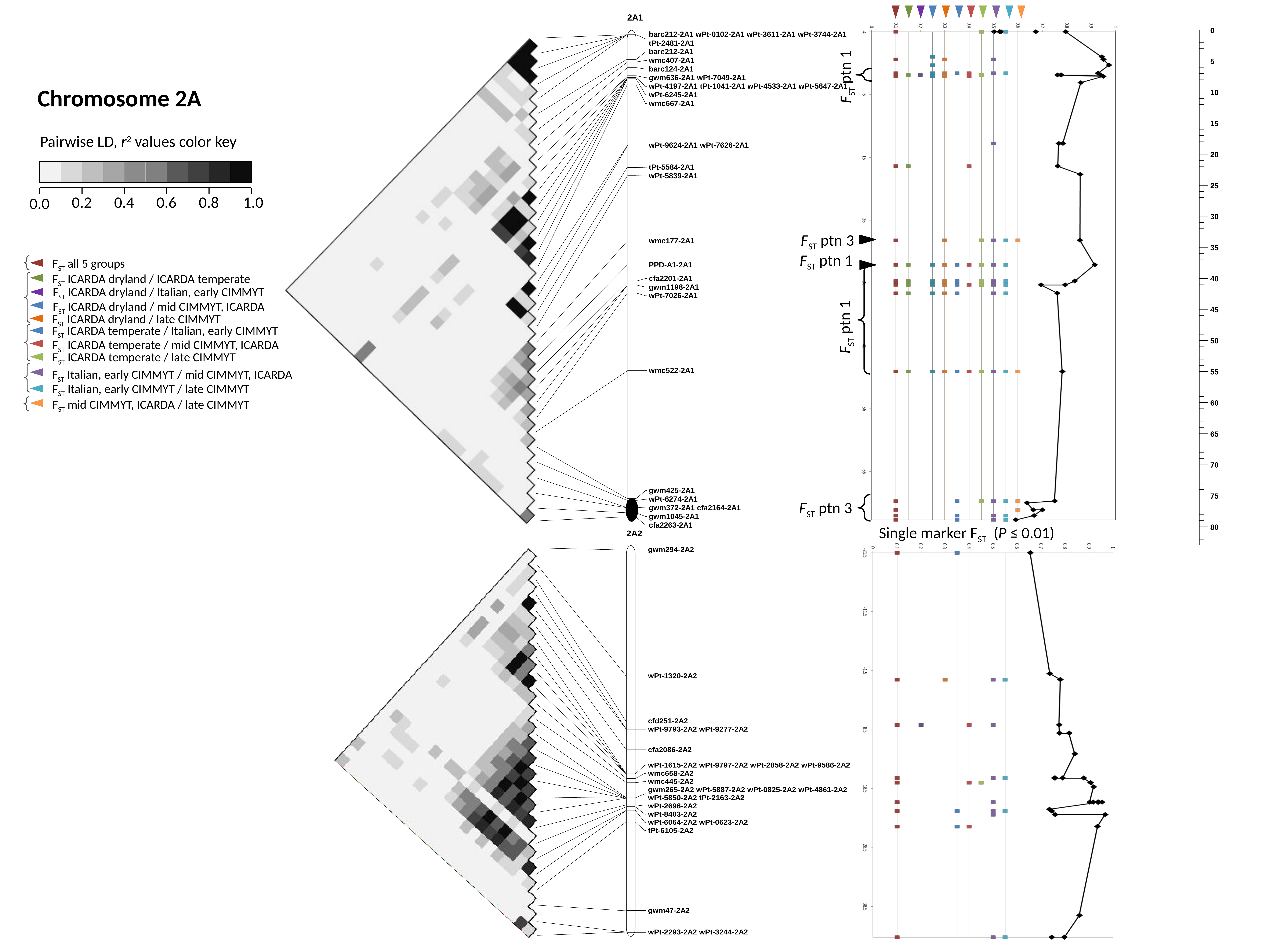

FST ptn 1
Chromosome 2A
Pairwise LD, r2 values color key
0.2
0.4
0.6
0.8
1.0
0.0
FST ptn 3
FST ptn 1
FST all 5 groups
FST ICARDA dryland / ICARDA temperate
FST ICARDA dryland / Italian, early CIMMYT
FST ptn 1
FST ICARDA dryland / mid CIMMYT, ICARDA
FST ICARDA dryland / late CIMMYT
FST ICARDA temperate / Italian, early CIMMYT
FST ICARDA temperate / mid CIMMYT, ICARDA
FST ICARDA temperate / late CIMMYT
FST Italian, early CIMMYT / mid CIMMYT, ICARDA
FST Italian, early CIMMYT / late CIMMYT
FST mid CIMMYT, ICARDA / late CIMMYT
FST ptn 3
Single marker FST (P ≤ 0.01)

## Slide 5
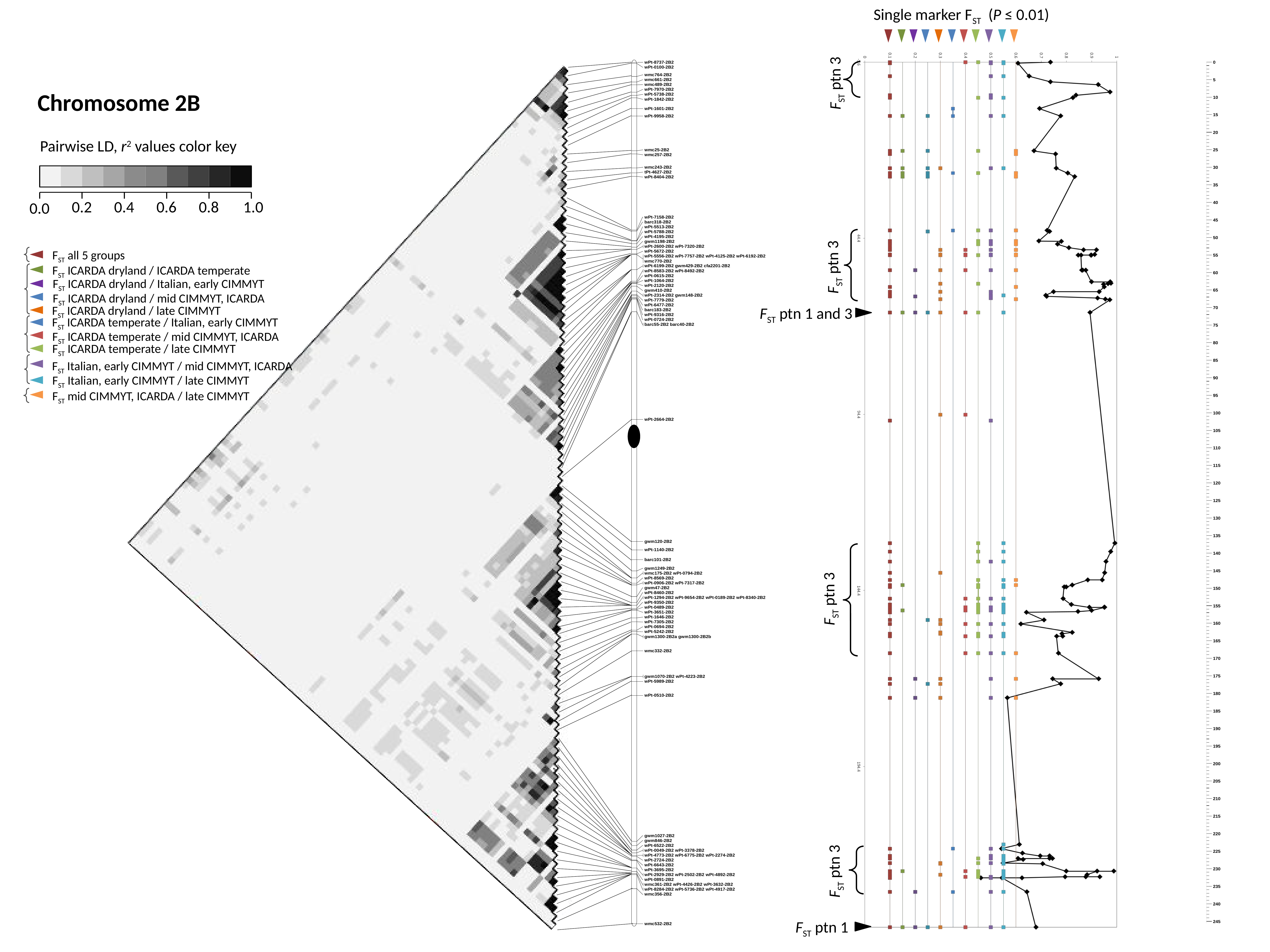

Single marker FST (P ≤ 0.01)
FST ptn 3
Chromosome 2B
Pairwise LD, r2 values color key
0.2
0.4
0.6
0.8
1.0
0.0
FST all 5 groups
FST ptn 3
FST ICARDA dryland / ICARDA temperate
FST ICARDA dryland / Italian, early CIMMYT
FST ICARDA dryland / mid CIMMYT, ICARDA
FST ICARDA dryland / late CIMMYT
FST ptn 1 and 3
FST ICARDA temperate / Italian, early CIMMYT
FST ICARDA temperate / mid CIMMYT, ICARDA
FST ICARDA temperate / late CIMMYT
FST Italian, early CIMMYT / mid CIMMYT, ICARDA
FST Italian, early CIMMYT / late CIMMYT
FST mid CIMMYT, ICARDA / late CIMMYT
FST ptn 3
FST ptn 3
FST ptn 1

## Slide 6
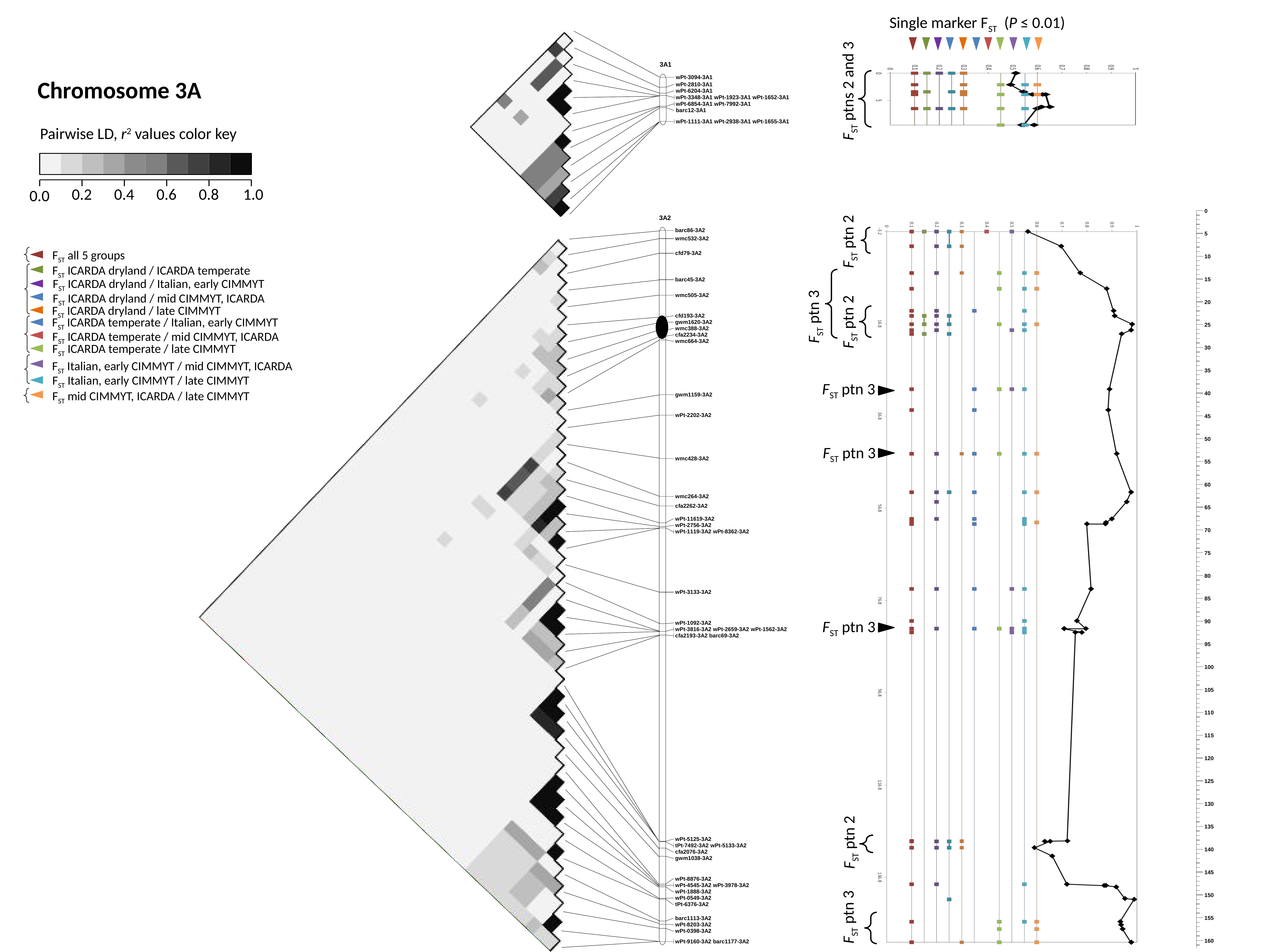

Single marker FST (P ≤ 0.01)
Chromosome 3A
FST ptns 2 and 3
Pairwise LD, r2 values color key
0.2
0.4
0.6
0.8
1.0
0.0
FST ptn 2
FST all 5 groups
FST ICARDA dryland / ICARDA temperate
FST ICARDA dryland / Italian, early CIMMYT
FST ICARDA dryland / mid CIMMYT, ICARDA
FST ICARDA dryland / late CIMMYT
FST ptn 3
FST ptn 2
FST ICARDA temperate / Italian, early CIMMYT
FST ICARDA temperate / mid CIMMYT, ICARDA
FST ICARDA temperate / late CIMMYT
FST Italian, early CIMMYT / mid CIMMYT, ICARDA
FST Italian, early CIMMYT / late CIMMYT
FST ptn 3
FST mid CIMMYT, ICARDA / late CIMMYT
FST ptn 3
FST ptn 3
FST ptn 2
FST ptn 3

## Slide 7
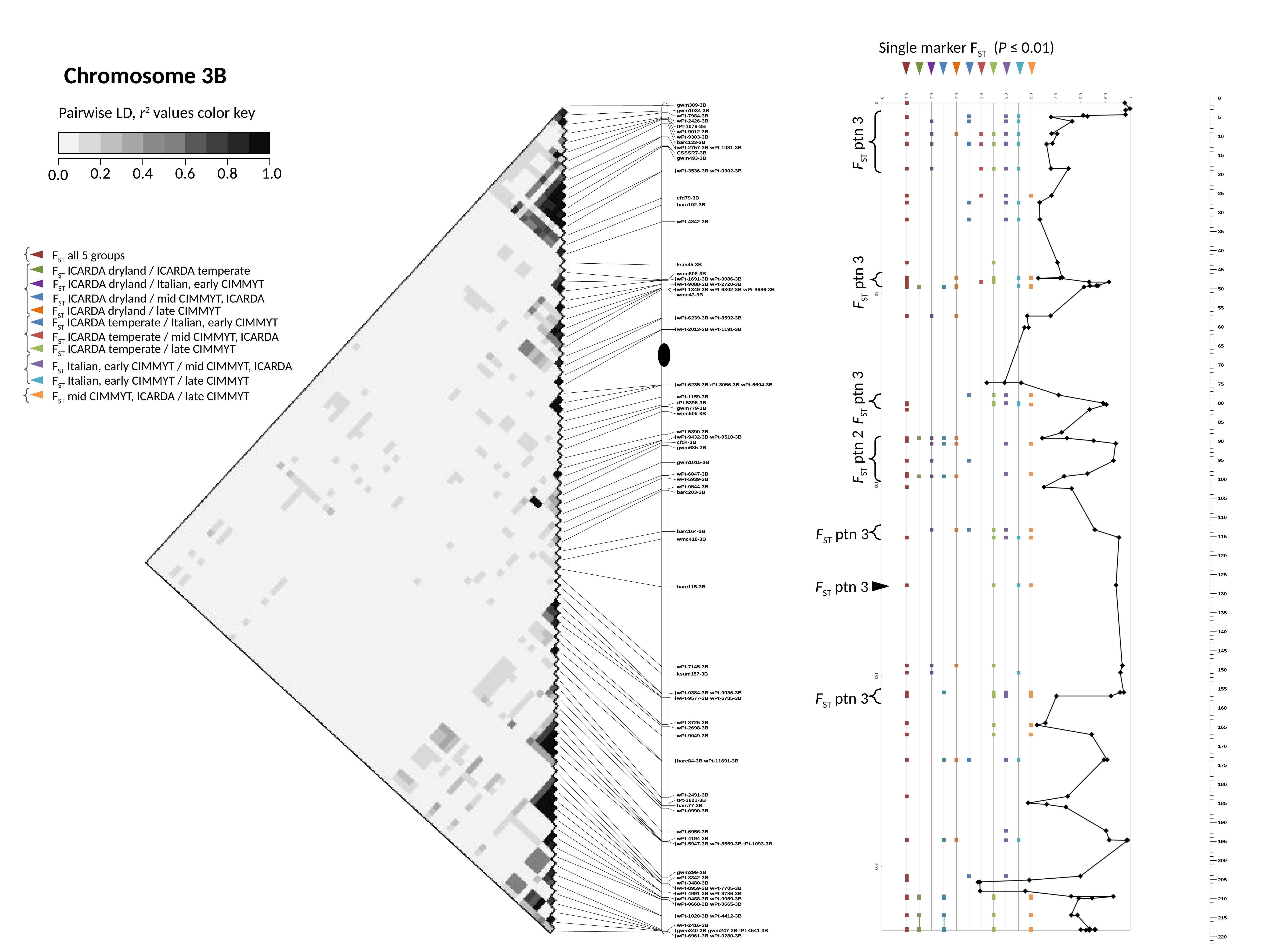

Single marker FST (P ≤ 0.01)
Chromosome 3B
Pairwise LD, r2 values color key
0.2
0.4
0.6
0.8
1.0
0.0
FST ptn 3
FST all 5 groups
FST ICARDA dryland / ICARDA temperate
FST ptn 3
FST ICARDA dryland / Italian, early CIMMYT
FST ICARDA dryland / mid CIMMYT, ICARDA
FST ICARDA dryland / late CIMMYT
FST ICARDA temperate / Italian, early CIMMYT
FST ICARDA temperate / mid CIMMYT, ICARDA
FST ICARDA temperate / late CIMMYT
FST Italian, early CIMMYT / mid CIMMYT, ICARDA
FST Italian, early CIMMYT / late CIMMYT
FST ptn 3
FST mid CIMMYT, ICARDA / late CIMMYT
FST ptn 2
FST ptn 3
FST ptn 3
FST ptn 3

## Slide 8
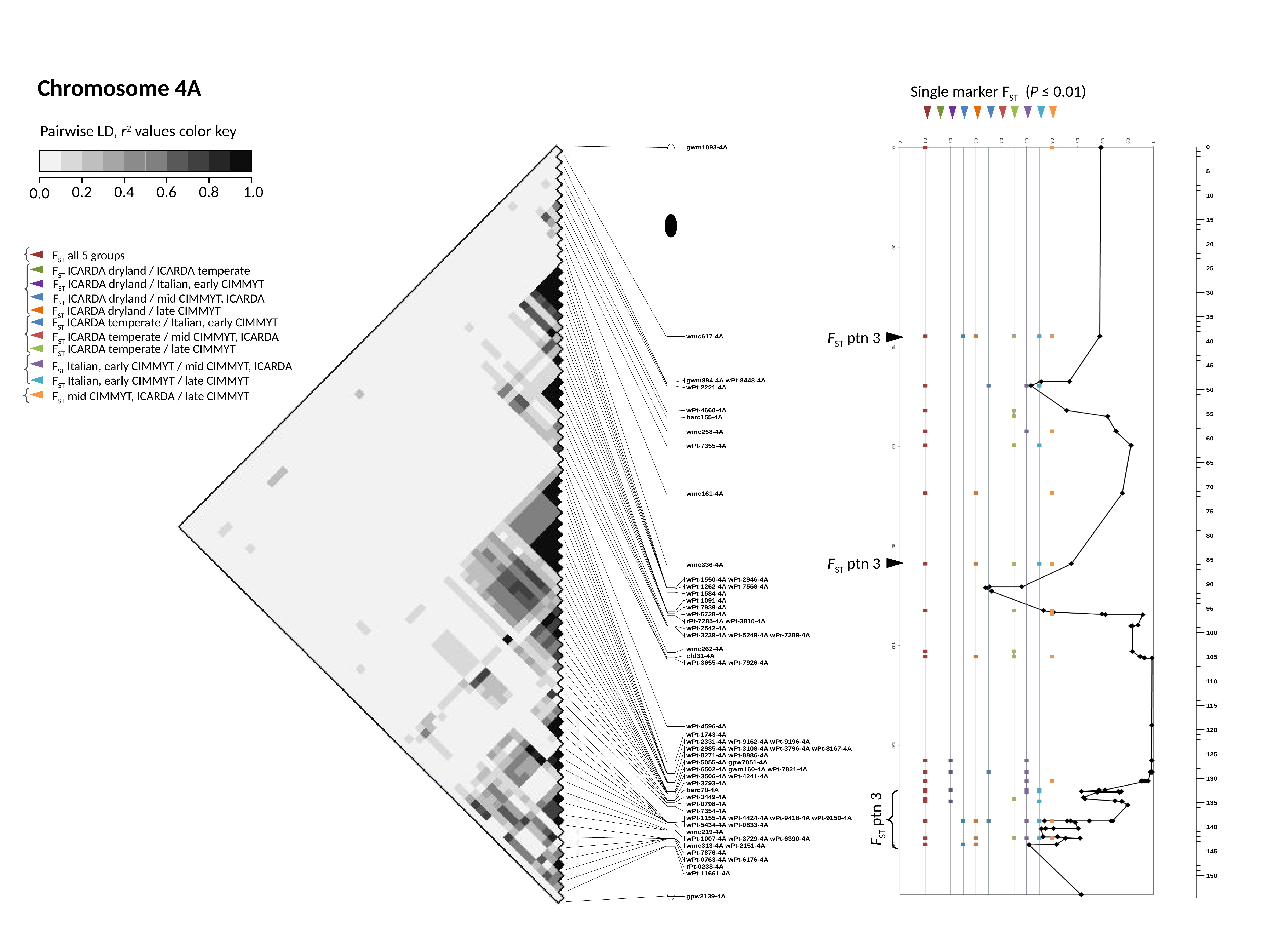

Chromosome 4A
Single marker FST (P ≤ 0.01)
Pairwise LD, r2 values color key
0.2
0.4
0.6
0.8
1.0
0.0
FST all 5 groups
FST ICARDA dryland / ICARDA temperate
FST ICARDA dryland / Italian, early CIMMYT
FST ICARDA dryland / mid CIMMYT, ICARDA
FST ICARDA dryland / late CIMMYT
FST ICARDA temperate / Italian, early CIMMYT
FST ptn 3
FST ICARDA temperate / mid CIMMYT, ICARDA
FST ICARDA temperate / late CIMMYT
FST Italian, early CIMMYT / mid CIMMYT, ICARDA
FST Italian, early CIMMYT / late CIMMYT
FST mid CIMMYT, ICARDA / late CIMMYT
FST ptn 3
FST ptn 3

## Slide 9
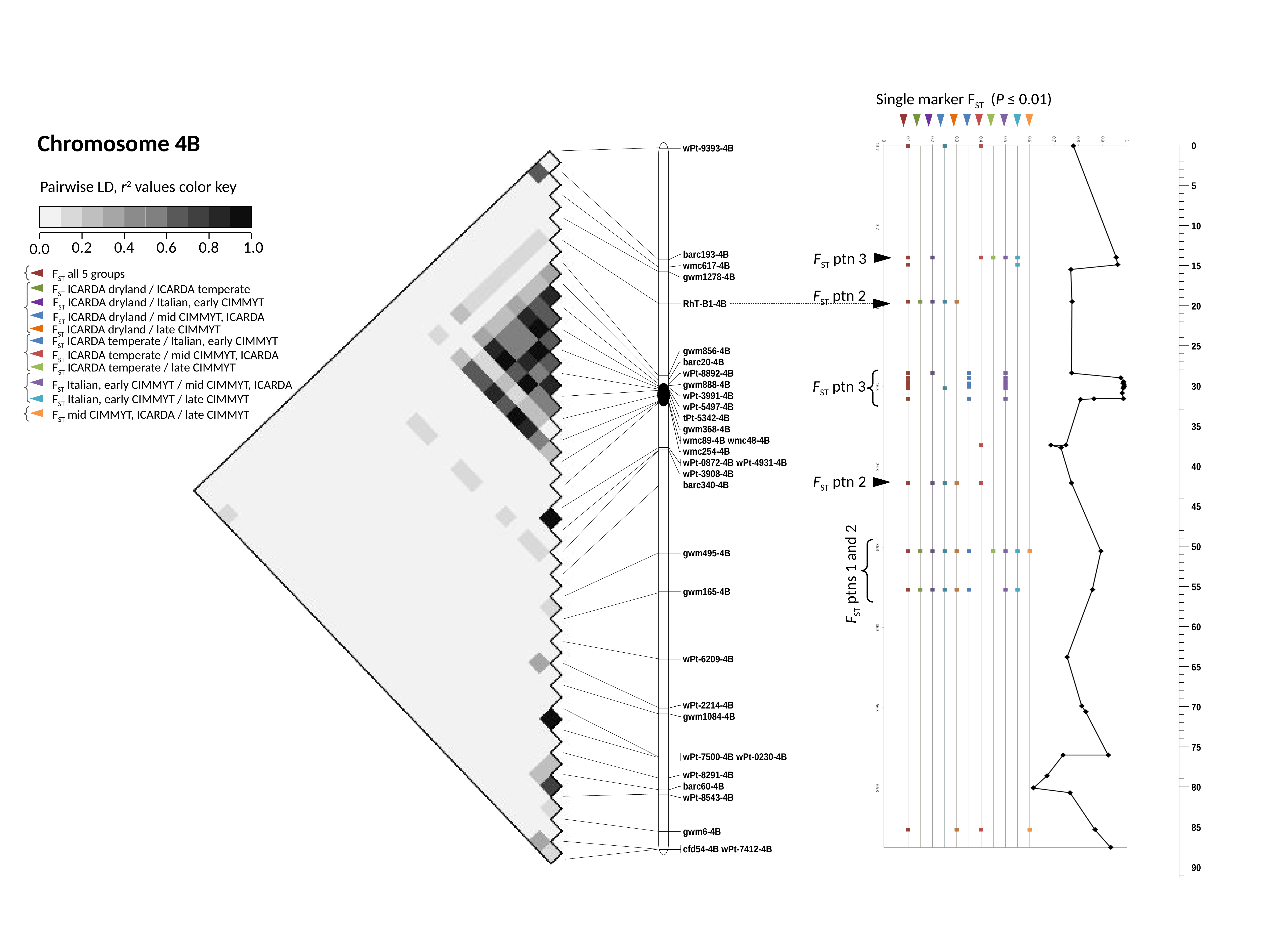

Single marker FST (P ≤ 0.01)
Chromosome 4B
Pairwise LD, r2 values color key
0.2
0.4
0.6
0.8
1.0
0.0
FST ptn 3
FST all 5 groups
FST ICARDA dryland / ICARDA temperate
FST ptn 2
FST ICARDA dryland / Italian, early CIMMYT
FST ICARDA dryland / mid CIMMYT, ICARDA
FST ICARDA dryland / late CIMMYT
FST ICARDA temperate / Italian, early CIMMYT
FST ICARDA temperate / mid CIMMYT, ICARDA
FST ICARDA temperate / late CIMMYT
FST ptn 3
FST Italian, early CIMMYT / mid CIMMYT, ICARDA
FST Italian, early CIMMYT / late CIMMYT
FST mid CIMMYT, ICARDA / late CIMMYT
FST ptn 2
FST ptns 1 and 2

## Slide 10
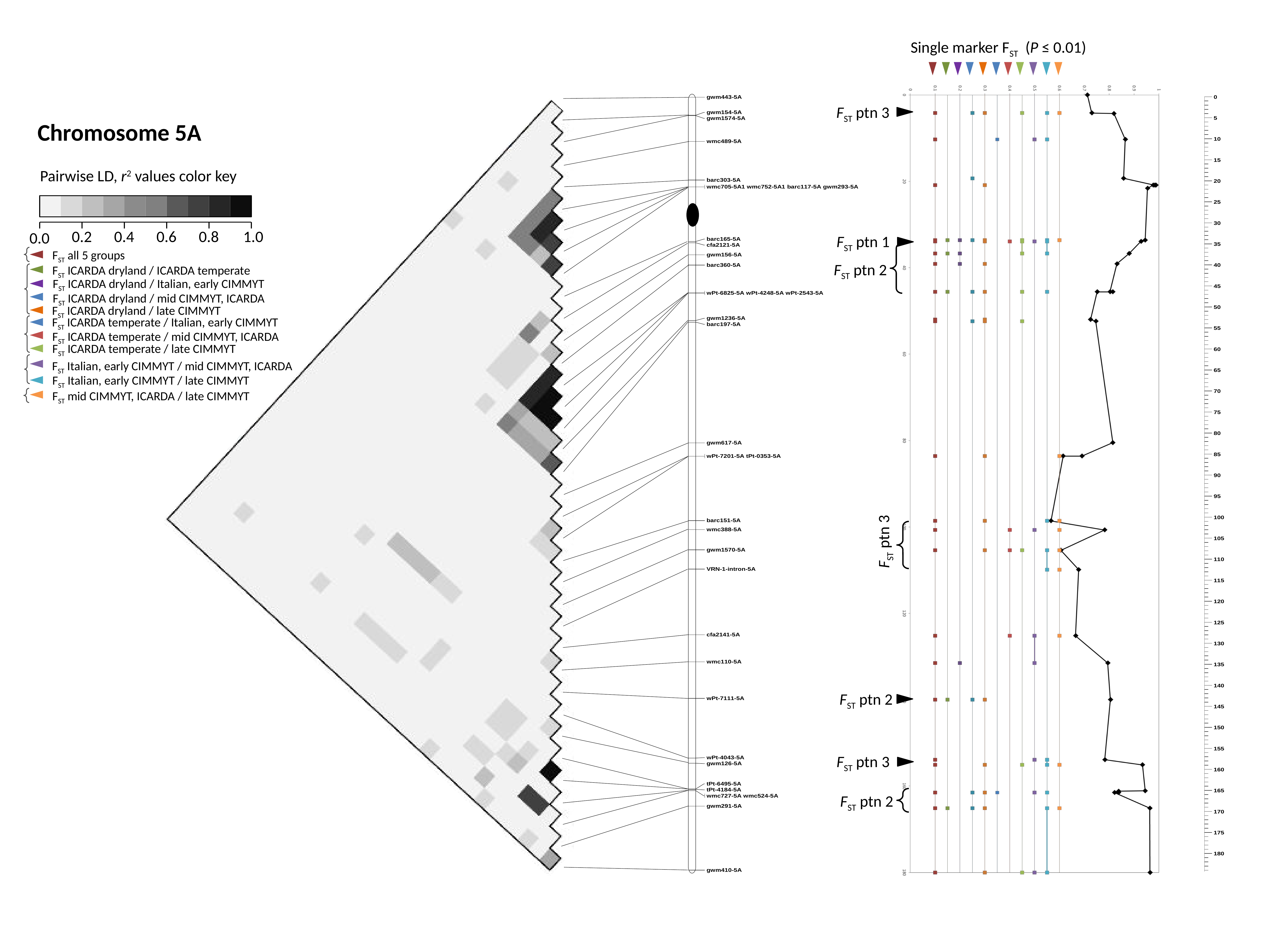

Single marker FST (P ≤ 0.01)
FST ptn 3
Chromosome 5A
Pairwise LD, r2 values color key
0.2
0.4
0.6
0.8
1.0
0.0
FST ptn 1
FST all 5 groups
FST ptn 2
FST ICARDA dryland / ICARDA temperate
FST ICARDA dryland / Italian, early CIMMYT
FST ICARDA dryland / mid CIMMYT, ICARDA
FST ICARDA dryland / late CIMMYT
FST ICARDA temperate / Italian, early CIMMYT
FST ICARDA temperate / mid CIMMYT, ICARDA
FST ICARDA temperate / late CIMMYT
FST Italian, early CIMMYT / mid CIMMYT, ICARDA
FST Italian, early CIMMYT / late CIMMYT
FST mid CIMMYT, ICARDA / late CIMMYT
FST ptn 3
FST ptn 2
FST ptn 3
FST ptn 2

## Slide 11
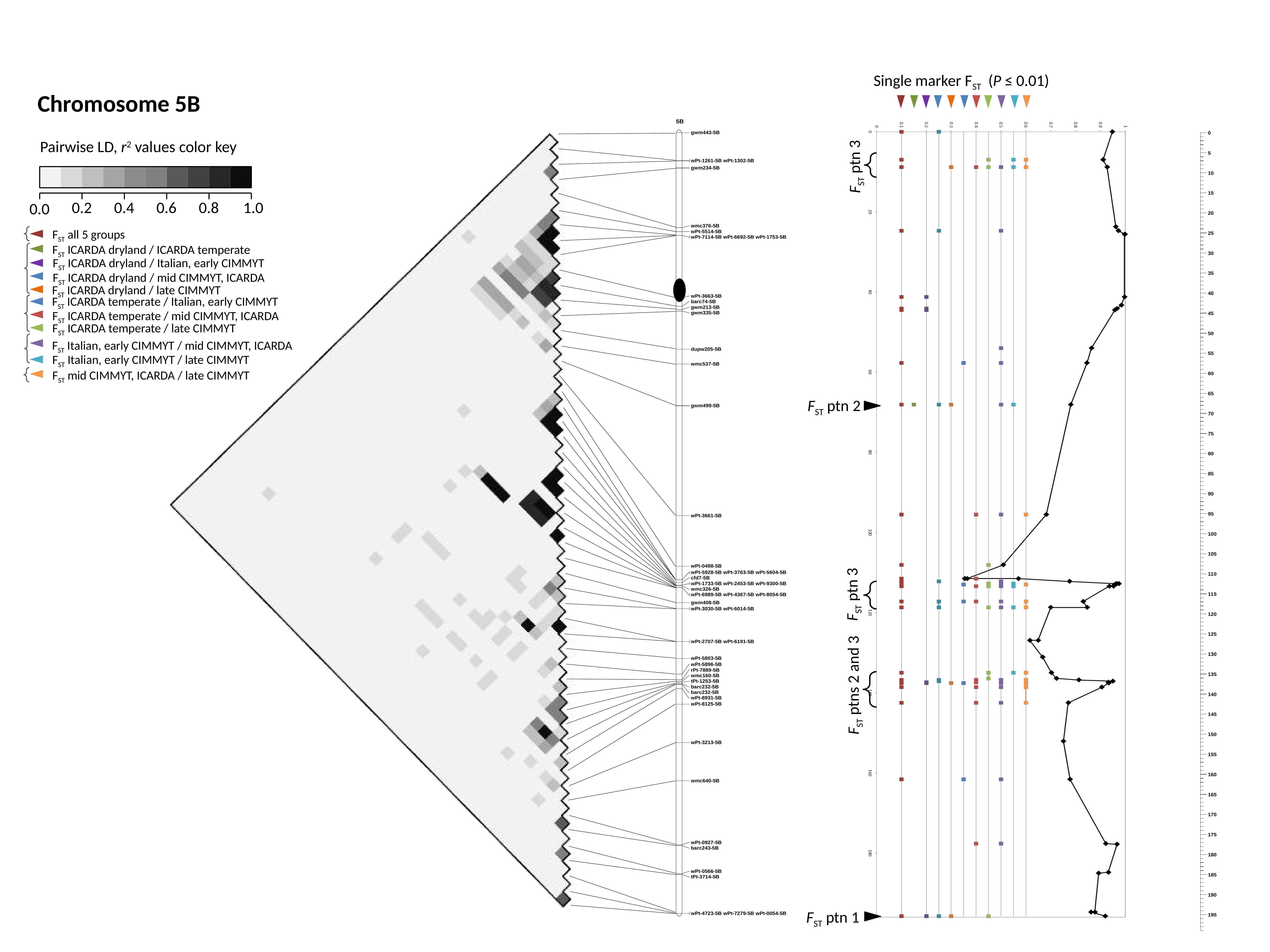

Single marker FST (P ≤ 0.01)
Chromosome 5B
Pairwise LD, r2 values color key
0.2
0.4
0.6
0.8
1.0
0.0
FST ptn 3
FST all 5 groups
FST ICARDA dryland / ICARDA temperate
FST ICARDA dryland / Italian, early CIMMYT
FST ICARDA dryland / mid CIMMYT, ICARDA
FST ICARDA dryland / late CIMMYT
FST ICARDA temperate / Italian, early CIMMYT
FST ICARDA temperate / mid CIMMYT, ICARDA
FST ICARDA temperate / late CIMMYT
FST Italian, early CIMMYT / mid CIMMYT, ICARDA
FST Italian, early CIMMYT / late CIMMYT
FST mid CIMMYT, ICARDA / late CIMMYT
FST ptn 2
FST ptn 3
FST ptns 2 and 3
FST ptn 1

## Slide 12
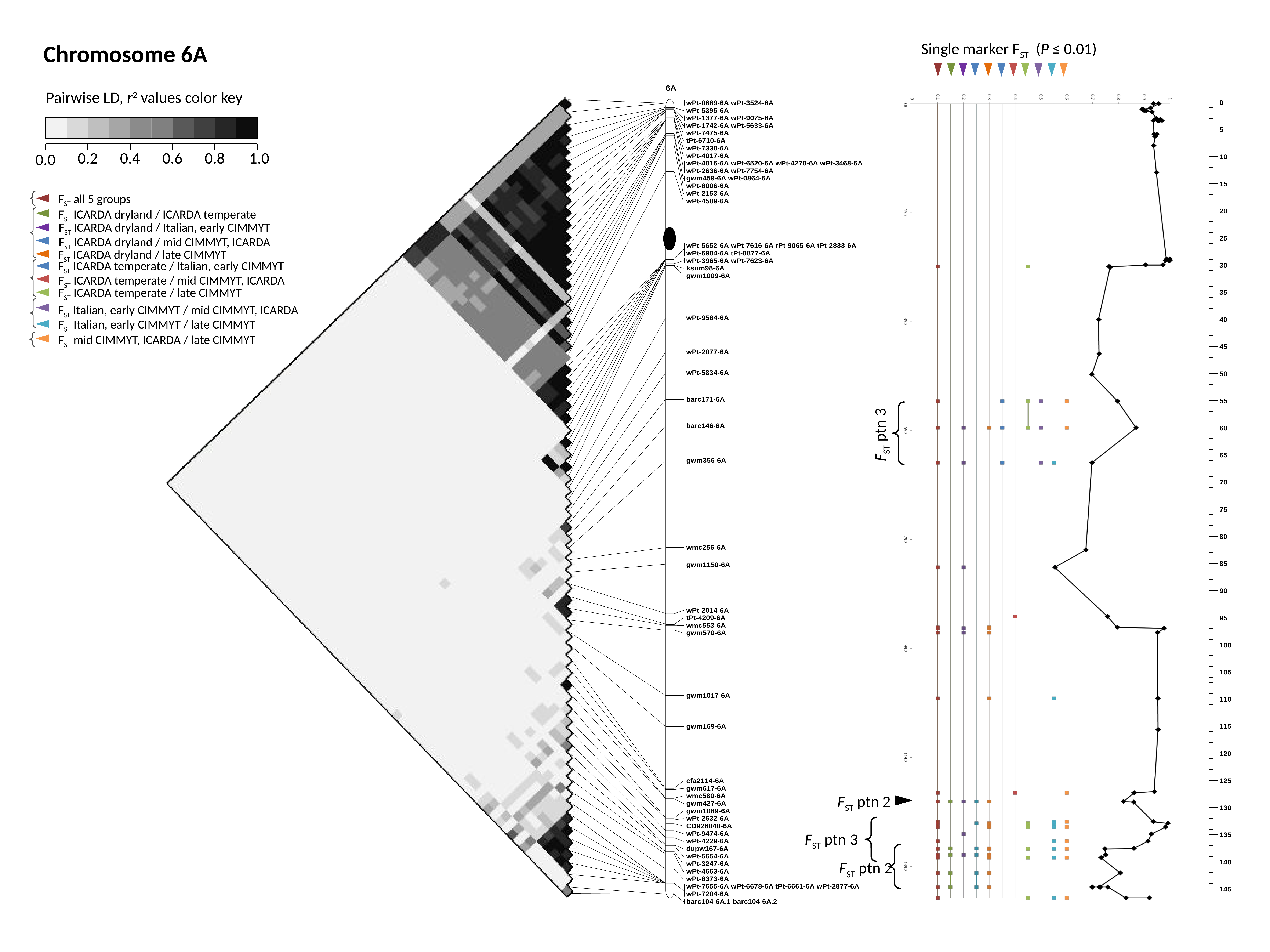

Single marker FST (P ≤ 0.01)
Chromosome 6A
Pairwise LD, r2 values color key
0.2
0.4
0.6
0.8
1.0
0.0
FST all 5 groups
FST ICARDA dryland / ICARDA temperate
FST ICARDA dryland / Italian, early CIMMYT
FST ICARDA dryland / mid CIMMYT, ICARDA
FST ICARDA dryland / late CIMMYT
FST ICARDA temperate / Italian, early CIMMYT
FST ICARDA temperate / mid CIMMYT, ICARDA
FST ICARDA temperate / late CIMMYT
FST Italian, early CIMMYT / mid CIMMYT, ICARDA
FST Italian, early CIMMYT / late CIMMYT
FST mid CIMMYT, ICARDA / late CIMMYT
FST ptn 3
FST ptn 2
FST ptn 3
FST ptn 2

## Slide 13
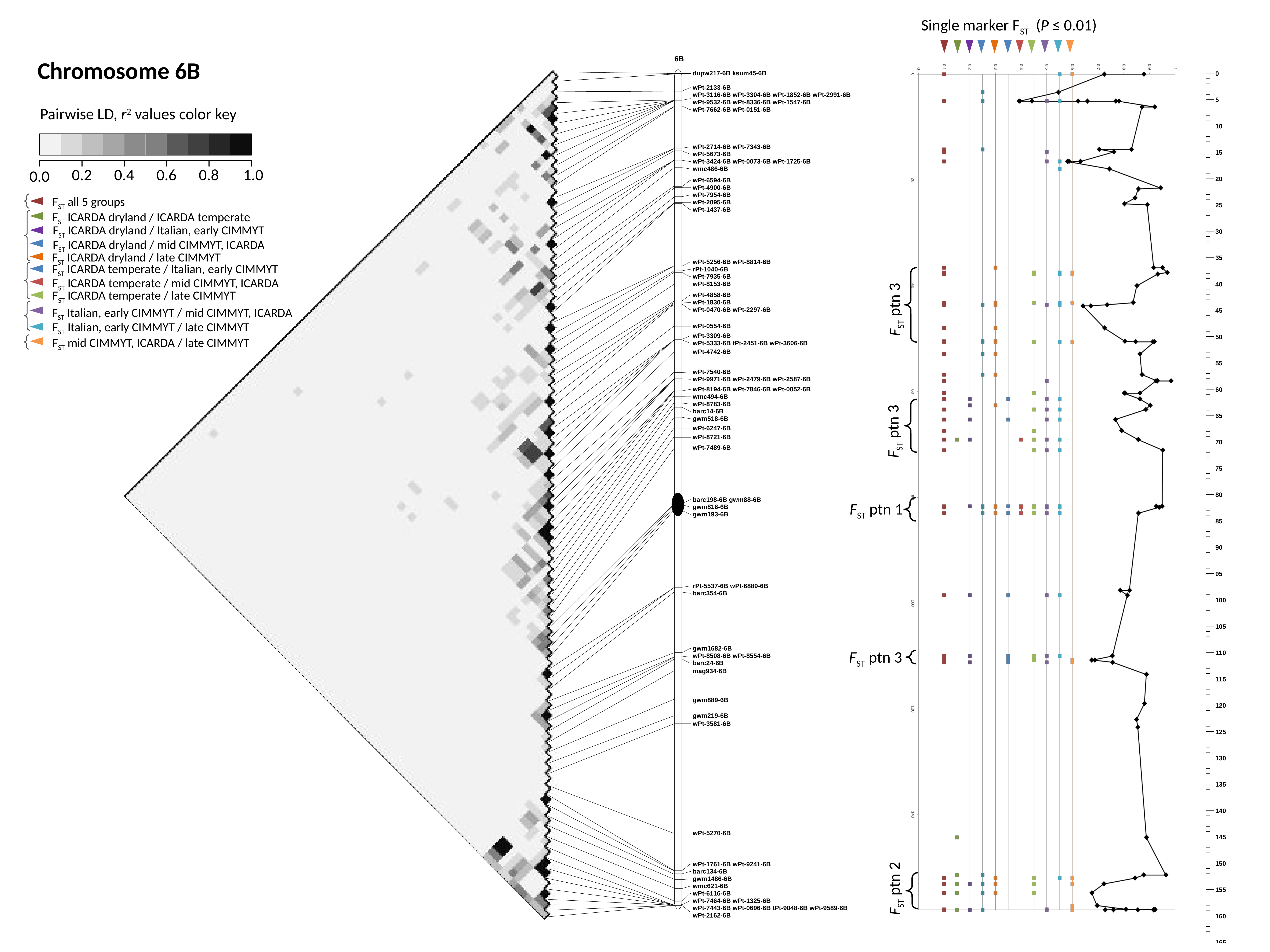

Single marker FST (P ≤ 0.01)
Chromosome 6B
Pairwise LD, r2 values color key
0.2
0.4
0.6
0.8
1.0
0.0
FST all 5 groups
FST ICARDA dryland / ICARDA temperate
FST ICARDA dryland / Italian, early CIMMYT
FST ICARDA dryland / mid CIMMYT, ICARDA
FST ICARDA dryland / late CIMMYT
FST ICARDA temperate / Italian, early CIMMYT
FST ICARDA temperate / mid CIMMYT, ICARDA
FST ICARDA temperate / late CIMMYT
FST ptn 3
FST Italian, early CIMMYT / mid CIMMYT, ICARDA
FST Italian, early CIMMYT / late CIMMYT
FST mid CIMMYT, ICARDA / late CIMMYT
FST ptn 3
FST ptn 1
FST ptn 3
FST ptn 2

## Slide 14
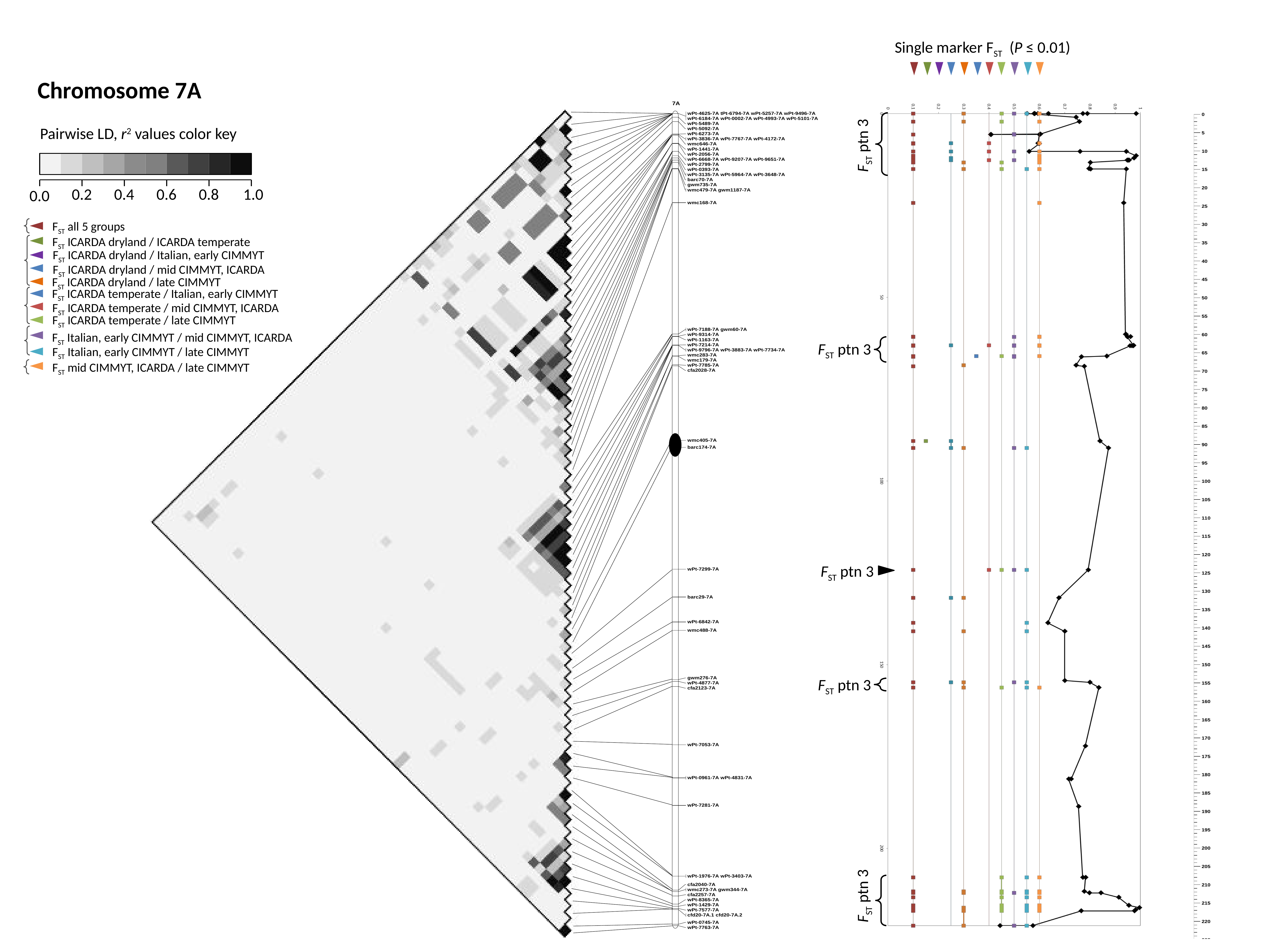

Single marker FST (P ≤ 0.01)
Chromosome 7A
Pairwise LD, r2 values color key
0.2
0.4
0.6
0.8
1.0
0.0
FST ptn 3
FST all 5 groups
FST ICARDA dryland / ICARDA temperate
FST ICARDA dryland / Italian, early CIMMYT
FST ICARDA dryland / mid CIMMYT, ICARDA
FST ICARDA dryland / late CIMMYT
FST ICARDA temperate / Italian, early CIMMYT
FST ICARDA temperate / mid CIMMYT, ICARDA
FST ICARDA temperate / late CIMMYT
FST Italian, early CIMMYT / mid CIMMYT, ICARDA
FST ptn 3
FST Italian, early CIMMYT / late CIMMYT
FST mid CIMMYT, ICARDA / late CIMMYT
FST ptn 3
FST ptn 3
FST ptn 3

## Slide 15
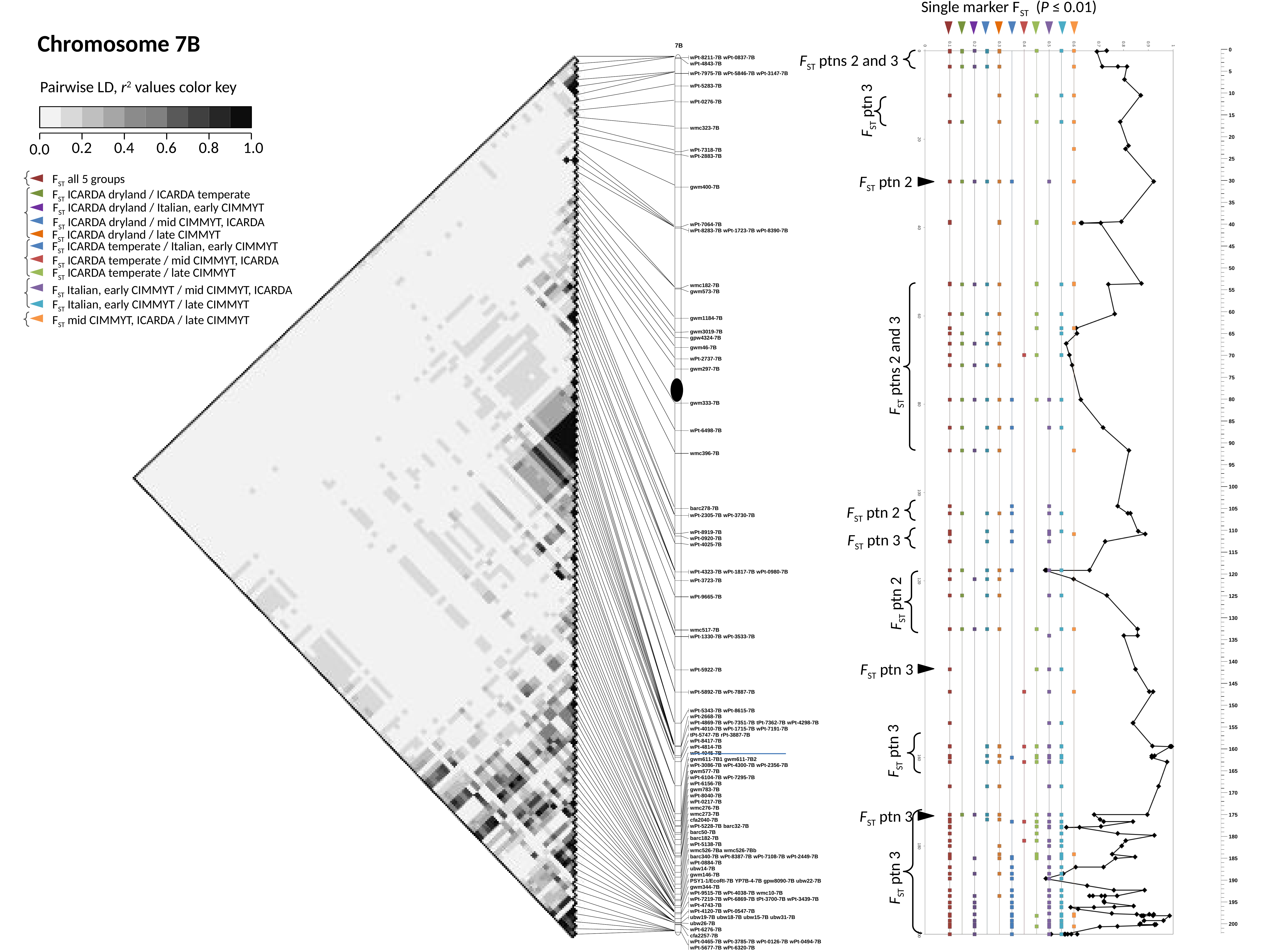

Single marker FST (P ≤ 0.01)
Chromosome 7B
Pairwise LD, r2 values color key
0.2
0.4
0.6
0.8
1.0
0.0
FST all 5 groups
FST ICARDA dryland / ICARDA temperate
FST ICARDA dryland / Italian, early CIMMYT
FST ICARDA dryland / mid CIMMYT, ICARDA
FST ICARDA dryland / late CIMMYT
FST ICARDA temperate / Italian, early CIMMYT
FST ICARDA temperate / mid CIMMYT, ICARDA
FST ICARDA temperate / late CIMMYT
FST Italian, early CIMMYT / mid CIMMYT, ICARDA
FST Italian, early CIMMYT / late CIMMYT
FST mid CIMMYT, ICARDA / late CIMMYT
FST ptns 2 and 3
FST ptn 3
FST ptn 2
FST ptns 2 and 3
FST ptn 2
FST ptn 3
FST ptn 2
FST ptn 3
FST ptn 3
FST ptn 3
FST ptn 3
