# Supplementary material for: A consensus framework map of durum wheat (Triticum durum Desf.) suitable for linkage disequilibrium analysis and genome-wide association mapping
Source: BMC Genomics. 2014 Oct 7;15(1):873. doi: 10.1186/1471-2164-15-873 (PMC4287192; doi:10.1186/1471-2164-15-873)
Supplement: Supplementary file 7 — Additional file 7: Table S2: GWAS results for heading date in the elite Durum Panel across 27 Mediterranean and Mexican environments using Ppd-A1 and Ppd-B1 representative markers as covariates in the mixed linear model. Marker-trait associations, chromosome locations and significance intervals based on the consensus map, P-values and (R 2) coefficients of determination for the chromosome regions harboring QTLs significant for heading date adjusted means from field experiments conducted on five different macro-environment areas (Southern Europe with 10 field experiments [environments], North Africa (Tunisia) with 2 envs., West Asia with 8 envs., North Africa (Morocco) with 2 envs. and Mexico with 5 envs.). A QTL was declared when a marker-wise highly significant effect (P ≤ 0.01) was detected on the adj. means for at least one macro-environmental area. R 2 values are reported in brackets next to the P-values. The number of single environments where the QTLs showed significant effects is also reported for each macro-area. Macro-area are sorted from left to right based on decreasing latitude. (DOCX 47 KB) [file 12864_2014_6782_MOESM7_ESM.docx]

**A consensus framework map of durum wheat (*Triticum durum* Desf.) suitable for linkage disequilibrium analysis and genome-wide association mapping**

**Marco Maccaferri^1,6 §^, Maria Angela Cane’^1^, Maria C Sanguineti^1^, Silvio Salvi^1^, Maria C Colalongo^1^, Andrea Massi^2^, Fran Clarke^3^, Ron Knox^3^, Curtis Pozniak^4^, John M Clarke^4^, Tzion Fahima^5^, Jorge Dubcovsky^6,7^, Steven Xu^8^, Karim Ammar^9^, Ildikó Karsai^10^, Gyula Vida^10^, Roberto Tuberosa^1^**

**Table S2.** GWAS results for heading date in the elite Durum Panel across 27 Mediterranean and Mexican environments, grouped in five main macro-environmental areas, using *Ppd-A1* and *Ppd-B1* representative markers as covariates in the mixed linear model.

**Table S2. GWAS results for heading date in the elite Durum Panel across 27 Mediterranean and Mexican environments, grouped in five main macro-environmental areas, using *Ppd-A1* and *Ppd-B1* representative markers as covariates in the mixed linear model.**

Marker-trait associations, chromosome locations and significance intervals based on the consensus map, *P*-values and (*R*^2^) coefficients of determination for the chromosome regions harboring QTLs significant for heading date adjusted means from field experiments conducted on five different macro-environmental areas (Southern Europe with 10 field experiments [environments], North Africa (Tunisia) with 2 envs., West Asia with 8 envs., North Africa (Morocco) with 2 envs. and Mexico with 5 envs.). A QTL was declared when a marker-wise highly significant effect (*P* ≤ 0.01) was detected on the adj. means for at least one macro-environmental area. *R*^2^ values are reported in brackets next to the *P*-values. The number of single environments where the QTLs showed significant effects is also reported for each macro-area. Macro-area are sorted from left to right based on decreasing latitude.

| **QTL** | **Chr** | **Representative marker** | **Position** | **Associated markers** | **LD**  **block** |  | **Macro-environmental areas** | | | | | | | | | |
| --- | --- | --- | --- | --- | --- | --- | --- | --- | --- | --- | --- | --- | --- | --- | --- | --- |
|  |  |  |  |  | **Interval** |  | **Southern Europe**  **(10 envs.)** | | **North-Africa (Tunisia)**  **(2 envs.)** | | **West Asia**  **(8 envs.)** | | **North Africa (Morocco)**  **(2 envs.)** | | **Mexico**  **(5 envs.)** | |
|  |  |  | **cM** | **No.** | **cM** |  | ***P*-value (*R*^2^)** | **Envs**  **no.** | ***P*-value (*R*^2^)** | **Envs**  **no.** | ***P*-value (*R*^2^)** | **Envs no.** | ***P*-value (*R*^2^)** | **Envs**  **no.** | ***P*-value (*R*^2^)** | **Envs**  **no.** |
| 1 | 1A | *wmc95* | 18.6 | - | - |  | 0.005 (3.23) | 5 | 0.050 (3.07) | 1 | 0.002 (4.27) | 7 | 0.046 (1.69) | 2 | 0.019 (1.66) | 3 |
| 2 | 1B | *gpw3013* | 28.7 | - | - |  | ns | 0 | ns | 0 | 0.005 (3.39) | 4 | ns | 1 | 0.006 (2.32) | 4 |
| 3 | 1B | *gwm947* | 33.3 | - | - |  | 0.016 (2.40) | 3 | ns | 0 | 0.018 (2.36) | 2 | 0.014 (2.60) | 2 | 5.09E-4 (3.71) | 5 |
| 4 | 2A1 | *barc212* | 0 | - | - |  | 0.026 (2.02) | 1 | ns | 1 | ns | 0 | ns | 1 | 0.008 (2.14) | 4 |
| 5 | 2A1 | *wmc407* | 0.4 | 3 | 2.9 |  | 0.010 (2.66) | 7 | 0.004 (5.04) | 2 | 0.007 (2.84) | 1 | ns | 7 | ns | 3 |
| 6 | 2A1 | *wmc522* | 50.1 | - | - |  | 0.002 (3.88) | 2 | ns | 0 | 0.029 (2.00) | 5 | ns | 0 | 0.044 (1.22) | 2 |
| 7 | 2A1 | *gwm372* | 72.1 | - | - |  | 0.024 (2.07) | 2 | ns | 0 | 0.002 (4.16) | 5 | 0.009 (2.97) | 2 | 0.020 (1.61) | 1 |
| 8 | 2A2 | *tPt-2163* | 20.8 | 3 | 0 |  | 0.001 (4.28) | 4 | 0.003 (4.93) | 2 | 0.018 (2.36) | 2 | ns | 0 | ns | 2 |
| 9 | 2B2 | *wmc25* | 19.6 | - | - |  | 0.018 (2.27) | 1 | ns | 0 | 0.006 (3.27) | 4 | 0.050 (1.16) | 1 | 0.003 (2.67) | 5 |
| 10 | 2B2 | *wmc243* | 24.5 |  |  |  | ns | 0 | 0.006 (4.40) | 2 | 0.045 (1.70) | 3 | ns | 0 | ns | 1 |
| 11 | 3A | *cfd79-3A* | 0 | - | - |  | 0.018 (2.30) | 4 | 0.031 (2.69) | 2 | ns | 4 | 0.030 (2.01) | 1 | 0.007 (2.17) | 2 |
| 12 | 3A | *wPt-1652* | 3.9 | 3 | 1.8 |  | ns | 1 | ns | 0 | ns | 0 | ns | 0 | 0.002 (2.78) | 5 |
| 13 | 3A | *wmc428* | 45 | - | - |  | 0.007 (2.98) | 4 | ns | 0 | 0.01 (2.72) | 5 | 0.016 (2.50) | 1 | 0.004 (2.48) | 5 |
| 14 | 3A | *wPt-9160* | 150.9 | 1 | 2.9 |  | 0.003 (3.54) | 7 | ns | 1 | ns | 3 | ns | 1 | 0.026 (1.48) | 2 |
| 15 | 3B | *gwm1034* | 1.5 | 1 | 1.5 |  | 0.007 (2.98) | 6 | ns | 1 | 0.001 (4.49) | 5 | 0.023 (2.22) | 1 | 0.024 (1.53) | 1 |
| 16 | 3B | *cfd79-3B* | 24.3 | - | - |  | 0.002 (3.78) | 6 | 0.003 (5.12) | 2 | 0.007 (3.90) | 8 | ns | 0 | 0.015 (1.78) | 3 |

(continued)

**Table S2 (continued)**

| **QTL** | **Chr** | **Representative marker** | **Position** | **Associated markers** | **LD**  **block** |  | **Macro-environments** | | | | | | | | | |
| --- | --- | --- | --- | --- | --- | --- | --- | --- | --- | --- | --- | --- | --- | --- | --- | --- |
|  |  |  |  |  | **Interval** |  | **Southern Europe**  **(10 envs.)** | | **North-Africa (Tunisia)**  **(2 envs.)** | | **West Asia**  **(8 envs.)** | | **North Africa (Morocco)**  **(2 envs.)** | | **Mexico**  **(5 envs.)** | |
|  |  |  | **cM** | **No.** | **cM** |  | **P-value (*R*^2^)** | **Envs. no.** | ***P*-value (*R*^2^)** | **Envs (no.)** | ***P*-value (*R*^2^)** | **Envs no.** | ***P*-value (*R*^2^)** | **Envs**  **no.** | ***P*-value (*R*^2^)** | **Envs**  **no.** |
| 17 | 3B | *ksm45* | 41.8 | - | - |  | 0.003 (1.88) | 1 | 0.002 (5.47) | 2 | ns | 1 | 0.005 (3.42) | 2 | ns | 0 |
| 18 | 3B | *wmc808* | 45.7 | - | - |  | 0.011 (2.63) | 2 | 0.008 (3.98) | 1 | 0.008 (3.00) | 1 | ns | 0 | 0.038 (1.30) | 1 |
| 19 | 3B | *wPt-9510* | 87.8 | 5 | 10.8 |  | ns | 4 | ns | 0 | ns | 0 | 0.008 (3.06) | 1 | 0.004 (2.44) | 5 |
| 20 | 3B | *wPt-2698* | 162.9 | 1 | 2.5 |  | 0.015 (2.44) | 6 | 0.039 (2.43) | 2 | 0.005 (3.38) | 5 | 0.005 (3.42) | 2 | 0.014 (1.83) | 4 |
| 21 | 3B | *wPt-9989* | 207.8 | 5 | 8.5 |  | ns | - | 0.008 (4.01) | 2 | ns | 0 | ns | 0 | ns | 0 |
| 22 | 4A | *wmc617* | 37.9 | - | - |  | 0.034 (1.82) | 1 | ns | 0 | 0.050 (1.57) | 2 | 0.010 (1.54) | 1 | 0.023 (1.56) | 2 |
| 23 | 4B | *wPt-4931* | 23.6 | 2 | 0.3 |  | 0.008 (2.83) | 6 | ns | 0 | 0.022 (2.31) | 4 | ns | 0 | ns | 0 |
| 24 | 5A | *barc117* | 20.9 | 2 | 1.6 |  | ns | 1 | 0.008 (3.96) | 2 | ns | 1 | ns | 0 | ns | 0 |
| 25 | 5A | *barc151* | 98.6 | - | - |  | 0.001 (2.18) | 3 | ns | 0 | 0.012 (2.65) | 5 | ns | 1 | 0.019 (1.46) | 2 |
| 26 | 5A | *wmc110* | 131.5 | 2 | 0 |  | ns | 1 | ns | 1 | 0.035 (1.88) | 2 | 0.050 (1.64) | 1 | 0.004 (2.46) | 5 |
| 27 | 5A | *gwm126* | 155.1 | - | - |  | ns | 0 | 0.032 (2.64) | 1 | ns | 2 | 0.029 (2.06) | 1 | 0.015 (1.80) | 5 |
| 28 | 5A | *wmc727* | 161.5 | - | - |  | ns | 1 | ns | 0 | ns | 0 | 0.031 (1.99) | 1 | 0.009 (2.02) | 3 |
| 29 | 5A | *gwm291* | 165.1 | - | - |  | 0.033 (1.85) | 7 | ns | 0 | 0.010 (2.82) | 4 | 0.004 (3.68) | 1 | 0.022 (1.57) | 3 |
| 30 | 5B1 | *gwm234* | 8.8 | - | - |  | ns | 2 | 0.021 (3.09) | 1 | ns | 2 | 0.011 (2.78) | 1 | 6.12E-4 (3.60) | 5 |
| 31 | 5B2 | *wmc326* | 113.0 | - | - |  | 0.026 (2.02) | 3 | ns | 0 | 0.006 (3.22) | 4 | 0.029 (2.04) | 2 | ns | 0 |
| 32 | 6A | *wmc256* | 81.1 | - | - |  | 0.004 (3.35) | 7 | 0.009 (3.99) | 2 | 0.006 (3.12) | 4 | 0.030 (1.87) | 2 | 0.002 (3.00) | 5 |

**(continued)**

**Table S2 (continued)**

| **QTL** | **Chr** | **Representative marker** | **Position** | **Associated markers** | **LD**  **block** |  | **Macro-environments** | | | | | | | | | |
| --- | --- | --- | --- | --- | --- | --- | --- | --- | --- | --- | --- | --- | --- | --- | --- | --- |
|  |  |  |  |  | **Interval** |  | **Southern Europe**  **(10 envs.)** | | **North-Africa (Tunisia)**  **(2 envs.)** | | **West Asia**  **(8 envs.)** | | **North Africa (Morocco)**  **(2 envs.)** | | **Mexico**  **(5 envs.)** | |
|  |  |  | **cM** | **No.** | **cM (range)** |  | ***P*-value (*R*^2^)** | **Envs. no.** | ***P*-value (*R*^2^)** | **Envs. no.** | ***P*-value (*R*^2^)** | **Envs. no.** | ***P*-value (*R*^2^)** | **Envs. no.** | ***P*-value (*R*^2^)** | **Envs. no.** |
| 33 | 6A | *gwm169* | 114.1 | - | - |  | 0.010 (2.70) | 6 | 0.021 (3.05) | 1 | 0.035 (2.49) | 4 | ns | 0 | ns | 0 |
| 34 | 6B | *wPt-9532* | 5.1 | 1 | 0 |  | 0.009 (2.83) | 4 | 0.004 (4.81) | 2 | 0.022 (2.22) | 1 | 0.004 (3.57) | 2 | ns | 0 |
| 35 | 6B | *wPt-0470* | 43.8 | 1 | 0 |  | 0.050 (1.44) | 3 | 0.010 (3.55) | 2 | 0.024 (2.15) | 3 | 0.042 (1.78) | 1 | ns | 0 |
| 36 | 6B | *gwm518* | 65.3 | - | - |  | 0.050 (1.02) | 0 | ns | 0 | ns | 0 | ns | 0 | ns | 0 |
| 37 | 6B | *gwm88* | 81.7 | - | - |  | 0.050 (1.27) | 2 | ns | 0 | 0.002 (4.29) | 3 | ns | 0 | ns | 0 |
| 38 | 6B | *gwm816* | 81.9 | 1 | 1.1 |  | ns | 1 | ns | 1 | ns | 1 | ns | 0 | 0.004 (2.58) | 3 |
| 39 | 6B | *gwm1682* | 110.0 | - | - |  | ns | 2 | ns | 1 | ns | 1 | ns | 0 | 0.003 (2.61) | 5 |
| 40 | 6B | *gwm889* | 119.0 | - | - |  | ns | 1 | 0.046 (2.27) | 1 | 0.003 (3.84) | 3 | 0.050 (1.35) | 1 | ns | 0 |
| 41 | 6B | *wmc621* | 154.8 | - | - |  | ns | 1 | 0.030 (2.71) | 1 | 0.050 (1.45) | 3 | 0.040 (1.80) | 1 | 0.007 (2.20) | 4 |
| 42 | 7A | *wPt-9651* | 10.3 | - | - |  | 0.050 (1.54) | 1 | ns | 0 | ns | 2 | 0.029 (2.04) | 1 | 0.004 (2.44) | 4 |
| 43 | 7A | *cfa2028* | 68.8 | 1 | 0.3 |  | ns | 1 | ns | 0 | ns | 0 | ns | 0 | 0.009 (2.04) | 3 |
| 44 | 7A | *wmc405* | 89.1 | 1 | 1.9 |  | 0.040 (1.72) | 3 | ns | 0 | ns | 1 | ns | 1 | 0.013 (1.85) | 3 |
| 45 | 7A | *wPt-7053* | 172.1 | - | - |  | ns | 2 | ns | 0 | ns | 1 | ns | 0 | ns | 0 |
| 46 | 7B | *wPt-1723* | 39.0 | 3 | 0.3 |  | ns | 3 | 0.022 (2.99) | 2 | 0.029 (2.00) | 3 | 0.019 (2.33) | 1 | 0.050 (1.06) | 0 |
| 47 | 7B | *wPt-8615* | 152.2 | 1 | 0 |  | 0.004 (3.44) | 5 | ns | 0 | 0.008 (3.30) | 3 | 0.036 (1.88) | 1 | ns | 1 |
| 48 | 7B | *cfa2040* | 182.8 | - | - |  | 0.002 (3.78) | 3 | 0.004 (4.73) | 2 | ns | 1 | ns | 1 | 0.029 (1.43) | 2 |
| 49 | 7B | *barc182* | 187.4 | 1 | 0 |  | 0.010 (2.37) | 3 | ns | 1 | ns | 1 | 0.035 (1.88) | 1 | ns | 1 |
| 50 | 7B | *wPt-7108* | 191.3 | 3 | 0 |  | 0.021 (2.42) | 4 | 0.033 (2.64) | 1 | ns | 2 | 0.036 (2.57) | 1 | ns | 0 |
